# Development of mumps virus-specific sialidase imaging probes through chemical modifications of sialic acid

**DOI:** 10.1038/s41598-025-26190-y

**Published:** 2025-11-25

**Authors:** Yutaka Narimichi, Tadanobu Takahashi, Yuuki Kurebayashi, Tadamune Otsubo, Kiyoshi Ikeda, Yu Saito, Akira Minami, Hideyuki Takeuchi

**Affiliations:** 1 Department of Biochemistry, School of Pharmaceutical Sciences, University of Shizuoka, 52-1 Yada, Suruga-ku, Shizuoka-shi, Shizuoka 422-8526 Japan; 2 Department of Organic Chemistry, School of Pharmaceutical Sciences, Hiroshima International University, 5-1-1 Hirokoshinkai, Kure-shi, Hiroshima 737-0112 Japan; 3 Department of Functional Morphology, Faculty of Pharmacy, Juntendo University, 6-8-1 Hinode, Urayasu-shi, Chiba 279-0013 Japan

**Keywords:** Biochemistry, Biotechnology, Microbiology

## Abstract

Some viruses, such as mumps virus (MuV), possess sialidases that cleave terminal sialic acids, primarily *N*-acetylneuraminic acid (Neu5Ac), from glycans. Previously, we developed a sialidase fluorescent imaging probe (BTP3-Neu5Ac) and visualized cells infected with sialidase–expressing viruses. However, BTP3-Neu5Ac could not specifically identify these viruses, as it reacted with a wide range of sialidases from viruses, bacteria, and mammals. Here, to confer specificity of BTP3-Neu5Ac to MuV sialidase, which features a hydrophobic cavity extending toward the C5 position of bound Neu5Ac, we evaluated BTP3-Neu5Ac derivatives containing hydrophobic groups at the C5 position of Neu5Ac moiety across various viral sialidases, including influenza A and B viruses. Linear *N*-acyl groups with 5–8 carbon atoms at this position improved specificity for MuV sialidase. These derivatives might enable precise identification and diagnosis of MuV infections. Our findings demonstrate that chemical modifications to Neu5Ac in BTP3-Neu5Ac can effectively modulate its specificity for viral sialidases.

**Supplementary Information:**

The online version contains supplementary material available at 10.1038/s41598-025-26190-y.

## Introduction

Sialidase (enzyme code 3.2.1.18) is an exohydrolase that cleaves terminal sialic acids from glycans. Sialidases are found in a wide range of hosts, including viruses, bacteria, and mammals. *Orthorubulavirus parotitidis*, *Respirovirus laryngotracheitidis*, *Respirovirus pneumoniae*, *Alphainfluenzavirus influenzae*, and *Betainfluenzavirus influenzae* are commonly known as the mumps virus (MuV), human parainfluenza virus type 1 (hPIV1), human parainfluenza virus type 3 (hPIV3), influenza A virus (IAV), and influenza B virus (IBV), respectively. These are human epidemic pathogenic viruses possessing sialidase activity. MuV and hPIV belong to the *Paramyxoviridae* family and possess hemagglutinin-neuraminidase protein (HN), which exhibits sialidase activity. IAV and IBV belong to the *Orthomyxoviridae* family and possess neuraminidase protein (NA), which reveals sialidase activity^1^. MuV is associated with various pathogenic conditions, including aseptic meningitis, encephalitis, inflammation of testicles (orchitis) and ovaries (oophoritis), and severe sequelae such as deafness, infertility, subfertility, and pancreatitis^2^. Mumps-associated deafness is a severe sequela with a poor prognosis, posing a significant risk to children, adolescents, and adults, with an overall incidence of 0.15%^3^. First-time MuV infections in adolescents and adults can potentially result in infertility or subfertility due to orchitis and oophoritis^4,5^. hPIV, IAV, and IBV primarily cause respiratory syndromes. hPIV, predominantly hPIV1 and hPIV3, accounts for approximately 20% of viral pneumonia cases in children under the age of five^6–8^. The global spread of IAV and IBV affects up to 10% of the world’s population annually, resulting in more than 15 million cases of infection. In Japan alone, approximately 15,000 hospitalizations occur during a single influenza season^9,10^. *N*-acetylneuraminic acid (Neu5Ac), the primary molecular species of sialic acids, is located at the terminus of glycans and serves as a receptor for these viruses, facilitating their attachment to host cell surfaces. Viral sialidase activities promote the release of progeny viruses from host cells by preventing the binding of viruses to Neu5Ac^11–13^.

**Fig. 1:**
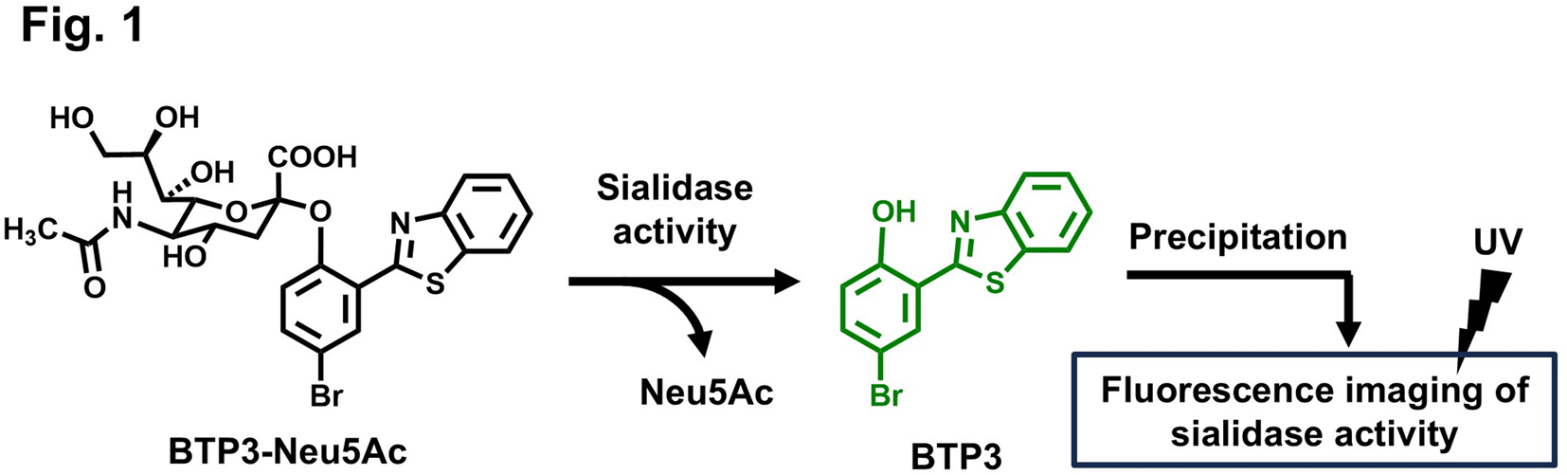
Fluorescence imaging of sialidase activity using BTP3-Neu5Ac. Enzymatic cleavage of Neu5Ac from BTP3-Neu5Ac by sialidase generates the hydrophobic fluorophore BTP3, which precipitates locally at the site of activity, thereby enabling spatially resolved fluorescence imaging under ultraviolet (UV) irradiation.

In the our previous study, we developed a sialidase fluorescent imaging probe, *N*-acetyl-2-*O*-[2-(2-benzothiazolyl)-4-bromophenyl]-α-neuraminic acid (BTP3-Neu5Ac). Sialidase activity cleaves BTP3-Neu5A into Neu5Ac and a hydrophobic fluorescent compound, 2-(benzothiazol-2-yl)-4-bromophenol (BTP3). BTP3 is pH-insensitive and emits green fluorescence under ultraviolet (UV) irradiation with a broader Stokes shift. Histochemically, BTP3 exhibits sialidase activity by precipitating at the locations where sialidase activity is observed (Fig. 1). BTP3-Neu5Ac enables easy and rapid fluorescent imaging of cells infected with MuV^14^, hPIV1 and hPIV3^15^, and IAV and IBV^16^, without requiring cell fixation and antiviral antibodies. It can also be used to measure viral infection titers and isolate viruses from live fluorescent cells. For IAV detection, viruses concentrated on an ultrafiltration membrane and visualized using BTP3-Neu5Ac within approximately 15 min demonstrated higher sensitivity compared to commercial influenza diagnostic kits employing anti-viral nucleoprotein antibodies. Furthermore, this BTP3-Neu5Ac-based approach can help detect resistance to anti-influenza drugs targeting the inhibition of viral sialidase^17,18^. Nevertheless, because BTP3-Neu5Ac reacts with several sialidases from viruses, bacteria, and mammals^19^, it cannot be used for the specific identification of viruses. Therefore, if BTP3-Neu5Ac derivatives with high specificity for particular viruses can be developed, they would serve as valuable tools for diagnosing viral infections in clinical settings and enabling easy and rapid fluorescence imaging of virus-infected cells from large numbers of samples for epidemiological investigations in various environmental and hygiene conditions.

**Fig. 2:**
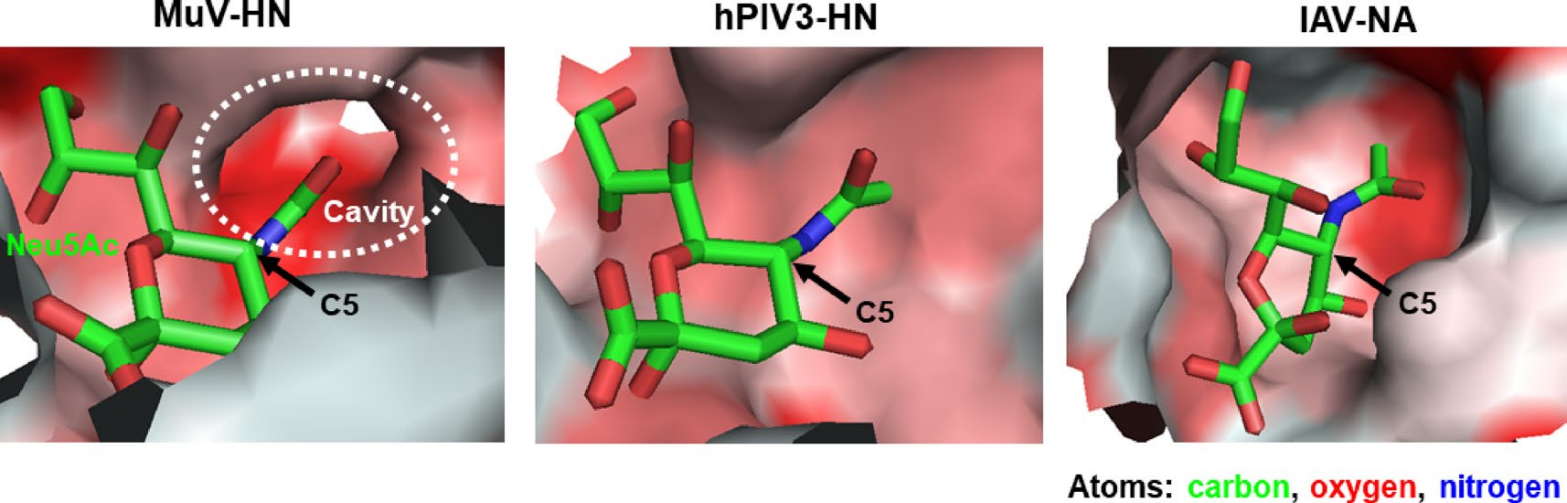
Three-dimensional structure of Neu5Ac-binding sites in MuV-HN, hPIV3-HN, and IAV-NA. Structural data of MuV-HN (PDB ID 5B2D), hPIV3-HN (PDB ID 1V3C), and IAV-NA (PDB ID 2BAT) were obtained from the Protein Data Bank and displayed using the PyMOL Molecular Graphics System version 1. 1r. 1 (Delano Scientific LLC.). Atoms within 8 Å of the nitrogen atom of Neu5Ac were selected and displayed as a surface model, with its hydrophobic regions highlighted in red. Neu5Ac is depicted as a stick model, with carbon, oxygen, and nitrogen atoms represented in green, red, and blue, respectively. Arrows indicate the C5 position in Neu5Ac. The hydrophobic cavity is demarcated by a white dotted circle.

As the active site of sialidases directly recognizes the structure of Neu5Ac, chemical modifications to Neu5Ac might significantly influence the reactivity and specificity of these enzymes. For instance, *O*-methylation at the C4 and C7 positions of Neu5Ac in a sialidase substrate has been shown to improve specificity for IAV and IBV sialidases^20^. Moreover, 4-guanidino-2,4-dideoxy-2,3-dehydro-*N*-acetylneuraminic acid, commonly known as the anti-influenza drug zanamivir, was developed as a highly specific competitive inhibitor for IAV and IBV sialidases^21^. To confer enzymatic reactivity of BTP3-Neu5Ac to specific viruses, we explored the effects of chemical modifications to the Neu5Ac moiety in BTP3-Neu5Ac on enzymatic reactivity and specificity for MuV, hPIV, IAV, and IBV. Structural analysis of the MuV-HN protein co-crystallized with α2,3-sialyllactose^22^ revealed a hydrophobic cavity extending toward the *N*-acetyl group at the C5 position of Neu5Ac. In contrast, no such hydrophobic cavity was detected in the protein structures of hPIV3-HN complexed with Neu5Ac^23^ or IAV-NA complexed with Neu5Ac^24^ (Fig. 2). In the present study, to develop BTP3-Neu5Ac derivatives with high specificity for MuV-HN using this cavity, we compared the reactivity and specificity of derivatives containing hydrophobic groups at the C5 position of Neu5Ac against the sialidases of MuV, hPIV, IAV, and IBV.

## Results

### Reactivity of BTP3-Neu5Ac derivatives containing hydrophobic groups at the C5 position of the Neu5Ac moiety for viral sialidases

**Fig. 3:**
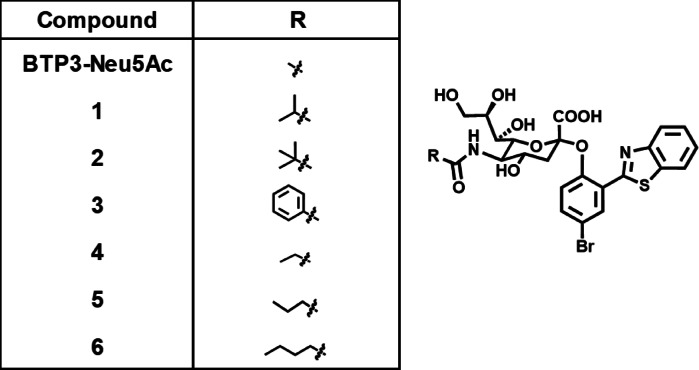
Chemical structures of BTP3-Neu5Ac and Compounds **1**–**6**.

**Fig. 4:**
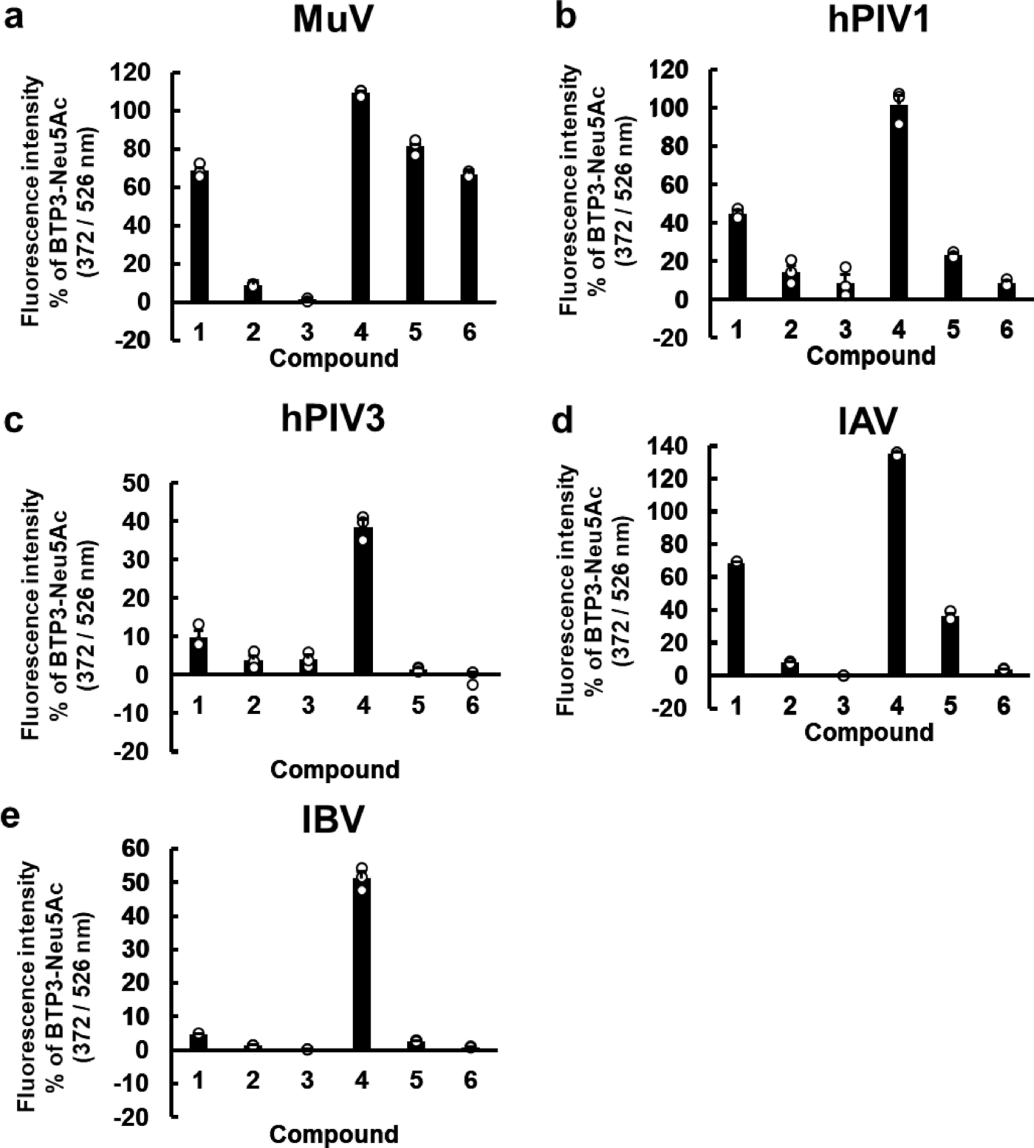
Comparison of Compounds **1**–**6** with BTP3-Neu5Ac for sialidase activity of MuV-HN, hPIV1-HN, hPIV3-HN, IAV-NA, and IBV-NA. (**a–e**) 293T cells were transfected with expression vectors for MuV-HN (**a**), hPIV1-HN (**b**), hPIV3-HN (**c**), IAV-NA (PR8 strain) (**d**), and IBV-NA (**e**). Each set of HN– or NA–expressing cells was incubated with 50 µM of BTP3-Neu5Ac or each compound at 37 °C for 20 min. Fluorescence intensity with Compounds **1**–**6** is expressed as a percentage relative to that obtained with BTP3-Neu5Ac (set as 100%).

The Neu5Ac-binding site in MuV-HN, hPIV3-HN, and IAV-NA (Supplementary Fig. 1) is believed to significantly affect the activity of viral sialidases. Although BTP3-Neu5Ac reacts with a wide range of sialidases, including those from viruses, bacteria, and mammals, it does not exhibit specificity for any particular sialidase. Nevertheless, chemical modifications to Neu5Ac might alter the reactivity and specificity of this enzyme for individual viral sialidases. In MuV-HN, a hydrophobic cavity extends toward the C5 position of the Neu5Ac moiety within the binding site. This structural feature is absent in hPIV3-HN or IAV-NA (Fig. 2), suggesting that introducing hydrophobic groups at the C5 position of the Neu5Ac moiety could improve the specificity of BTP3-Neu5Ac-based probes for MuV sialidase. To explore this hypothesis, we synthesized BTP3-Neu5Ac derivatives containing *N*-isobutanoyl, *N*-pivaloyl, *N*-benzoyl, *N*-propanoyl, *N*-butanoyl, and *N*-pentanoyl groups, designated as Compounds **1**, **2**, **3**, **4**, **5**, and **6**, respectively (Fig. 3). MuV, hPIV, IAV, and IBV are human pathogenic viruses possessing sialidase activity and are known to occasionally cause co-infections^8,25,26^. In the present study, we investigated the enzymatic reactivity of BTP3-Neu5Ac derivatives with the sialidases of MuV, hPIV, IAV, and IBV. For hPIV, we focused on hPIV1 and hPIV3, which are the most frequently detected strains of hPIV^7,27^. The BTP3 fluorescence of Compounds **1**–**6** was measured after incubating genetically viral sialidase–expressing cells with each compound for 20 min at 37 °C and compared with the fluorescence of BTP3-Neu5A, which serves as a standard substrate for these viral sialidases. Compound **6** exhibited remarkable reactivity exclusively for MuV sialidase (Fig. 4a) but showed minimal reactivity for the remaining sialidases (Fig. 4b–e). These findings indicated that Compound **6** possessed high specificity for MuV sialidase.

Next, we investigated whether the observed enzymatic activity is specifically attributable to each BTP3-Neu5Ac derivative; and whether sialidase activity can be quantitatively evaluated without substrate saturation, indicated by a constant ratio of fluorescence intensity between each derivative and BTP3-Neu5Ac. Thus, we investigated the enzymatic reactivity of Compounds **1**–**3** by incubating viral sialidase–expressing cells with varying concentrations of each compound for 20 min at 37 °C (Supplementary Fig. 2a–g). All the tested viral sialidases demonstrated concentration-dependent fluorescence intensities when incubated with varying concentrations of BTP3-Neu5Ac as a standard substrate (Supplementary Fig. 3a–g). Compound **1** showed high reactivity toward MuV (Supplementary Fig. 2a). The reactivity of hPIV1 with Compound **1** was greater than that observed with BTP3-Neu5Ac, exceeding by 100% relative fluorescence intensity. The reactivity appeared to reach saturation as the relative sialidase activity with BTP3-Neu5Ac decreased with increasing substrate concentrations (Supplementary Fig. 2b). For IAV, we analyzed three sialidases: N1 NAs from A/PR/8/1934 H1N1 strain (IAV PR8, Supplementary Fig. 2d) and A/Shizuoka/738/2009 H1N1 strain (IAV S738, Supplementary Fig. 2f) and N2 NA from A/Memphis/1/1971 H3N2 strain (IAV M71, Supplementary Fig. 2g). IAV S738, which features a histidine-to-tyrosine substitution at amino acid position 275 in N1 NA, is resistant to oseltamivir, a widely used NA inhibitor for influenza treatment^18^. We detected similar reactivity between IAV PR8 and IAV S738 (Supplementary Fig. 2d,f). IAV M71 demonstrated lower reactivity to Compound **1** than IAV PR8 and IAV S738 (Supplementary Fig. 2g). The reactivity of Compound **1** remained unaffected by the oseltamivir-resistant mutation. The reactivity of N2 NA with Compound **1** appeared to be lower than that of N1 NAs. Compound **2** demonstrated minimal or no reactivity with any of the tested viral sialidases, except for hPIV1. Compound **3** exhibited no detectable reactivity with any of the tested viral sialidases (Fig. 4a–e, Supplementary Fig. 2a–g). These findings suggested that bulky hydrophobic groups, such as pivaloyl and benzoyl moieties at the C5 position of the Neu5Ac moiety, are not suitable for recognition as substrates by viral sialidases.

### Long linear *N*-acyl groups at the C5 position of the Neu5Ac moiety in BTP3-Neu5Ac improve the specificity for MuV sialidase

**Fig. 5:**
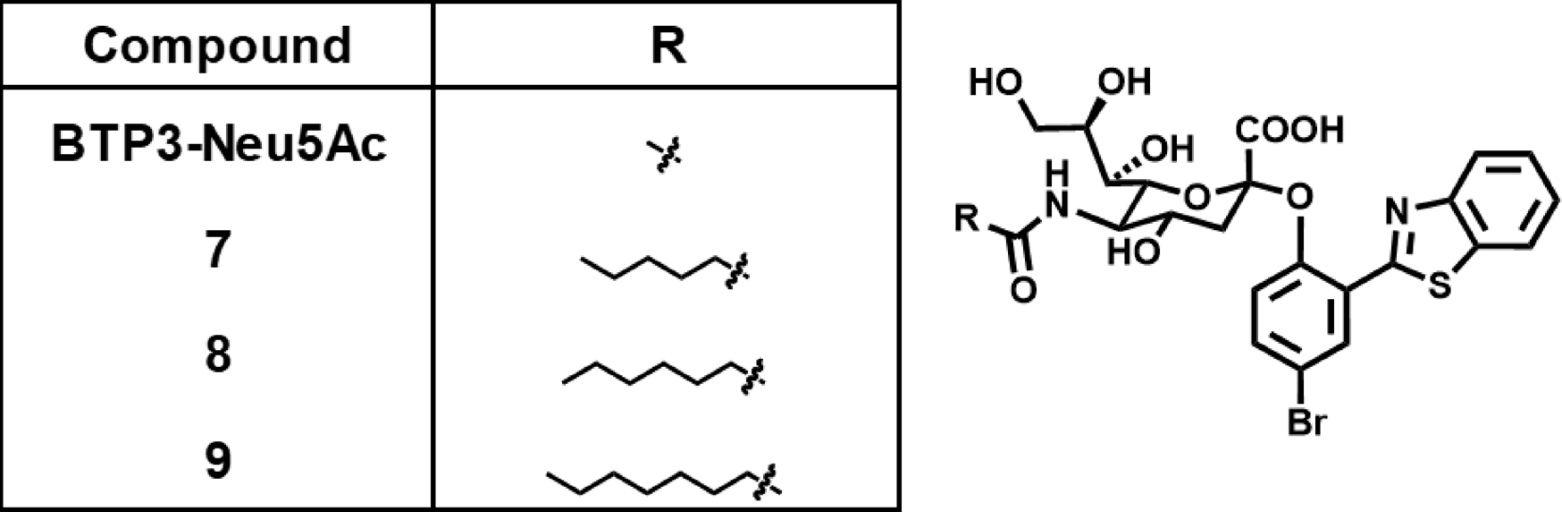
Chemical structures of Compounds **7**–**9**.

**Fig. 6:**
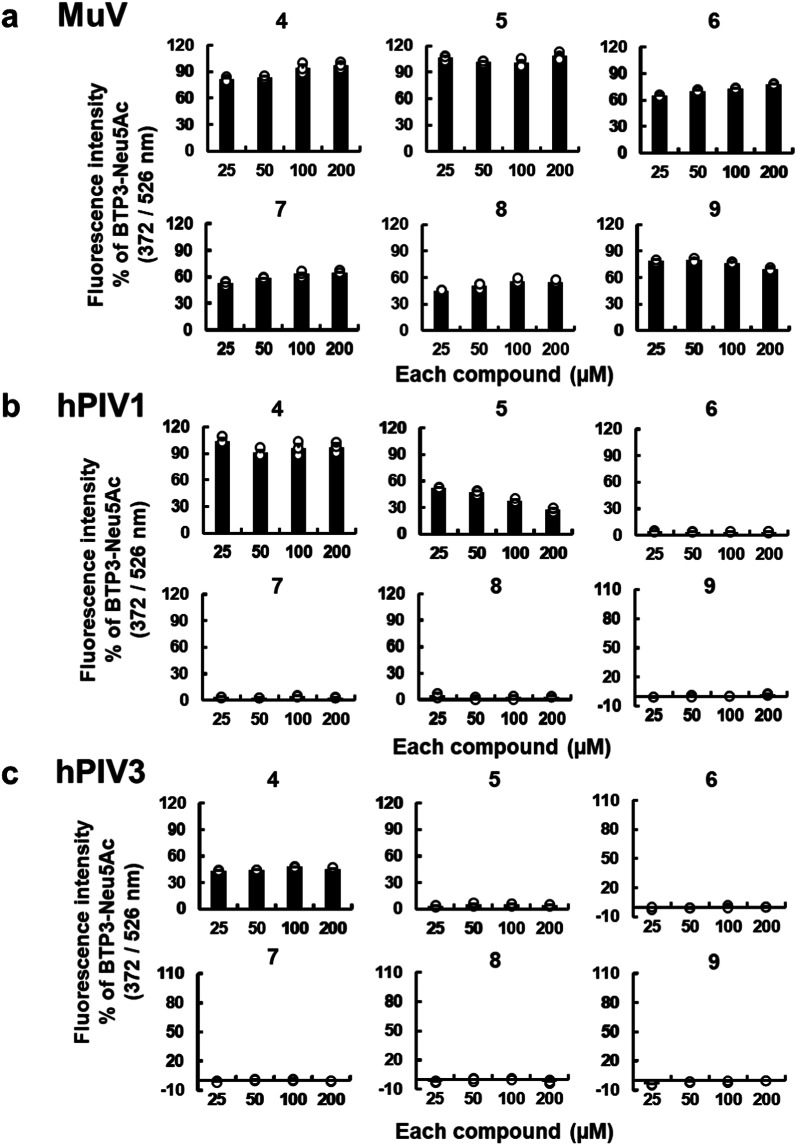

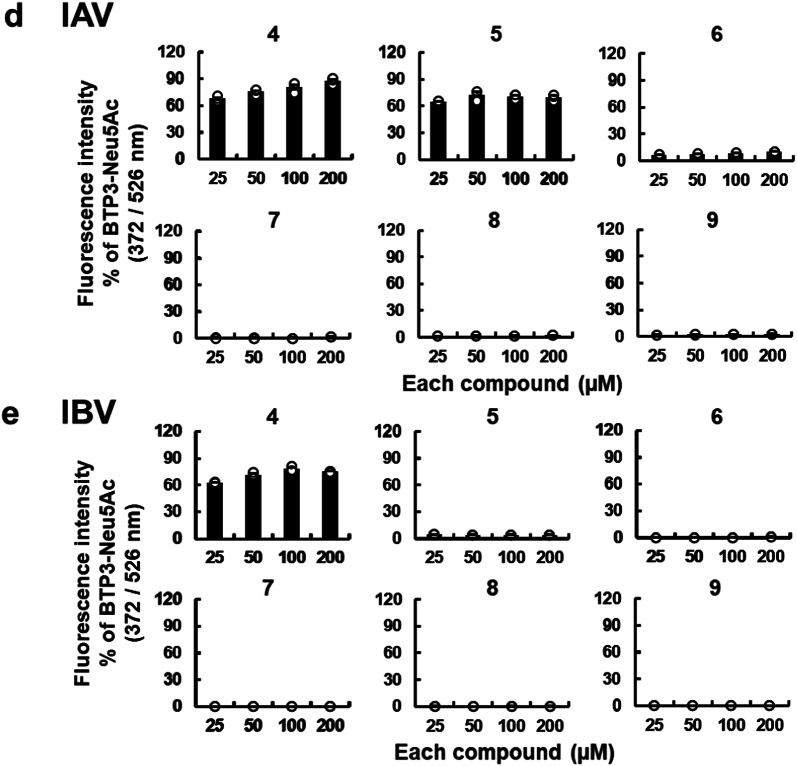
Concentration-dependent comparison of the sialidase activities of Compounds **4**–**9** in MuV-HN, hPIV1-HN, hPIV3-HN, IAV-NA, and IBV-NA. (**a**–**e**) 293T cells were transfected with expression vectors for MuV-HN (**a**), hPIV1-HN (**b**), hPIV3-HN (**c**), IAV-NA (PR8 strain) (**d**), and IBV-NA (**e**). Each set of HN– or NA–expressing cells was incubated with 25–200 µM of BTP3-Neu5Ac or each compound at 37 °C for 20 min. Sialidase activity is represented as fluorescence intensity relative to that obtained with BTP3-Neu5Ac (set as 100%).

The results for Compounds **4**–**6**, which contain linear *N*-acyl groups on 3–5 carbon atoms at the C5 position of the Neu5Ac moiety, suggested that the length of the *N*-acyl group plays a vital role in determining the specificity for MuV sialidase. To further explore this finding, we synthesized additional compounds—Compounds **7**, **8**, and **9**, bearing *N*-hexanoyl, *N*-heptanoyl, and *N*-octanoyl groups, respectively, at the C5 position (Fig. 5). The enzymatic reactivity of Compounds **4**–**9**, each bearing a linear *N*-acyl group of varying length, was analyzed by incubating viral sialidase–expressing cells with varying concentrations of each compound for 20 min at 37 °C. All the tested viral sialidases demonstrated concentration-dependent increases in fluorescence intensity in response to BTP3-Neu5Ac (Supplementary Fig. 4a–e). Compound **4** exhibited clear reactivity with all the tested sialidases. In contrast, Compounds **6**–**9** demonstrated distinct reactivity exclusively with MuV sialidase (Fig. 6a–e). Compounds **8** and **9**, which demonstrated high specificity for MuV sialidase, exhibited no detectable reactivity with commercially available bacterial sialidases from *Arthrobacter ureafaciens* (Supplementary Fig. 5a) and *Clostridium perfringens* (Supplementary Fig. 5b). These results indicated that longer linear *N*-acyl groups (5–8 carbon atoms) at the C5 position of the Neu5Ac moiety improve the specificity for MuV sialidase.

In contrast, Compound **4**, with a short three-carbon *N*-acyl group, reacted with all the tested viral sialidases. Compound **5** reacted with hPIV1 and IAV PR8 but exhibited no detectable reactivity with hPIV3 or IBV sialidase (Fig. 6b–e). For hPIV1 sialidase, the fluorescence intensity ratio of Compound **5** relative to BTP3-Neu5Ac decreased in a concentration-dependent manner, indicating that Compound **5** readily reached substrate saturation under study conditions (Fig. 6b). We also observed reactivity with Compound **5** for other IAV strains, including oseltamivir-resistant IAV S738 (Supplementary Fig. 6a) and the 2009 pandemic oseltamivir-sensitive A/Shizuoka/838/2009 H1N1 strain (IAV S838)^18^ (Supplementary Fig. 6b), but not for IAV M71 (Supplementary Fig. 6c). These IAV sialidases also demonstrated concentration-dependent fluorescence intensities when incubated with varying concentrations of BTP3-Neu5Ac (Supplementary Fig. 7a–c). The results obtained for IAV S738 suggested that the oseltamivir-resistant mutation exerted no significant impact on probe reactivity. Altogether, these findings suggested that linear *N*-acyl groups with more than three or four carbon atoms at the C5 position of the Neu5Ac moiety represent the minimal structural requirement for accessing the active sites of hPIV, IAV, and IBV sialidases as enzymatic substrates.

### BTP3-Neu5Ac derivatives containing long linear *N*-acyl groups at the C5 position of the Neu5Ac moiety allow for the specific visualization of MuV-infected cells

**Fig. 7:**
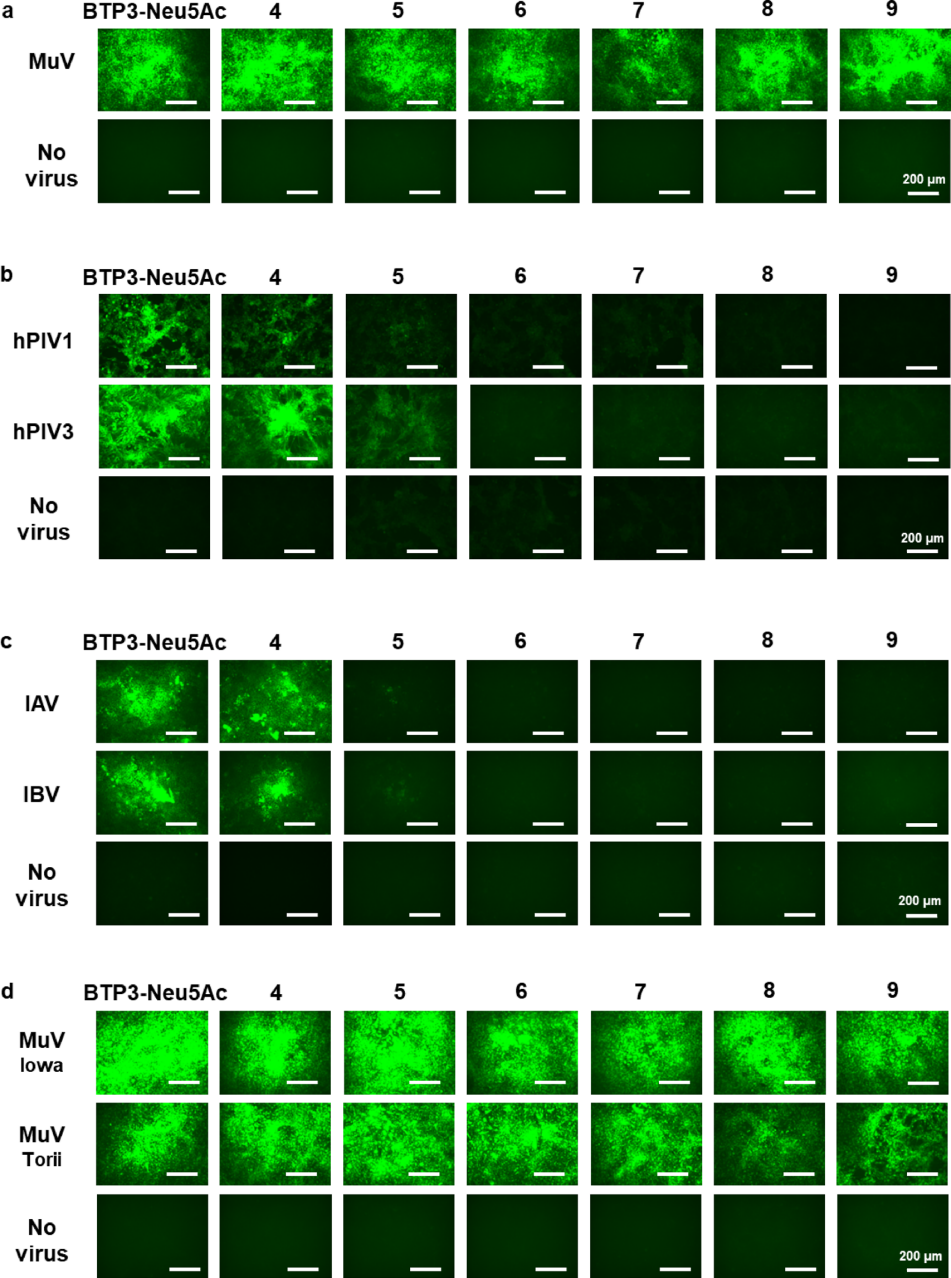
Fluorescence imaging of virus-infected cells using Compounds **4**–**9**. (**a**) Vero cells were infected with MuV (13V165E2 strain) and cultured for 48 h. (**b**) LLC-MK2 cells were infected with hPIV1 or hPIV3 and cultured for 48 h. (**c**) MDCK cells were infected with IAV (PR8 strain) or IBV and cultured for 24 h. (**d**) Vero cells were infected with two other MuV strains (Iowa and Torii strains) and cultured for 48 h. All infected cells were incubated with 200 µM of either BTP3-Neu5Ac or each compound at 37 °C for 20 min. Scale bars indicate 200 µm.

BTP3-Neu5Ac is characterized by its ability to enable fluorescence imaging of viral sialidase–expressing cells infected with MuV^14^, hPIV^15,28^, and IAV and IBV^16–18^. We explored whether BTP3-Neu5Ac derivatives containing linear *N*-acyl groups at the C5 position of the Neu5Ac moiety could also enable specific fluorescence visualization of MuV-infected cells. We infected African green monkey kidney Vero cells with MuV, rhesus monkey kidney epithelial LLC-MK2 cells with hPIV, and Madin–Darby canine kidney (MDCK) cells with IAV and IBV. After incubation for 48 h for MuV and hPIV or 24 h for IAV and IBV, the cells were incubated with 200 µM of BTP3-Neu5Ac or Compounds **4**–**9** for 20 min at 37 °C. We then obtained the fluorescence images of infected cells (Fig. 7) and of the entire wells (Supplementary Fig. 8). Clear fluorescence visualization of MuV-infected cells was achieved with Compounds **4**–**9** (Fig. 7a; Supplementary Fig. 8a), with only Compound **4** enabling clear fluorescence visualization of cells infected with hPIV1, hPIV3, IAV, or IBV (Fig. 7b,c; Supplementary Fig. 8b,c). Compound **5** exhibited weak fluorescence and Compounds **6**–**9** exhibited no fluorescence in the cells infected with hPIV1, hPIV3, IAV, or IBV. These results indicated that Compounds **6**–**9** were specific probes for MuV sialidase, enabling the selective fluorescence imaging of MuV-infected cells.

Three MuV strains were used in this study: one isolated in 2013 in Shizuoka city, Japan; one in 2006 in the United States (the Iowa strain); and one in 1969 in Japan (the Torii strain). Compounds **4**–**9** exhibited remarkable fluorescence visualization of cells infected with either the Iowa or Torii strain (Fig. 7d; Supplementary Fig. 8d). These findings suggested that Compounds **6**–**9** served as MuV-specific probes across multiple viral strains.

**Fig. 8:**
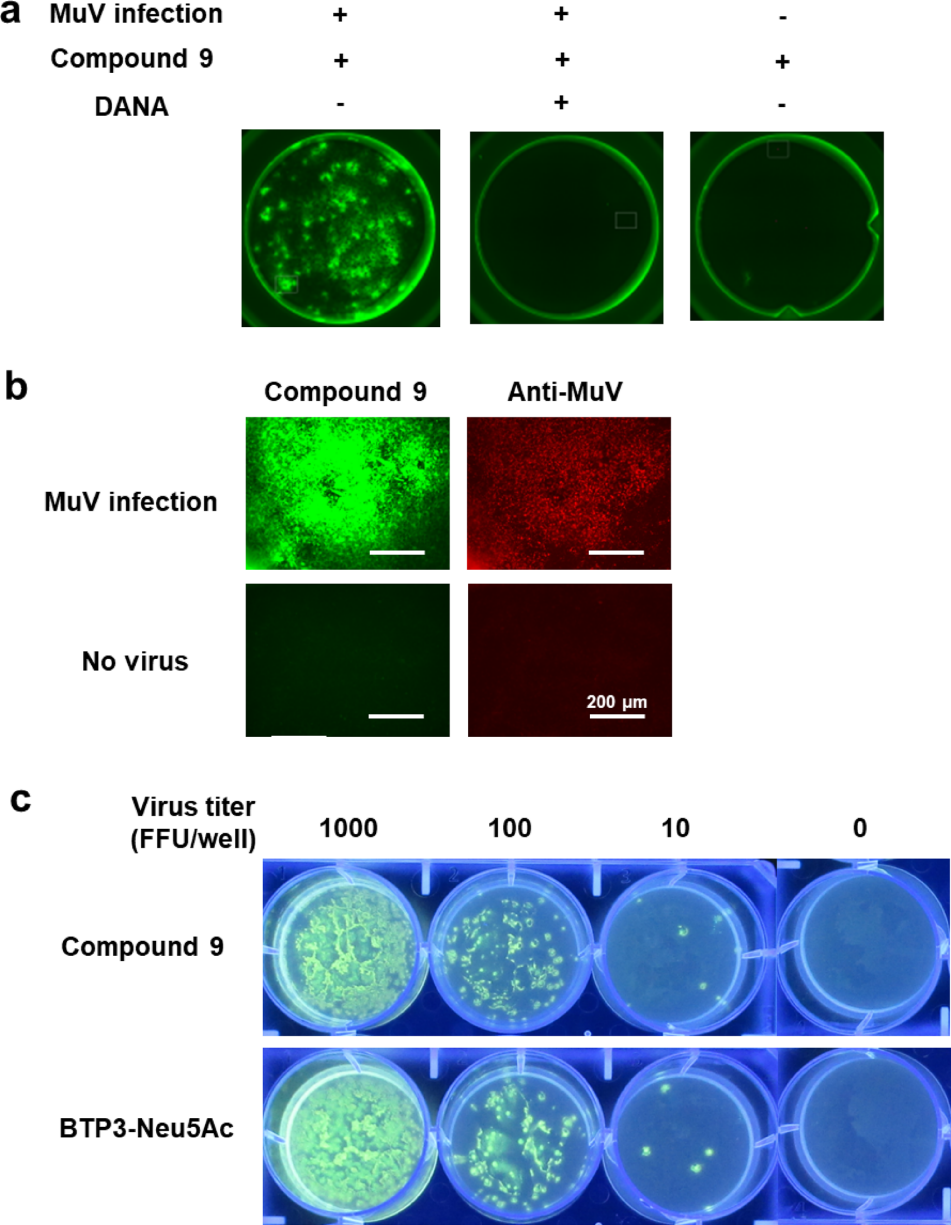
Fluorescence imaging of MuV-infected cells and viral focuses using Compound **9**. Vero cells were infected with MuV (13V165E2 strain) and cultured for 48 h. (**a**) The cells were incubated with 200 µM of Compound **9** in the presence or absence of 2 mM DANA at 37 °C for 20 min. Fluorescence images of the wells are shown. (**b**) The cells were incubated with 200 µM of Compound **9** at 37 °C for 20 min. After observing BTP3 fluorescence (green), the cells were treated with methanol for 1 min and subsequently immunostained with rabbit anti-MuV serum (red). Scale bars indicate 200 µm. **c** Vero cells were infected with MuV (13V165E2 strain) at the indicated FFU/well and overlaid with an agarose medium. After culturing at 37 °C for 72 h, the cells were incubated with 1 mM of either BTP3-Neu5Ac or Compound **9** at 37 °C for 4 h. Fluorescence images of viral focuses were captured under UV irradiation at 365 nm.

To determine whether the fluorescence visualization of MuV-infected cells depends on sialidase activity, we incubated MuV-infected cells with Compound **9** in the presence or absence of 2 mM 2,3-dehydro-2-deoxy-*N*-acetylneuraminic acid (DANA), a known competitive inhibitor of MuV sialidase^29,30^. DANA completely suppressed the fluorescence signal from MuV-infected cells (Fig. 8a), indicating that Compound **9** acts as a sialidase imaging probe through the detection of sialidase activity.

Sialidase imaging probes, such as BTP3-Neu5Ac, can be used for double-staining with antibodies^16,31^. We explored whether Compound **9** could also be employed for double-staining with an antibody. After the fluorescence visualization of MuV-infected cells with Compound **9**, BTP3 fluorescence was removed using methanol. Then, the MuV-infected cells were stained with an anti-MuV antibody. BTP3 fluorescence overlapped with the staining by the anti-MuV antibody (Fig. 8b), indicating that Compound **9** can be used for double-staining with antibodies.

BTP3-Neu5Ac is used for viral focus imaging, allowing for the measurement of viral infection titers and virus isolation^14,18^. Hence, we examined whether Compound **9** could also be used for viral focus imaging. We cultured MuV-infected cells in an agarose-containing medium for 72 h and then fluorescently visualized them after applying a solution of Compound **9** onto the agarose-containing medium and incubating it for 4 h at 37 °C. Similar to BTP3-Neu5Ac, Compound **9** successfully enabled viral focus imaging (Fig. 8c), indicating that Compound **9** can be used for MuV focus imaging.

## Discussion

In our previous study, we developed BTP3-Neu5Ac as an imaging probe for a wide range of sialidases from various sources, including viruses, bacteria, and mammals^16,19^. In the present study, we aimed to develop virus-specific sialidase imaging probes by chemically modifying the Neu5Ac moiety in BTP3-Neu5Ac. In MuV-HN, a hydrophobic cavity extends toward the C5 position of the Neu5Ac moiety within the binding site, suggesting that introducing hydrophobic groups at this C5 position could improve the specificity of BTP3-Neu5Ac-based probes for MuV sialidase. Consequently, the BTP3-Neu5Ac derivatives containing linear *N*-acyl groups with 5–8 carbons at the C5 position of the Neu5Ac moiety enabled the specific visualization of MuV sialidase. These findings demonstrated that chemical modifications to the Neu5Ac moiety can improve substrate specificity for targeted sialidases.

The BTP3-Neu5Ac derivatives containing bulky hydrophobic groups, such as the *N*-pivaloyl group (Compound **2**) and the *N*-benzoyl group (Compound **3**), at the C5 position, exhibited no reactivity with any viral sialidases. In contrast, the derivative containing an *N*-isobutanoyl group (Compound **1**) at the C5 position reacted with MuV, hPIV1, and IAV sialidases, although its reactivity was lower than that of BTP3-Neu5Ac. These findings suggested that a less bulky hydrophobic group is essential for reactivity with viral sialidases and that *N*-acetyl or *N*-isobutanoyl groups represent the minimal size required at the C5 position of the Neu5Ac moiety to enable recognition as a substrate for viral sialidases.

**Fig. 9:**
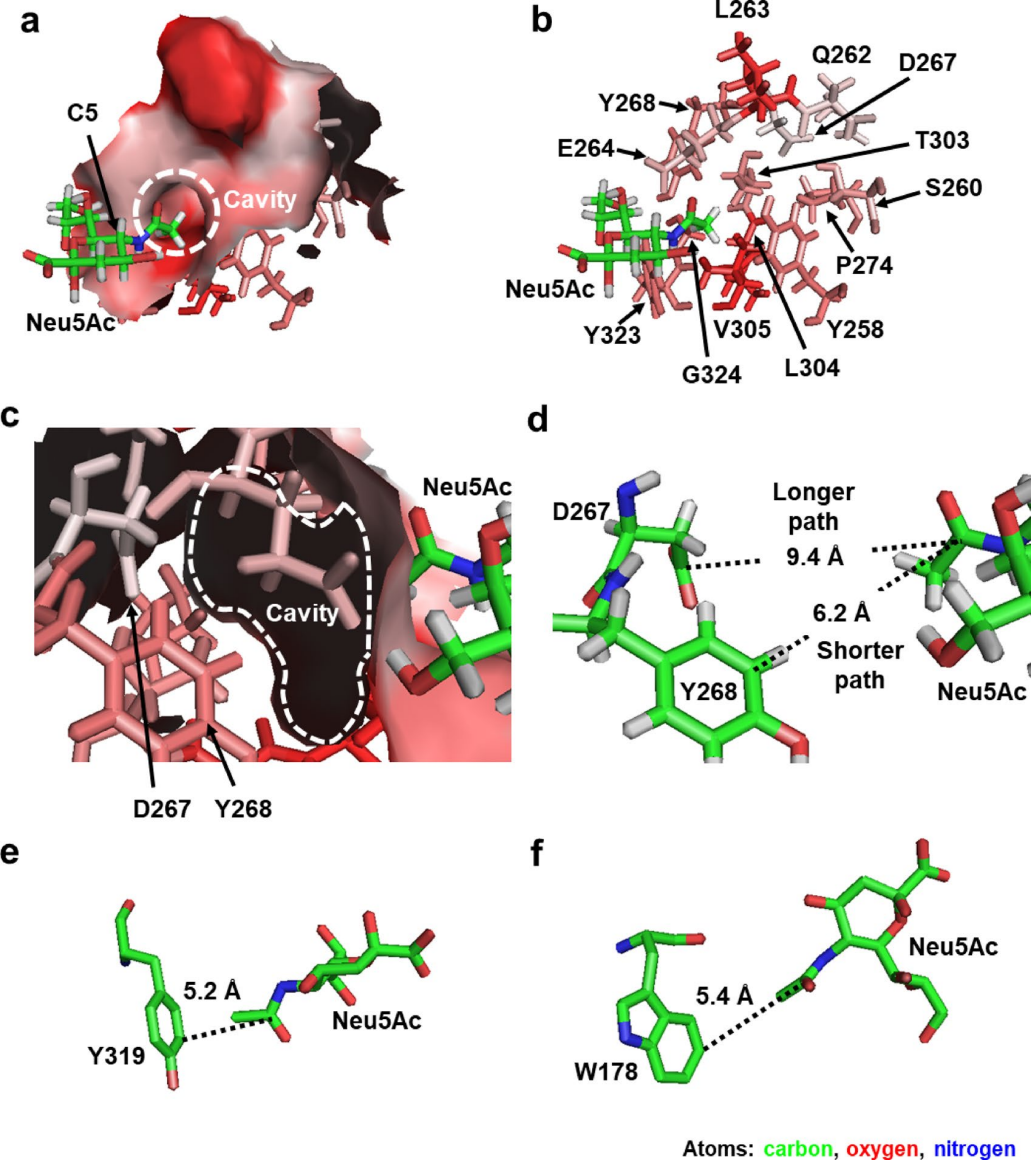
Amino acid residues constituting the hydrophobic cavity within the Neu5Ac-binding site in MuV-HN. Images are illustrated using the PyMOL software. Neu5Ac is depicted as a stick model, with carbon, oxygen, and nitrogen atoms represented in green, red, and blue, respectively. The hydrophobicity of amino acid residues is highlighted in red, and the hydrophobic cavity is outlined with a white dotted circle. (**a**) The amino acid residues constituting the hydrophobic cavity in MuV-HN (PDB ID 5B2D) are displayed as surface stick models. (**b**) The amino acid residues constituting the hydrophobic cavity are shown as a stick model, with the surface model from (**a**) hidden. (**c**) The amino acid residues constituting the hydrophobic cavity are displayed as both surface and stick models, with the view focused on the cavity within HN. (**d**) Distances are measured between the carbon atom of the carbonyl group in the side chain of aspartic acid at position 267 or the carbon atom at the C5 position of the phenolic group in the side chain of tyrosine at position 268 and the carbon atom of the carbonyl group at the C5 position in Neu5Ac. (**e**) In hPIV3-HN (PDB ID 1V3C), the distance is measured between the carbon atom at the C5 position of the phenolic group of tyrosine at position 319 and the carbon atom of the carbonyl group at the C5 position in Neu5Ac. (**f**) In IAV-NA (PDB ID 2BAT), the distance is measured between the carbon atom at the C5 position of the indole group of tryptophan at position 178 and the carbon atom of the carbonyl group at the C5 position in Neu5Ac.

The hydrophobic cavity in MuV-HN is composed of the amino acid residues depicted in Fig. 9a and b. For these residues, based on an alignment of 1635 MuV-HN protein sequences deposited in the GenBank database of the National Center for Biotechnology Information, we detected only two amino acid variations: aspartic acid at position 267 was substituted with asparagine in one strain (Massachusetts.USA/8.17 isolated in 2017), and proline at position 274 was substituted with leucine in another strain (San Sebastian.ESP/20.89.2 isolated in 1989). All other amino acid residues were completely conserved. This result indicated that the cavity within the Neu5Ac-binding site of MuV-HN is highly conserved across MuV strains. Consequently, Compounds **6**–**9** might specifically react with all MuV strains. In the crystallographic structure of MuV-HN^22^, lysine at position 242 directly interacts with the carbonyl oxygen atom of the *N*-acetyl group in Neu5Ac. This carbonyl group is probably essential for recognizing Neu5Ac as an enzymatic substrate by MuV-HN, and chemical modifications to this group could potentially disrupt the enzymatic activity of this sialidase. Conversely, the methyl group of the *N*-acetyl group in Neu5Ac does not appear to interact with any residues in MuV-HN, suggesting that it is not crucial for substrate recognition. This finding suggested that chemical modifications to the methyl group improve substrate specificity while maintaining the enzymatic reactivity of MuV-HN.

The analysis of the cavity from inside MuV-HN reveals two pathways leading to aspartic acid at position 267 or tyrosine at position 268 (Fig. 9c,d). The longer path (9.4 Å) spans between the carbon atom of the carbonyl group in the side chain of aspartic acid at position 267 and the carbon atom of the carbonyl group at the C5 position in Neu5Ac. The shorter path (6.2 Å) spans between the carbon atom at the C5 position of the phenolic group in the side chain of tyrosine at position 268 and the carbon atom of the carbonyl group at the C5 position in Neu5Ac (Fig. 9d). The *N*-acyl groups with 5–8 carbon atoms at the C5 position of Neu5Ac, which exhibited high specificity for MuV sialidase, correspond to an approximate length range of 6.0–10.5 Å, considering a distance of 1.5 Å per bond between two adjacent carbon atoms. However, in reality, the linear carbon chains adopt bent configurations. Therefore, the *N*-acyl groups with 5–8 carbon atoms might fit either pathway within the cavity. In hPIV3-HN, the carbon atom at the C5 position of the phenolic group of tyrosine at position 319 is 5.2 Å away from the carbon atom of the carbonyl group at the C5 position in Neu5Ac (Fig. 9e). Similarly, in IAV-NA, the carbon atom at the C5 position of the indole group of tryptophan at position 178 is 5.4 Å away from the carbon atom of the carbonyl group at the C5 position in Neu5Ac (Fig. 9f). These distances can accommodate *N*-acyl groups with three or four carbon atoms (approximately 3.0–4.5 Å long) but not those with 5–8 carbon atoms (approximately 6.0–10.5 Å long). This finding might underline the specific reactivity of Compounds **6**–**9** toward MuV sialidase.

Synthetic sialidase substrates based on Neu5Ac derivatives have been investigated for their specificity toward sialidases from viruses, bacteria, and mammals. 4,7-Di-*O*-methyl-Neu5Ac exhibited high specificity for sialidases from IAV and IBV, while showing no reactivity toward bacterial sialidases from *Arthrobacter ureafaciens*, *Clostridium perfringens*, *Streptococcus mitis*, and *Salmonella typhimurium*^20^. *N*-substituted derivatives at the C4 or C8 positions of Neu5Ac influenced reactivity toward sialidases from IAV, IBV, bacteria, and mammals. Neu5Ac derivatives substituted with NH_2_ or *N*-acetyl groups at the C8 position showed high reactivity toward sialidases from *Streptococcus pneumoniae* NanA. Among these, the C8 NH_2_-substituted derivative displayed higher reactivity to IAV N1 sialidase than to IAV N2 and IBV sialidases^32^. Derivatives substituted with N_3_ or *O*-acetyl groups at the C4 position showed high specificity for IAV and IBV sialidases^33^, suggesting that substitutions at the C4 position are important for specificity to IAV and IBV sialidases. Our present study demonstrates the novelty of Neu5Ac derivatives modified at the C5 position and their specificity for MuV sialidase. Chemical modifications of Neu5Ac can thus provide tools for detecting specific sialidases and contribute to the advancement of sialidase-related research, as well as to the diagnosis of sialidase-associated diseases, including infections with IAV, IBV, MuV, and hPIV.

In the present study, chemical modifications to Neu5Ac in BTP3-Neu5Ac could modulate the reactivity and specificity of viral sialidases. Therefore, we developed novel BTP3-Neu5Ac derivatives specifically targeting MuV sialidase. In a previous study, an analysis of the viral genes in oral fluids collected from 116 children (aged 2**–**12 years) clinically diagnosed with mumps between December 2014 and February 2015 in England revealed that the MuV gene was detected in only nine patients, whereas the IAV (H3N2) gene was identified in 16 patients^26^. This finding emphasized the challenge faced during the accurate diagnosis of MuV infections. In particular, human studies have reported cases of co-infections with MuV, hPIV, and IAV^8,25,26^, further complicating the precise identification and diagnosis of MuV infections. Despite these challenges, no simple and rapid detection system currently exists for MuV infection. However, a virus detection system employing BTP3-Neu5Ac and a molecular cutoff ultrafiltration membrane, developed in our previous study, demonstrated higher sensitivity for detecting IAV compared to commercial diagnostic kits using antiviral nucleoprotein antibodies^17^. Furthermore, the ability of BTP3-Neu5Ac derivatives to achieve easy and rapid fluorescence imaging of virus-infected cells might prove instrumental for epidemiological investigations in various environmental and hygiene conditions, which rely on infected cells for the propagation and isolation of the virus. In the future, Compounds **6**–**9** and their derivatives might serve as valuable tools for diagnosing and conducting epidemiological investigations of MuV infections. We are further exploring the chemical modification of Neu5Ac in BTP3-Neu5Ac to develop novel derivatives with improved reactivity and specificity for hPIV, IAV, and IBV.

## Methods

### Cells, viruses, and chemicals

Cells were obtained from the American Type Culture Collection (ATCC). MDCK cells (ATCC-CCL-34), rhesus monkey kidney epithelial LLC-MK2 cells (ATCC-CCL-7), and African green monkey kidney Vero cells (ATCC-CCL-81) were maintained in minimum essential medium (MEM; 41500034, Thermo Fisher Scientific, Waltham, MA, USA) supplemented with 5% fetal bovine serum (FBS; S1600-500, BioWest, Bradenton, FL, USA) at 37 °C under 5% CO_2_. Human embryonic kidney 293T cells (ATCC-CRL-3216) were maintained in Dulbecco’s modified Eagle medium (DMEM; 12800017, Thermo Fisher Scientific, Waltham, MA, USA) supplemented with 10% FBS at 37 °C under 5% CO_2_. MuV Shizuoka 13V165E2 strain^14,30,34^ was propagated in Vero cells using 1 µg/mL of TPCK-trypsin (LS003740, Worthington Biochemical Corporation, Lakewood, NJ, USA). MuV Torii (ATCC-VR-1880) and Iowa. US/2006 (ATCC-VR-1899) strains were obtained from ATCC and propagated in Vero cells using 1 µg/mL of TPCK-trypsin. IAV A/PR/8/1934 H1N1 and IBV B/Lee/1940 strains^17^ were propagated in MDCK cells using 1 µg/mL of TPCK-trypsin. hPIV1 C35 and hPIV3 C243 stains^35^ were propagated in LLC-MK2 cells using 1 µg/mL of TPCK-trypsin. DANA (A2205) and BTP3-Neu5Ac (B6529) were purchased from Tokyo Chemical Industry Co., Ltd, Tokyo, Japan. DANA was stored at − 30 °C as stock solutions (100 mM) prepared in sterile distilled water. BTP3-Neu5Ac was stored at − 30 °C as stock solutions (100 mM) prepared in dimethyl sulfoxide (DMSO; D2650, SIGMA-ALDRICH, Tokyo, Japan). We synthesized all BTP3-Neu5Ac derivatives (see Supplementary information for synthesis details and chemical data of Compounds **1**–**9**) and stored them at − 30 °C as stock solutions (100 mM) prepared in DMSO.

### Focus-forming assay

Vero cells and LLC-MK2 cells were used for MuV and hPIV infections, respectively, and MDCK cells were used for IAV and IBV infections. The cells were seeded at a density of 4 × 10^5^ cells/well (2.5 mL/well) in a 6-well plate and cultured overnight at 37 °C under 5% CO_2_. A confluent monolayer of cells was washed with 1 mL/well of phosphate-buffered saline (PBS, containing 131 mM NaCl, 14 mM Na_2_HPO_4_, 1.5 mM KH_2_PO_4_, and 2.7 mM KCl; pH 7.2) and inoculated with tenfold serially diluted virus solution (1 mL/well) in serum-free medium (SFM; 12300067, Hybridoma-SFM Complete DPM, Thermo Fisher Scientific, Waltham, MA, USA) at 37 °C under 5% CO_2_ for 1 h. After washing with 1 mL/well of PBS, the cells were cultured in 4 mL/well of SFM containing 1 µg/mL of TPCK-trypsin and 0.8% agarose (Bacto Agar, 214010, Japan Becton Dickinson Company, Ltd., Tokyo, Japan) at 37 °C under 5% CO_2_ for 48 h for MuV, IAV, and IBV or for 96 h for hPIV1 and hPIV3, after inverting the plate. Viral focuses (populations of virus-infected cells) were fluorescently visualized using BTP3-Neu5Ac^14–16^. The supernatant obtained after the centrifugation (4 °C, 10,000×*g*, 10 min) of 1 mM BTP3-Neu5Ac in SFM for MuV, IAV, and IBV or in SFM adjusted to pH 4.5 (with HCl) for hPIV1 and hPIV3 was used as the BTP3-Neu5Ac solution to remove excess water-insoluble fluorescent BTP3 before use. Next, 200 µL/well of BTP3-Neu5Ac solution was added to the overlaid agarose medium on the plate and incubated at 37 °C under 5% CO_2_ for 4 h for MuV, IAV, and IBV or 6 h for hPIV1 and hPIV3. Viral focuses were fluorescently visualized under UV irradiation at 365 nm using a GUV-720 transilluminator (Natural Immunity, Tokyo, Japan). The infection titer, measured as focus-forming units (FFUs), was determined by counting the number of focuses.

To analyze the applicability of Compound **9** for the focus-forming assay, a confluent monolayer of Vero cells in a 6-well plate was washed with 1 mL/well of PBS and inoculated with the MuV 13V165E2 strain (1000, 100, and 10 FFU/well) in SFM (1 mL/well) at 37 °C under 5% CO_2_ for 1 h. After washing with 1 mL/well of PBS, the cells were cultured in 4 mL/well of SFM containing 1 µg/mL of TPCK-trypsin and 0.8% agarose at 37 °C under 5% CO_2_ for 72 h, after inverting the plate. Viral focuses were fluorescently visualized using either BTP3-Neu5Ac or Compound **9**. The supernatant obtained after the centrifugation (4 °C, 10,000×*g*, 10 min) of 1 mM BTP3-Neu5Ac or 1 mM Compound **9** in SFM (200 µL/well) was added to the overlaid agarose medium on the plate, followed by incubation at 37 °C under 5% CO_2_ for 4 h. Viral focuses were fluorescently visualized under UV irradiation at 365 nm using a GUV-720 transilluminator.

### Sialidase assay

293T cells were seeded at a density of 5 × 10^5^ cells/well (2.5 mL/well) in a 6-well plate and cultured overnight at 37 °C under 5% CO_2_. A 70–90% confluent monolayer of 293T cells was transfected with the expression plasmid vector pCAGGS/MCS containing the HN gene of MuV 13V165E2, hPIV1 C35, and hPIV3 C243 strains and the NA gene of IAV A/PR/8/1934 H1N1 and IBV B/Lee/1940 strains using the transfection reagent TransIT-293 (MIR2700, Mirus, Madison, WI, USA) according to the manufacturer’s instructions. pCAGGS/MCS was used as a negative control for the empty vector. At 72 h post-transfection at 37 °C under 5% CO_2_, the culture medium was removed, and the HN– or NA–expressing cells were collected by suspending in PBS (1 mL/well). The cells were then centrifuged (4 °C, 1000×*g*, 10 min), and the supernatant was replaced with 10 mM acetate buffer (1 mL/well) at pH 4.5 for MuV and hPIV or at pH 6.0 for IAV to achieve the optimal pH for each viral sialidase. BTP3-Neu5Ac and its derivatives were serially diluted two-fold to concentrations ranging from 0.125 mM to 1 mM in 10 mM acetate buffer at pH 4.5 for MuV and hPIV or at pH 6.0 for IAV and IBV (final concentrations: 25–200 µM). The supernatant of compound solutions was collected by centrifugation (4 °C, 10,000×*g*, 10 min). The HN– or NA–expressing cells in 10 mM acetate buffer (40 µL/well) were mixed with each compound (10 µL/well) in a 96-well black plate on ice. Sialidase reaction was performed by incubating the plate at 37 °C for 20 min at pH 4.5 for MuV and hPIV or pH 6.0 for IAV and IBV. The reaction was terminated by adding 50 µL/well of 100 mM sodium carbonate buffer (pH 10.7). BTP3 fluorescence was measured using an Infinite M200 microplate reader (TECAN Group Ltd., Männedorf, Switzerland) with excitation/emission wavelengths of 372/526 nm. Fluorescence intensity values were normalized by subtracting the fluorescence obtained through the negative control (empty vector pCAGGS/MCS). The experiments were independently repeated three times (n = 3). The graphs presented the mean ± standard error of the mean.

### Fluorescence visualization of virus-infected cells

Cells were seeded at a density of 2 × 10^3^ cells/well (100 µL/well) in a 96-well plate and cultured overnight at 37 °C under 5% CO_2_. The cells were washed with PBS (50 µL/well) and infected with the respective virus (100 FFU/well) in SFM (50 µL/well) at 37 °C under 5% CO_2_ for 60 min. After washing with PBS (50 µL/well), the cells were incubated in SFM (50 µL/well) at 37 °C under 5% CO_2_ for 24 h for IAV and IBV or 48 h for MuV and hPIV. Next, 200 µM solutions of BTP3-Neu5Ac and its derivative compounds were prepared in SFM for MuV, IAV, and IBV or in SFM adjusted to pH 4.5 (with HCl) for hPIV. The supernatant of compound solutions was collected by centrifugation (4 °C, 10,000×*g*, 10 min). Then, the cells were washed with PBS (50 µL/well) and incubated with 200 µM of each compound (50 µL/well) at 37 °C under 5% CO_2_ for 20 min. After washing with PBS (50 µL/well) and adding SFM (50 µL/well), the cells were observed under a BZ-X700 fluorescence microscope (KEYENCE, Osaka, Japan) equipped with a fluorescent filter (excitation wavelength of 360/40 nm, emission wavelength of 525/50 nm, and dichroic mirror of 400 nm). Fluorescent cells in the microplate wells were observed using a 4× objective lens (Supplementary Fig. 8). Representative fluorescent cells (highlighted by squares in Supplementary Fig. 8) were further observed using a 20× objective lens and are depicted in Fig. 7.

To confirm that BTP3 fluorescence was dependent on viral sialidase activity, Vero cells were seeded at 2 × 10^3^ cells/well in 10-well glass slide CELLview (543979, Greiner Bio-One, Oberösterreich, Austria) and cultured overnight at 37 °C under 5% CO_2_. Then, the cells were washed with PBS (50 µL/well) and infected with MuV Shizuoka 13V165E2 strain (100 FFU/well) in SFM (50 µL/well) at 37 °C under 5% CO_2_ for 60 min. After washing with PBS (50 µL/well), the cells were incubated in SFM (50 µL/well) at 37 °C under 5% CO_2_ for 48 h. MuV-infected cells were also incubated with 200 µM of Compound **9** (50 µL/well) in the presence of 2 mM DANA, a sialidase inhibitor of MuV^29,30^. Fluorescent cells in the wells were observed using a 4× objective lens.

To confirm that BTP3 fluorescence was observed in MuV-infected cells, immunostaining was performed on MuV-infected cells in a 10-well glass slide CELLview using rabbit anti-MuV serum prepared by us previously^34^. After observing BTP3 fluorescence, the cells were treated with methanol (50 µL/well) at room temperature for 1 min to remove BTP3. Then, the cells were washed with PBS (50 µL/well) and incubated with rabbit anti-MuV serum and Alexa Fluor Plus 555–labeled goat anti-rabbit IgG antibody (A32732, Thermo Fisher Scientific, Waltham, MA, USA) at room temperature for 1 h. After washing with PBS (50 µL/well) and adding PBS (50 µL/well), the cells were observed under a BZ-X700 fluorescence microscope equipped with a TRITC fluorescent filter (OP-87764, excitation wavelength of 545/25 nm, emission wavelength of 605/70 nm, and dichroic mirror of 565 nm) using a 20× objective lens.

## Supplementary Information

Below is the link to the electronic supplementary material.

Supplementary Material 1

## Data Availability

The data that support the findings of this study are available from the corresponding author, T. T., upon reasonable request.
