# Supplementary material for: Development of mumps virus-specific sialidase imaging probes through chemical modifications of sialic acid
Source: Sci Rep. 2025 Nov 25;15:42005. doi: 10.1038/s41598-025-26190-y (PMC12647717; doi:10.1038/s41598-025-26190-y)
Supplement: Supplementary file 1 — Supplementary Material 1 [file 41598_2025_26190_MOESM1_ESM.pdf]

Supplementary Information

Development of Mumps Virus-Specific Sialidase Imaging Probes Through Chemical Modifications of Sialic Acid

Yutaka Narimichi[1,†], Tadanobu Takahashi[1,†,*], Yuuki Kurebayashi[1,†], Tadamune Otsubo[2], Kiyoshi Ikeda[2], Yu Saito[1], Akira Minami[3], Hideyuki Takeuchi[1,*]

[1]Department of Biochemistry, School of Pharmaceutical Sciences, University of Shizuoka, 52-1 Yada, Suruga-ku, Shizuoka-shi, Shizuoka, 422-8526, Japan

[2]Department of Organic Chemistry, School of Pharmaceutical Sciences, Hiroshima International University, 5-1-1 Hirokoshinkai, Kure-shi, Hiroshima 737-0112, Japan

[3]Department of Functional Morphology, Faculty of Pharmacy, Juntendo University, 6-8-1 Hinode, Urayasu-shi, Chiba, 279-0013, Japan

[†]They contributed equally as first authors.

*Corresponding author: Email address: takahasi@u-shizuoka-ken.ac.jp (T. Takahashi), htakeuchi@u-shizuoka-ken.ac.jp (H. Takeuchi)

Methods

Sialidase assay for Supplementary Figures

293T cells were seeded at a density of 5 × 10^5 cells/well (2.5 mL/well) in a 6-well plate and cultured overnight at 37°C under 5% CO_2. A 70%–90% confluent monolayer of 293T cells was transfected with the expression plasmid vector pCAGGS/MCS containing the HN gene of MuV (13V165E2 strain), hPIV1 (C35 strain), and hPIV3 (C243 strain) and the NA gene of IAVs (A/PR/8/1934, IAV PR8; A/Shizuoka/738/2008 H1N1, IAV S738; A/Shizuoka/838/2009 H1N1, IAV S838; and A/Memphis/1/1971 H3N2, IAV M71 strains) and IBV (B/Lee/1940 strain) using the transfection reagent TransIT-293 (MIR2700, Mirus, Madison, WI, USA) according to the manufacturer's instructions. pCAGGS/MCS was used as a negative control for the empty vector. At 72 h post-transfection at 37°C under 5% CO_2, the culture medium was removed, and the HN– or NA–expressing cells were collected by suspending them in PBS (1 mL/well). The suspension was centrifuged (4°C, 1000 ×*g*, 10 min), and the supernatant was replaced with 10 mM acetate buffer (1 mL/well) at pH 4.5 for MuV and hPIV or at pH 6.0 for IAV, to achieve the optimal pH for each viral sialidase. BTP3-Neu5Ac and its derivatives were serially diluted two-fold to concentrations ranging from 0.125 mM to 1 mM in 10 mM acetate buffer at pH 4.5 for MuV and hPIV or at pH 6.0 for IAV and IBV (final concentrations: 25–200 μM). The supernatant of compound solutions was collected by centrifugation (4°C, 10,000 ×*g*, 10 min).

The HN– or NA–expressing cells in 10 mM acetate buffer (40 µL/well) were mixed with each compound (10 µL/well) in a 96-well black plate on ice. Sialidase reaction was performed by incubating the cells at 37°C for 20 min at pH 4.5 for MuV and hPIV or at pH 6.0 for IAV and IBV. The reaction was terminated by adding 50 µL/well of 100 mM sodium carbonate buffer (pH 10.7). BTP3 fluorescence was measured using an Infinite M200 microplate reader (TECAN Group Ltd., Männedorf, Switzerland) with excitation/emission wavelengths of 372/526 nm. The experiments were independently repeated three times (n = 3).

To evaluate the reactivity of Compounds **8** and **9** with commercial bacterial sialidases, 10 mU/mL of *Arthrobacter ureafaciens* sialidase (AUSA; 10269611001, Roche Diagnostics, Basel, Switzerland) or *Clostridium perfringens sialidase* (CPSA; 11585886001, Roche Diagnostics, Basel, Switzerland) (40 µL/well) was incubated with the indicated concentrations of BTP3-Neu5Ac, Compound **8**, or Compound **9** (10 µL/well) in 10 mM acetate buffer (pH 4.5) at 37°C for 20 min. The reaction was terminated by adding 50 µL/well of 100 mM sodium carbonate buffer (pH 10.7). BTP3 fluorescence was measured using an Infinite M200 microplate reader with excitation/emission wavelengths of 372/526 nm. The experiments were independently repeated three times (n = 3). The graphs presented the mean ± standard error of the mean.

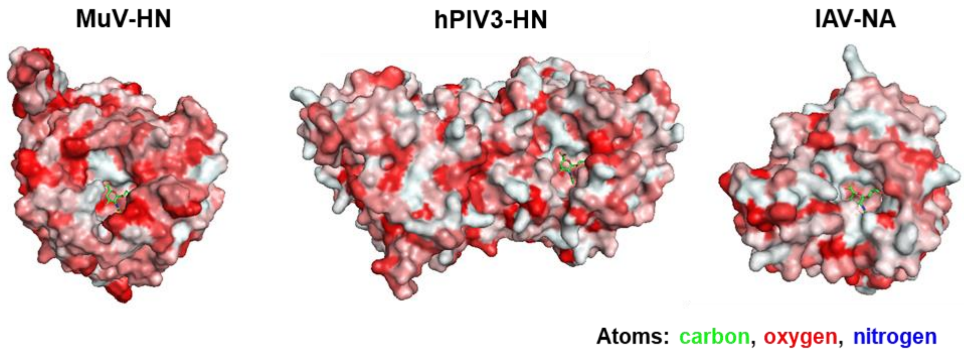

Supplementary Figure 1: Three-dimensional structures of MuV-HN, hPIV3-HN, and IAV-NA. The structural data of MuV-HN monomer (PDB ID 5B2D), hPIV3-HN dimer (PDB ID 1V3C), and IAV-NA monomer (PDB ID 2BAT) were obtained from the Protein Data Bank and displayed using the PyMOL Molecular Graphics System version 1. 1r. 1 (Delano Scientific LLC.). HN and NA are displayed as surface models, with the hydrophobic regions highlighted in red. Neu5Ac is depicted as a stick model, with carbon, oxygen, and nitrogen atoms represented in green, red, and blue, respectively.

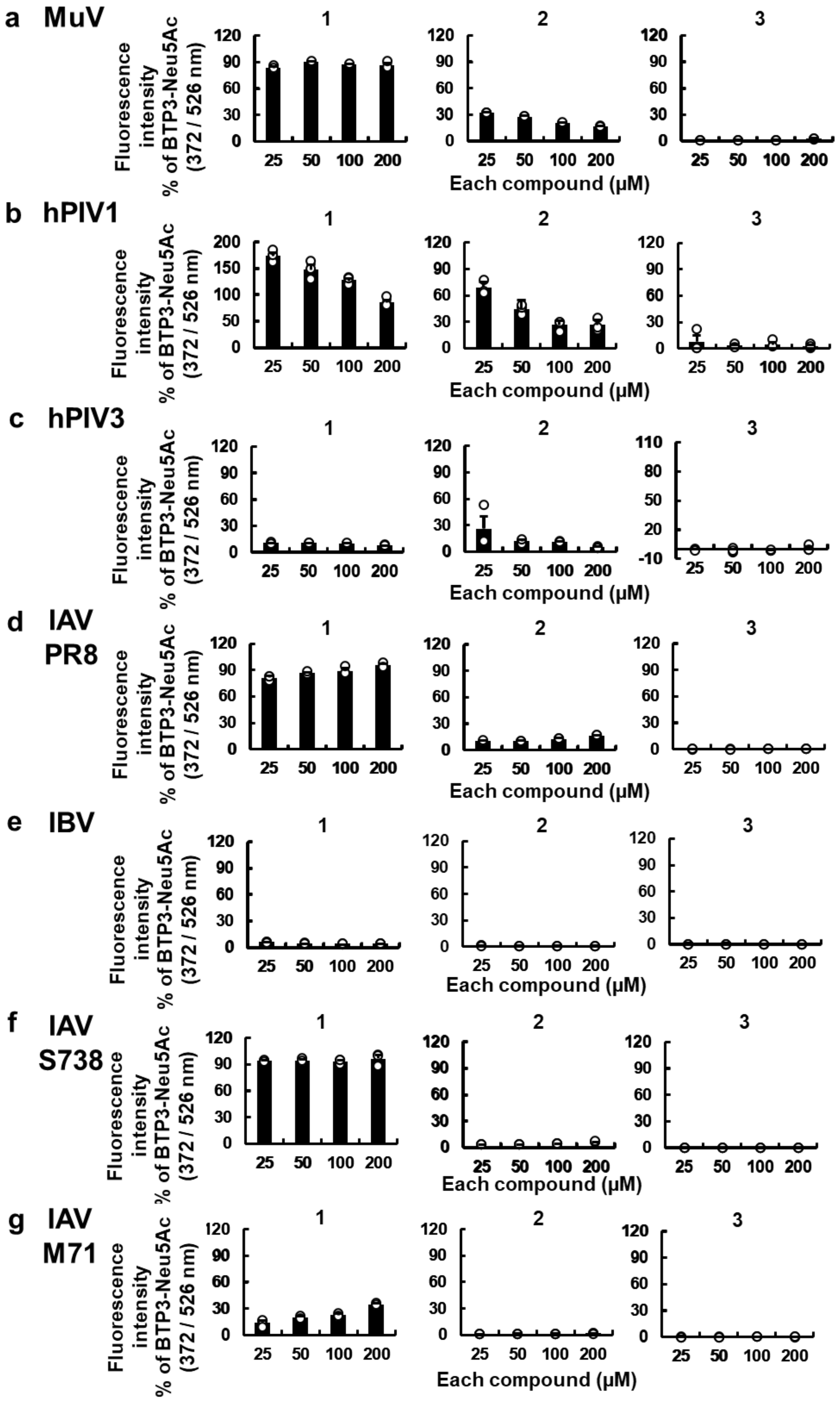

a MuV
b hPIV1
c hPIV3
d IAV PR8
e IBV
f IAV S738
g IAV M71
1
2
3
Fluorescence intensity % of BTP3-Neu5Ac (372 / 526 nm)
Each compound (μM)
25 50 100 200

Supplementary Figure 2: Concentration-dependent comparison of the sialidase activities of Compounds **1–3** relative to BTP3-Neu5Ac in MuV-HN, hPIV1-HN, hPIV3-HN, IAV-NA, and IBV-NA. **a–g** 293T cells were transfected with expression vectors for MuV-HN (**a**), hPIV1-HN (**b**), hPIV3-HN (**c**), IAV-N1NA (IAV PR8 strain) (**d**), IBV-NA (**e**), oseltamivir-resistant IAV-N1NA (IAV S738 strain) (**f**), and IAV-N2NA (IAV M71 strain) (**g**). Each set of HN- or NA-expressing cells was incubated with 25–200 μM of either BTP3-Neu5Ac or each compound at 37°C for 20 min. Sialidase activity is expressed as fluorescence intensity relative to that obtained with BTP3-Neu5Ac (set as 100%).

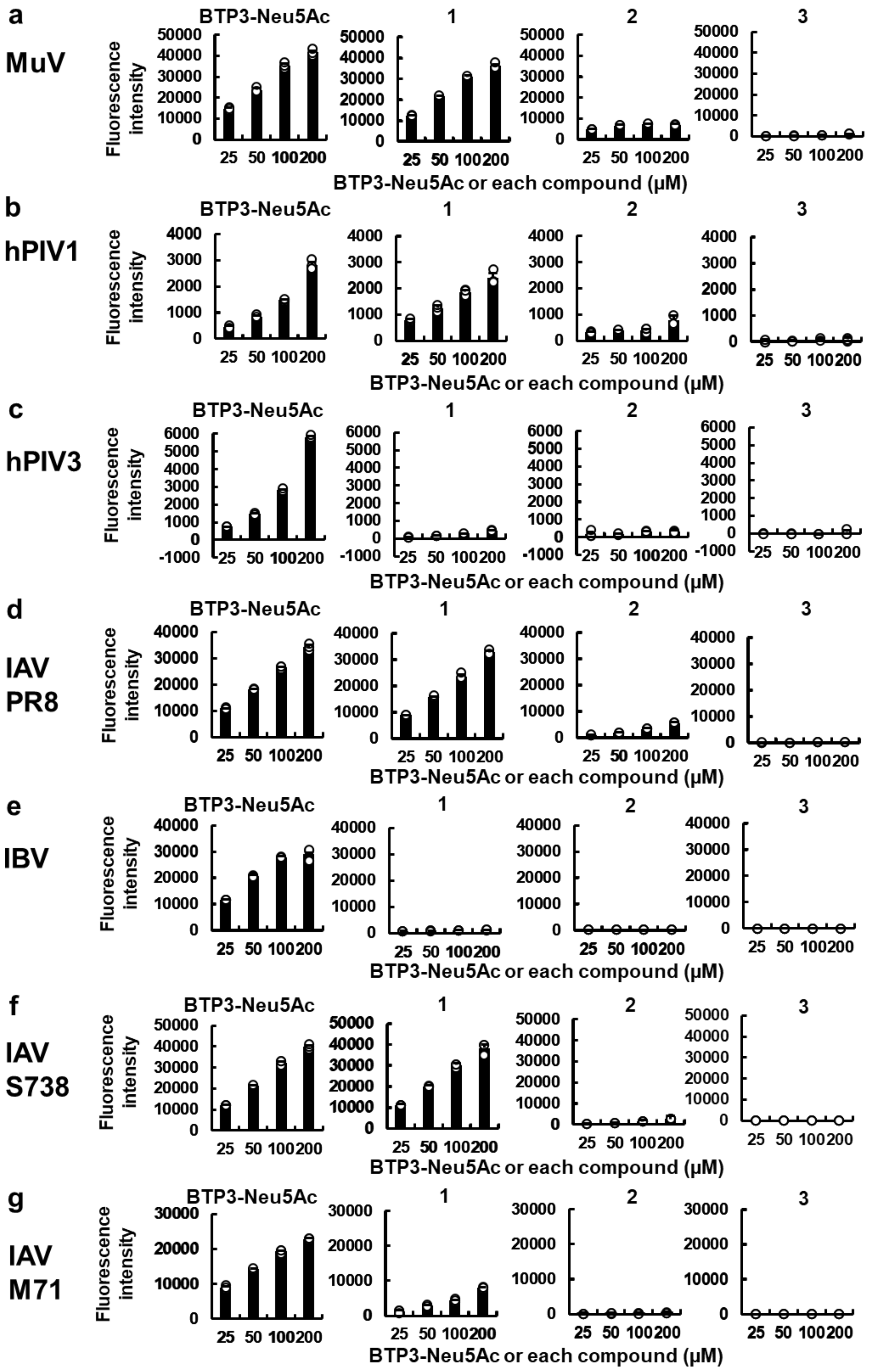
a
MuV
b
hPIV1
c
hPIV3
d
IAV PR8
e
IBV
f
IAV S738
g
IAV M71
BTP3-Neu5Ac
1
2
3
Fluorescence intensity
25 50 100 200
BTP3-Neu5Ac or each compound (μM)

Supplementary Figure 3: Concentration-dependent comparison of the sialidase activities of Compounds **1**–**3** in MuV-HN, hPIV1-HN, hPIV3-HN, IAV-NA, and IBV-NA. **a**–**g** 293T cells were transfected with expression vectors for MuV-HN (**a**), hPIV1-HN (**b**), hPIV3-HN (**c**), IAV-N1NA (IAV PR8 strain) (**d**), IBV-NA (**e**), oseltamivir-resistant IAV-N1NA (IAV S738 strain) (**f**), and IAV-N2NA (IAV M71 strain) (**g**). Each set of HN- or NA-expressing cells was incubated with 25–200 μM of either BTP3-Neu5Ac or each compound at 37°C for 20 min. Sialidase activity is expressed as the fluorescence intensity of BTP3.

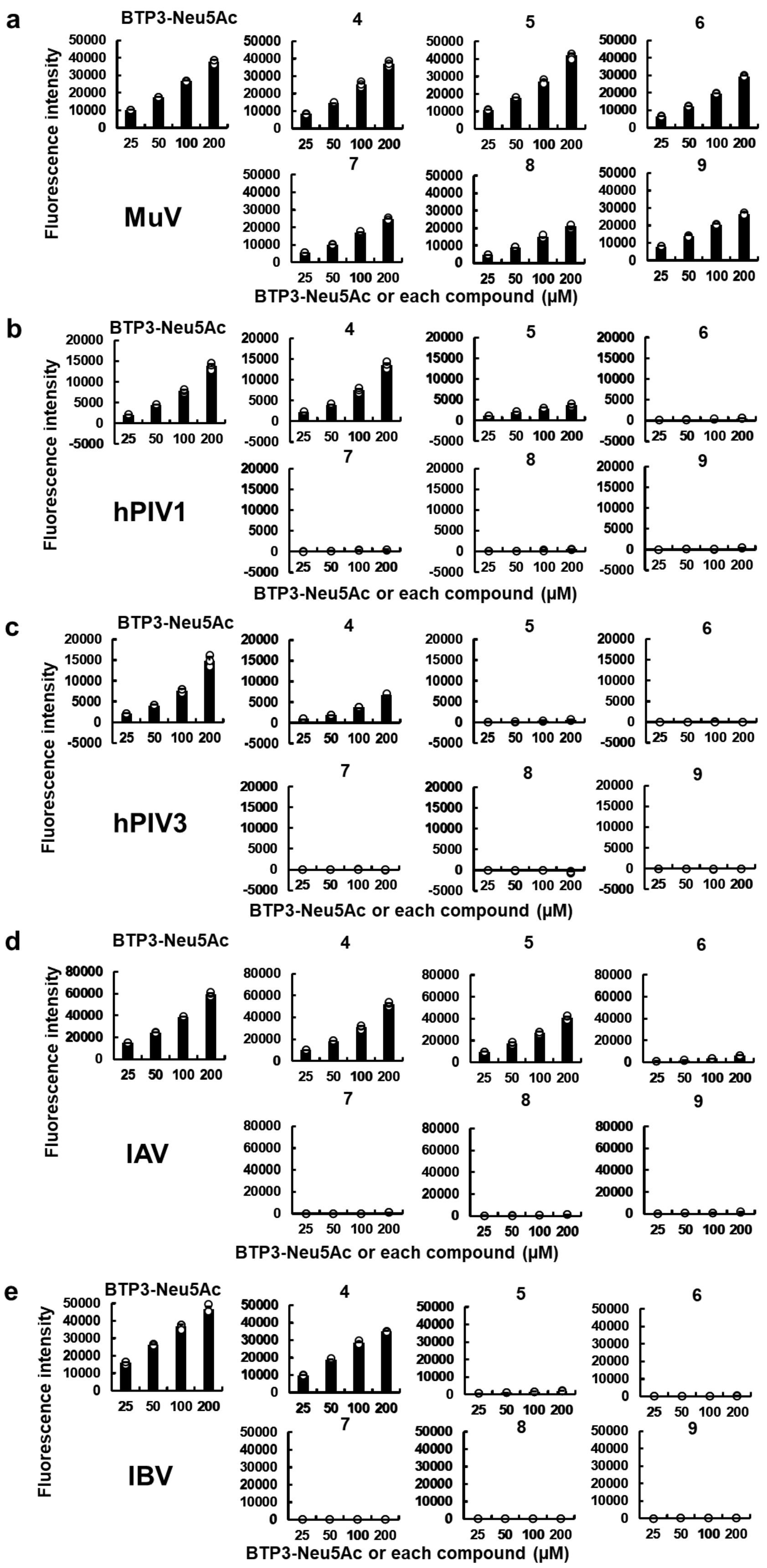
a
b
c
d
e
BTP3-Neu5Ac
4
5
6
7
8
9
MuV
hPIV1
hPIV3
IAV
IBV
Fluorescence intensity
25
50
100
200
BTP3-Neu5Ac or each compound (μM)

Supplementary Figure 4: Concentration-dependent comparison of the sialidase activities of Compounds **4–9** in MuV-HN, hPIV1-HN, hPIV3-HN, IAV-NA, and IBV-NA. **a–e** 293T cells were transfected with expression vectors for MuV-HN (**a**), hPIV1-HN (**b**), hPIV3-HN (**c**), IAV-N1NA (IAV PR8 strain) (**d**), and IBV-NA (**e**). Each set of HN- or NA-expressing cells was incubated with 25–200 μM of either BTP3-Neu5Ac or each compound at 37°C for 20 min. Sialidase activity is represented as the fluorescence intensity of BTP3.

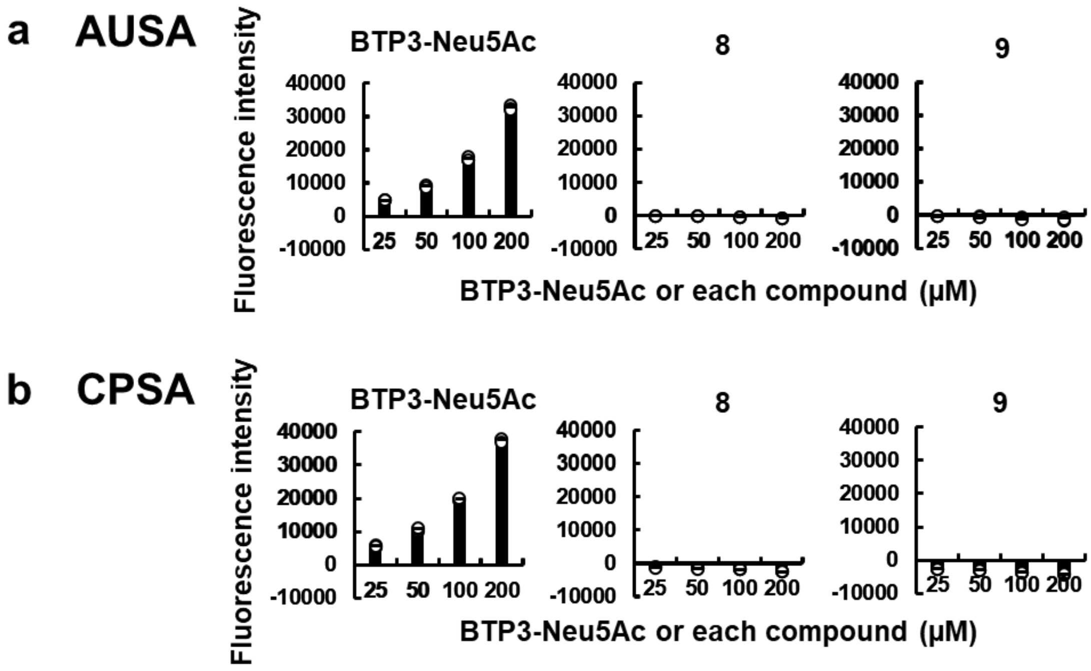

Supplementary Figure 5: Comparison of Compounds **8** and **9** for the activity of bacterial sialidases in a concentration-dependent manner. **a–b** *Arthrobacter ureafaciens* sialidase (AUSA) (**a**) or *Clostridium perfringens* sialidase (CPSA) (**b**) was incubated with 25–200 µM of either BTP3-Neu5Ac or each compound at 37°C for 20 min. Sialidase activity is expressed as the fluorescence intensity of BTP3.

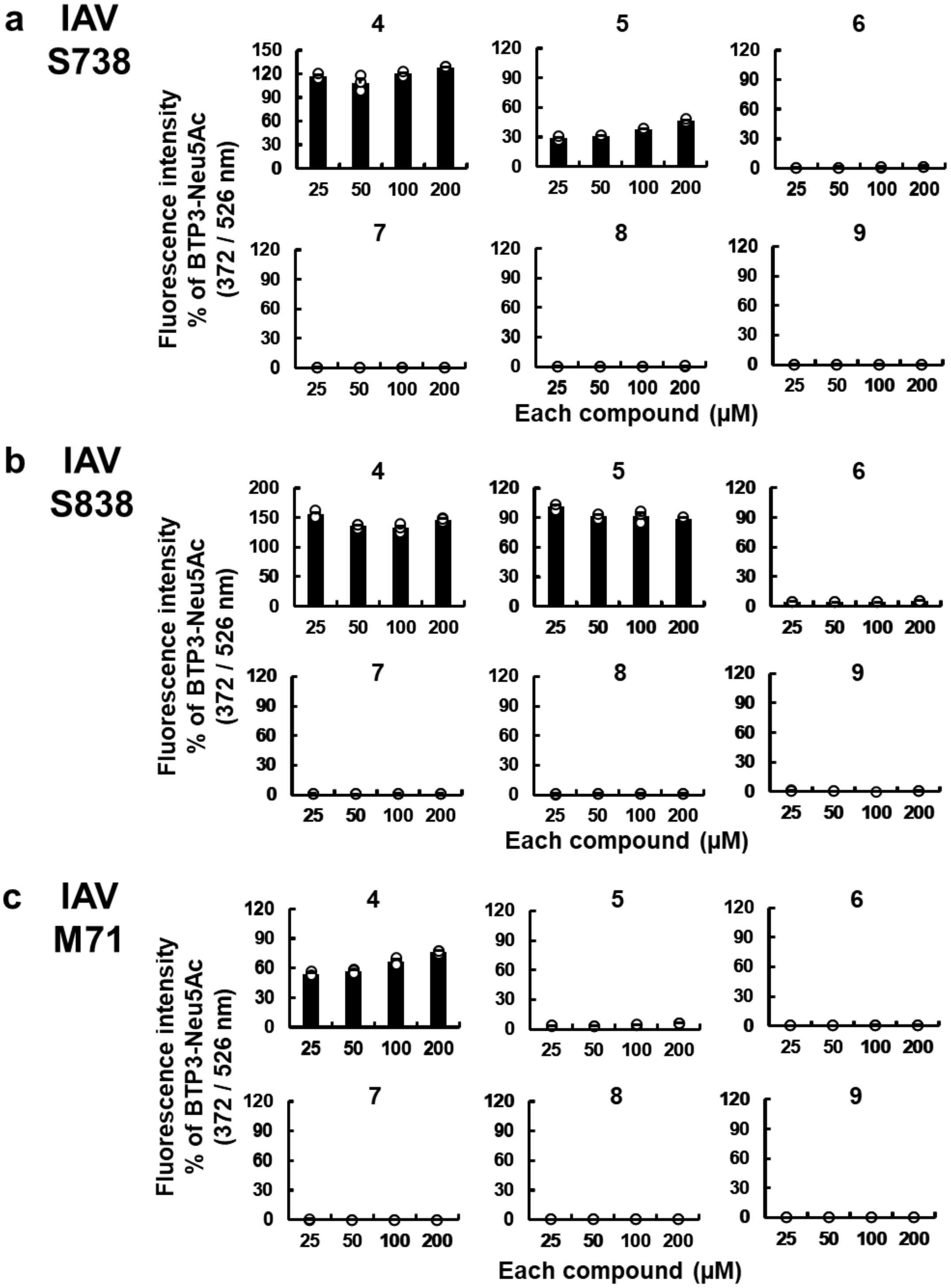

Supplementary Figure 6: Concentration-dependent comparison of the sialidase activities of Compounds **4–9** in IAV-NA. **a–c** 293T cells were transfected with expression vectors for oseltamivir-resistant IAV-N1NA (IAV S738 strain) (**a**), 2009 pandemic IAV-N1NA (IAV S838

strain) (**b**), and IAV-N2NA (IAV M71 strain) (**c**). Each set of NA-expressing cells was incubated with 25–200 μM of either BTP3-Neu5Ac or each compound at 37°C for 20 min. Sialidase activity is expressed as fluorescence intensity relative to that obtained with BTP3-Neu5Ac (set as 100%).

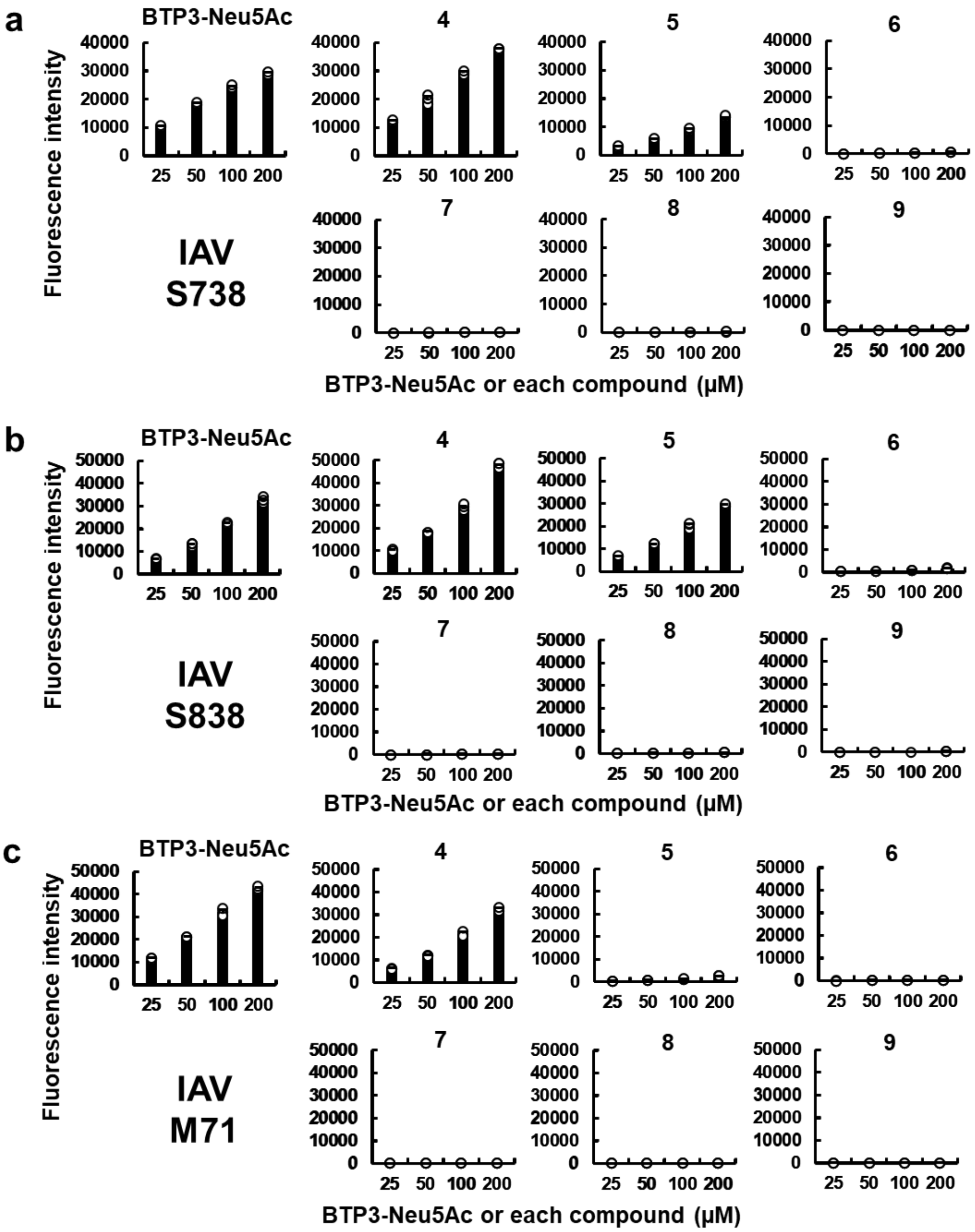

Supplementary Figure 7: Comparison of Compounds **4**–**9** for the sialidase activity of other IAV-NAs in a concentration-dependent manner. **a**–**c** 293T cells were transfected with expression vectors for oseltamivir-resistant IAV-N1NA (IAV S738 strain) (**a**), 2009 pandemic IAV-N1NA (IAV S838 strain) (**b**), and IAV-N2NA (IAV M71 strain) (**c**). Each set of NA-expressing cells

was incubated with 25–200 μM of either BTP3-Neu5Ac or each compound at 37°C for 20 min.

Sialidase activity is expressed as the fluorescence intensity of BTP3.

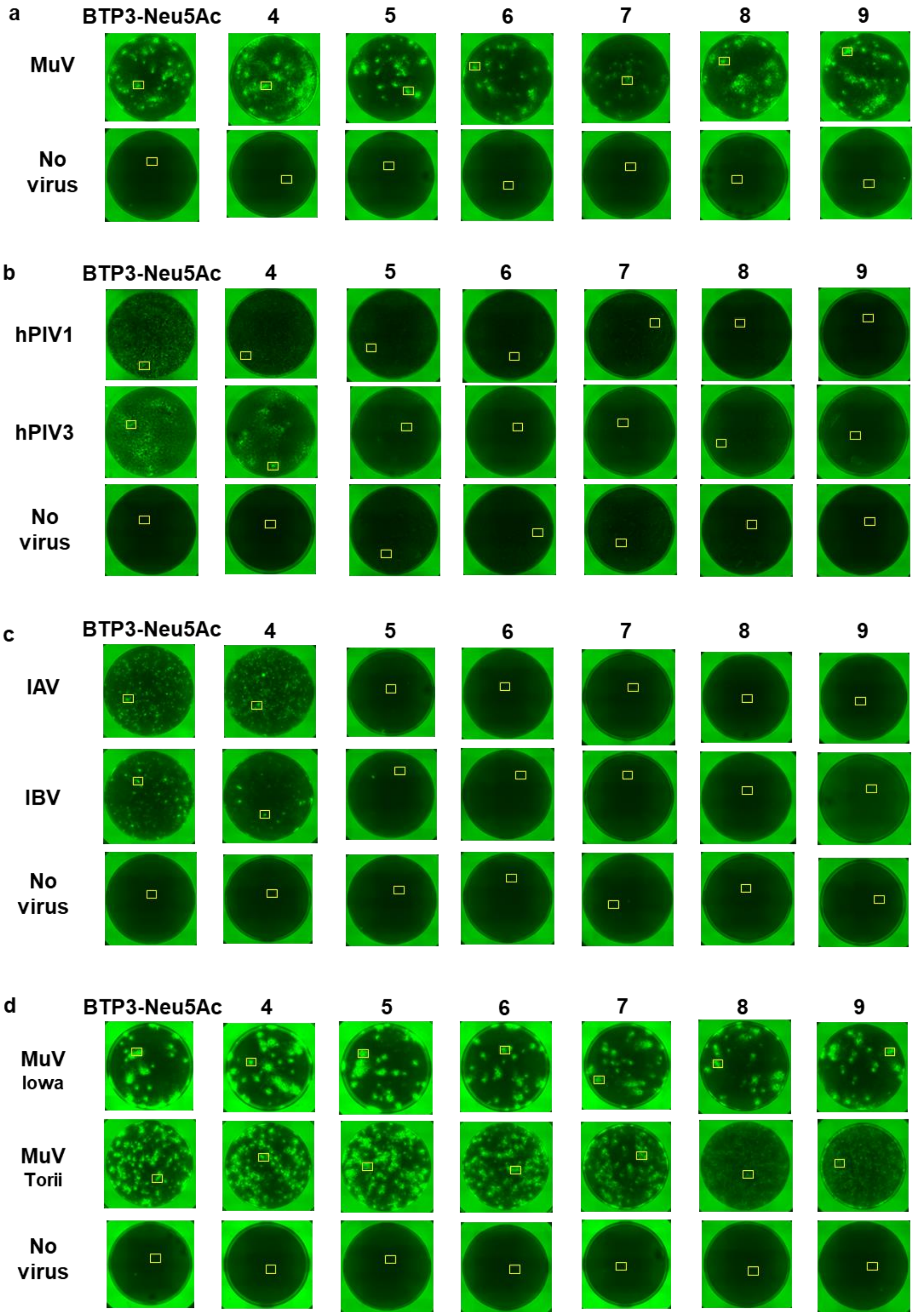

Supplementary Figure 8: Virus-infected cells visualized using Compounds **4–9**. **a** Vero cells

were infected with MuV (13V165E2 strain) and cultured for 48 h. **b** LLC-MK2 cells were infected with hPIV1 or hPIV3 and cultured for 48 h. **c** MDCK cells were infected with IAV (IAV PR8 strain) or IBV and cultured for 24 h. **d** Vero cells were infected with two other MuV (Torii and Iowa. US/2006 strains) and cultured for 48 h. All infected cells were incubated with 200 μM of either BTP3-Neu5Ac or each compound at 37°C for 20 min. Virus-infected cells in the microplate wells were visualized under UV irradiation. The fluorescence image of cells within the highlighted square region is shown in Fig. 7, and this supplementary figure depicts the entire well.

Methods

Synthesis of Compounds 1–9

I. General

All chemicals were purchased from Tokyo Chemical Industry Co., Ltd., Wako Pure Chemical Industries, Ltd., or Sigma-Aldrich Co. LLC., and used without further purification. ^{1}H-NMR and ^{13}C-NMR experiments were conducted on a JMTC-600 (JEOL Ltd.) NMR spectrometer with D_2O, CD_3OD or $CDCl_3$ as solvent. Chemical shifts were expressed in parts per million (ppm, δ) relative to the residual deuterated solvent or the internal standard tetramethylsilane. High-resolution mass spectra (HRMS) were measured on AccuTOF (JMS-T100LC) equipped with an electrospray ion source (JEOL Ltd.). Flash column chromatography was performed using silica gel 60N (spherical, neutral, 40–50 μm) or CHROMATOREX PSQ 60B. The analytical high-performance liquid chromatography (HPLC) system consisted of a HITACHI Pump Unit L-2130, a HITACHI L-2400 Absorbance Detector, and a HITACHI D-2500 Chromato-Integrator. The preparative HPLC system comprised a SHIMADZU Pump Unit LC-20AP, a SHIMADZU In-Line Degasser DGU-20A5R, and a SHIMADZU SPD-20A absorbance detector. Both the absorbance detectors were operated at 254 nm. The mobile phase for preparation and analysis comprised combinations of water (A), acetonitrile (B), and 100 mM aqueous ammonia (C), and the flow rates were 8 and 1 mL/min, respectively. YMC-Actus Triart

C18 (S-5 μm, 12 nm) columns were used for preparation (250 × 20 mm I.D.) and analysis (250 × 4.6 mm I.D.). To validate the chemical structures and purities of the compounds, nuclear magnetic resonance (NMR) data are provided in the Supplementary Information.

II. General methods and detailed experimental procedures for the synthesis of probes

II-I. Synthesis of *N*-deacetylated ammonium salt as a common intermediate

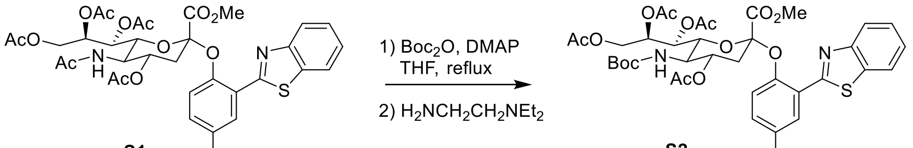

S1 S2

Methyl 4,7,8,9-tetra-*O*-acetyl-2-*O*-[2-(benzo[*d*]thiazol-2-yl)-4-bromophenyl]-*N*-*tert*-butoxycarbonyl-α-D-neuraminate (S2)

To a solution of methyl 2-[(benzothiazol-2-yl)-4-bromophenyl]-5-acetamido-4,7,8,9-tetra-*O*-acetyl-3,5-dideoxy-D-*glycero*-α-D-*galacto*-2-nonulopyranosylonate (**S1**)[supplementary reference 1 (SR1)] (6.42 g, 8.23 mmol) in tetrahydrofuran (THF) (70 ml), di-*tert*-butyl dicarbonate (Boc_2O) (3.59 g, 2.0 eq.) and 4-dimethylaminopyridine (DMAP) (100 mg, 0.1 eq.) were added and refluxed for 16 h. After cooling to room temperature, the resulting reaction mixture was mixed with *N,N*-diethylethlenediamine twice (first time: 3.5 mmol *N,N*-diethylethlenediamine, 3.0 eq. and second time: 2.4 mL *N,N*-diethylethlenediamine, 2.0 eq.) every 18–24 h. After a complete disappearance of *N*-Ac-*N*-Boc derivative on thin-layer chromatography (TLC), the reaction

mixture was diluted with EtOAc, and the organic layer was washed with 10% citric acid aqueous solution (three times) and water and brine, dried over Na_2SO_4, filtered off the drying agent, and concentrated under reduced pressure. The residue was purified by silica gel column chromatography (silica gel: 200 mL, *n*-hexane: EtOAc = 2:1–1:1 as an eluent) to yield 2.45 g of the desired product at 36% yield as a colorless foam.

^{1}H-NMR (600 MHz, $CDCl_3$) δ 8.59 (d, *J* = 2.8 Hz, 1 H), 8.08 (d, *J* = 8.2 Hz, 1 H), 7.94 (d, *J* = 7.9 Hz, 1 H), 7.54-7.49 (m, 2 H), 7.41-7.39 (Multiplet looks like td., 1 H), 7.28 (d, *J* = 8.9 Hz, 1 H), 5.48 (dd, *J* = 9.6, 0.9 Hz, 1 H), 5.43-5.40 (m, 1 H), 4.92 (td, *J* = 11.4, 4.0 Hz, 1 H), 4.52 (d, *J* = 11.0 Hz, 1 H), 4.32 (d, *J* = 10.4 Hz, 1 H), 4.25 (dd, *J* = 12.5, 2.7 Hz, 1 H), 4.14 (dd, *J* = 12.5, 4.9 Hz, 1 H), 3.87 (q, *J* = 10.5 Hz, 1 H), 3.53 (s, 3 H), 2.99 (dd, *J* = 12.9, 4.5 Hz, 1 H), 2.55 (t, *J* = 12.7 Hz, 1 H), 2.18 (s, 3 H), 2.11 (s, 3 H), 2.08 (s, 3 H), 2.06 (s, 3 H), 1.40 (s, 9 H).

^{13}C-NMR (150 MHz, $CDCl_3$) δ 202.0, 201.9, 201.4, 201.0, 198.9, 192.3, 186.4, 183.2, 182.3, 167.4, 165.7, 163.5, 157.6, 157.0, 156.5, 154.5, 152.6, 151.5, 148.2, 132.3, 111.6, 105.2, 99.9, 99.5, 98.2, 93.4, 84.5, 81.9, 70.3, 59.4, 52.3, 52.1, 52.1, 52.0.

MS (ESI$^+$) *m/z* 861 [M+Na]$^+$, 859 [M+Na]$^+$.

HRMS (ESI-TOF) *m/z* Calcd for $C_{36}H_{41}{}^{81}BrN_2NaO_{14}S$ [M+Na]$^+$ 861.13391; Found 861.13193, Calcd for $C_{36}H_{41}{}^{79}BrN_2NaO_{14}S$ [M+Na]$^+$ 859.13596; Found 859.13463.

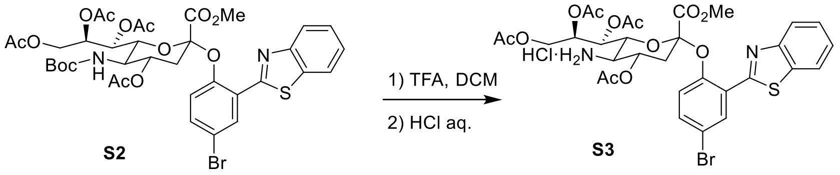

Methyl 4,7,8,9-tetra-*O*-acetyl-2-*O*-[2-(benzo[*d*]thiazol-2-yl)-4-bromophenyl]-α-D-neuraminate hydrochloride (S3)

To a solution of **S2** (2.45 g, 2.93 mmol) in dichloromethane (DCM) (10 mL), trifluoroacetic acid (TFA) (10 mL) was added and stirred at room temperature for 1 h. The resulting reaction mixture was concentrated under reduced pressure, and the resulting residue was dissolved in a mixture of 1,4-dioxane and 1 M aqueous HCl and concentrated under reduced pressure to obtain the HCl salt. The obtained HCl salt was dissolved in THF (26 mL) to yield **S3** solution (225 mmol/2 mL), which was used for subsequent reactions without further purification.

II-II. General procedure for the acylation of nitrogen and deacetylation of oxygen to produce tetraol (S4)

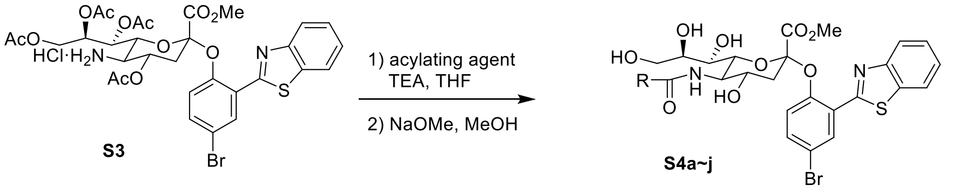

	acylating agent	R
**S4a**	: $(CH_3CH_2CO)_2O$	$-CH_2CH_3$
**S4b**	: $(CH_3CH_2CH_2CO)_2O$	$-(CH_2)_2CH_3$
**S4c**	: $CH_3(CH_2)_3COCl$	$-(CH_2)_3CH_3$
**S4d**	: $CH_3(CH_2)_4COCl$	$-(CH_2)_4CH_3$
**S4e**	: $CH_3(CH_2)_5COCl$	$-(CH_2)_5CH_3$
**S4f**	: $CH_3(CH_2)_6COCl$	$-(CH_2)_6CH_3$
**S4g**	: $(iPrCO)_2O$	-iPr
**S4h**	: tBuCOCl	-tBu
**S4i**	: BzCl	-Ph

II-II-I. Synthetic procedure for S4a is described below as a typical procedure for acylation reaction and the subsequent deacetylation reaction

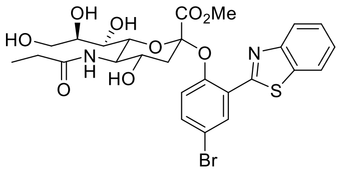

Methyl 2-*O*-[2-(benzo[*d*]thiazol-2-yl)-4-bromophenyl]-*N*-propanoyl-α-D-neuraminate (S4a)

To a solution of **S3** in THF (2 mL, 225 mmol/2 mL), which was described in the synthesis section of **S3**, triethylamine (TEA) (300 μL, 2.2 mmol) and propionic anhydride (120 μL, 932 mmol) were successively added and stirred at room temperature for 10 h. The resulting reaction mixture was diluted with EtOAc, and the organic layer was washed successively with water, diluted with $NaHCO_3$ aq. and water and brine, dried over Na_2SO_4, filtered, and concentrated under reduced pressure. The obtained residue was dissolved in NaOMe/MeOH solution (4 mL, 0.25 M NaOMe in MeOH), stirred at room temperature for 2 h, quenched by adding Amberlite FPC-3500 ion-exchange resin (0.6 g), filtered off the resin, and concentrated under reduced pressure to yield the desired tetraol **S4a**. The obtained crude product was dissolved in MeOH (8 mL) and used for subsequent reactions without further purification.

^{1}H-NMR (600 MHz, CD_3OD) δ 8.45 (d, *J* = 2.2 Hz, 1 H), 8.01 (d, *J* = 8.2 Hz, 1 H), 7.97 (d, *J* = 8.0 Hz, 1 H), 7.55 (d, *J* = 8.8 Hz, 1 H), 7.53 (dd, *J* = 9.0, 2.3 Hz, 1 H), 7.49 (ddd, *J* = 8.2, 7.2, 1.0 Hz, 1 H), 7.40 (ddd, *J* = 8.1, 7.2, 0.8 Hz, 1 H), 4.10 (dd, *J* = 10.6, 1.3 Hz, 1 H), 3.90 (t, *J* = 10.4 Hz, 1 H), 3.82-3.74 (m, 3 H), 3.60 (dd, *J* = 11.9, 6.1 Hz, 1 H), 3.54 (s, 3 H), 3.49 (dd, *J* = 9.1, 1.1 Hz, 1 H), 2.96 (dd, *J* = 12.8, 4.6 Hz, 1 H), 2.26-2.21 (m, 3 H), 1.10 (t, *J* = 7.6 Hz, 3 H).

MS (ESI$^+$) *m/z* 649 [M+Na]$^+$, 647 [M+Na]$^+$.

HRMS (ESI-TOF): *m/z* Calcd. for $C_{26}H_{29}{}^{81}BrN_2NaO_9S$ [M+Na]$^+$ 649.06544; Found. 649.06827, Calcd. for $C_{26}H_{29}{}^{79}BrN_2NaO_9S$ [M+Na]$^+$ 647.06748; Found. 647.06979.

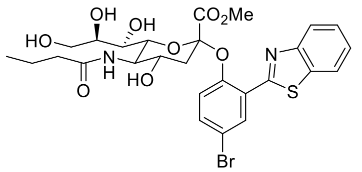

Methyl [2-(benzo[*d*]thiazol-2-yl)-4-bromophenyl]-*N*-butanoyl-α-D-neuraminate (S4b)

^{1}H-NMR (600 MHz, CD_3OD) δ 8.45 (d, *J* = 2.1 Hz, 1 H), 8.01 (d, *J* = 8.2 Hz, 1 H), 7.97 (d, *J* = 8.0 Hz, 1 H), 7.55 (d, *J* = 8.9 Hz, 1 H), 7.53 (dd, *J* = 8.9, 2.2 Hz, 1 H), 7.49 (t, *J* = 7.6 Hz, 1 H), 7.40 (t, *J* = 7.6 Hz, 1 H), 4.09 (dd, *J* = 10.6, 1.4 Hz, 1 H), 3.90 (t, *J* = 10.4 Hz, 1 H), 3.82-3.78 (m, 2 H), 3.76 (ddd, *J* = 11.8, 10.2, 4.6 Hz, 1 H), 3.59 (dd, *J* = 11.9, 6.1 Hz, 1 H), 3.54 (s, 3 H), 3.50 (dd, *J* = 9.1, 1.2 Hz, 1 H), 2.96 (dd, *J* = 12.8, 4.6 Hz, 1 H), 2.24 (d, *J* = 12.4 Hz, 1 H), 2.22-2.16 (m, 2 H), 1.62 (sex, *J* = 7.4 Hz, 2 H), 0.92 (t, *J* = 7.4 Hz, 3 H).

MS (ESI$^+$) *m/z* 663 [M+Na]$^+$, 661 [M+Na]$^+$.

HRMS (ESI-TOF): *m/z* Calcd. for $C_{27}H_{31}{}^{81}BrN_2NaO_9S$ [M+Na]$^+$ 663.08109; Found. 663.08249, Calcd. for $C_{27}H_{31}{}^{79}BrN_2NaO_9S$ [M+Na]$^+$ 661.08313; Found. 661.08413.

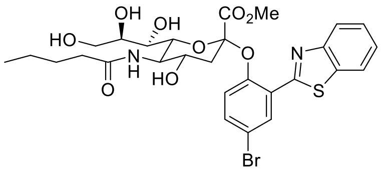

Methyl [2-(benzo[*d*]thiazol-2-yl)-4-bromophenyl]-*N*-penanoyl-α-D-neuraminate (S4c)

^{1}H-NMR (600 MHz, CD_3OD) δ 8.46 (d, *J* = 2.2 Hz, 1 H), 8.01 (d, *J* = 8.2 Hz, 1 H), 7.98 (d, *J* = 8.0 Hz, 1 H), 7.56 (d, *J* = 8.8 Hz, 1 H), 7.53 (dd, *J* = 8.9, 2.2 Hz, 1 H), 7.50 (ddd, *J* = 8.1, 7.2, 1.0 Hz, 1 H), 7.41 (ddd, *J* = 8.0, 7.2, 0.9 Hz, 1 H), 4.09 (dd, *J* = 10.7, 1.4 Hz, 1 H), 3.89 (t, *J* = 10.4 Hz, 1 H), 7.39-2.16 (m, 2 H), 3.75 (ddd, *J* = 11.8, 10.2, 4.6 Hz, 1 H), 3.59 (dd, *J* = 12.0, 6.2 Hz, 1 H), 3.54 (s, 3 H), 3.49 (dd, *J* = 9.0, 1.1 Hz, 1 H), 2.95 (dd, *J* = 12.7, 4.6 Hz, 1 H), 2.25-2.21 (m, 3 H), 1.57 (quin, *J* = 7.7 Hz, 2 H), 1.33 (sex, *J* = 7.4 Hz, 2 H), 0.89 (t, *J* = 7.4 Hz, 3 H).

MS (ESI$^+$) *m/z* 677 [M+Na]$^+$, 675 [M+Na]$^+$.

HRMS (ESI-TOF): *m/z* Calcd. for $C_{28}H_{33}{}^{81}BrN_2NaO_9S$ [M+Na]+ 677.09674; Found. 677.09797, Calcd. for $C_{28}H_{33}{}^{79}BrN_2NaO_9S$ [M+Na]+ 675.09879; Found. 675.09985.

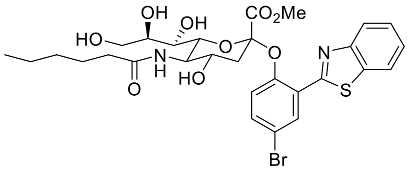

Methyl [2-(benzo[*d*]thiazol-2-yl)-4-bromophenyl]-*N*-hexanoyl-α-D-neuraminate (S4d)

^{1}H-NMR (600 MHz, CD_3OD) δ 8.46 (d, *J* = 2.2 Hz, 1 H), 8.02 (d, *J* = 8.2 Hz, 1 H), 7.98 (d, *J* = 7.9 Hz, 1 H), 7.56 (d, *J* = 8.9 Hz, 1 H), 7.53 (dd, *J* = 9.0, 2.3 Hz, 1 H), 7.50 (ddd, *J* = 8.1, 7.2, 1.0 Hz, 1 H), 7.41 (ddd, *J* = 8.0, 7.2, 0.9 Hz, 1 H), 4.09 (dd, *J* = 10.7, 1.3 Hz, 1 H), 3.89 (t, *J* = 10.4 Hz, 1 H), 3.82-3.79 (m, 2 H), 3.75 (ddd, *J* = 11.8, 10.2, 4.6 Hz, 1 H), 3.58 (dd, *J* = 12.0, 6.4 Hz, 1 H), 3.54 (s, 3 H), 3.49 (dd, *J* = 9.0, 1.2 Hz, 1 H), 2.96 (dd, *J* = 12.7, 4.6 Hz, 1 H), 2.25-2.20 (m, 3 H), 1.59 (quin, *J* = 7.2 Hz, 2 H), 1.34-1.25 (m, 4 H), 0.87 (t, *J* = 7.0 Hz, 3 H).

MS (ESI^{+}) *m/z* 691 [M+Na]$^{+}$, 689 [M+Na]$^{+}$.

HRMS (ESI-TOF): *m/z* Calcd. for $C_{29}H_{35}{}^{81}BrN_2NaO_9S$ [M+Na]$^{+}$ 691.11239; Found. 691.11027, Calcd. for $C_{29}H_{35}{}^{79}BrN_2NaO_9S$ [M+Na]$^{+}$ 689.11443; Found. 689.11209.

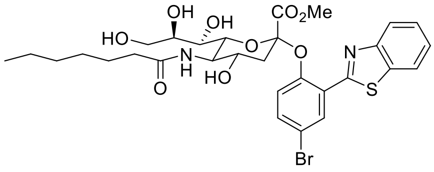

Methyl [2-(benzo[*d*]thiazol-2-yl)-4-bromophenyl]-*N*-heptanoyl-α-D-neuraminate (S4e)

^{1}H-NMR (600 MHz, CD_3OD) δ 8.45 (d, *J* = 1.7 Hz, 1 H), 8.01 (d, *J* = 8.1 Hz, 1 H), 7.98 (d, *J* = 7.9 Hz, 1 H), 7.55 (d, *J* = 8.9 Hz, 1 H), 7.53 (dd, *J* = 8.9, 2.3 Hz, 1 H), 7.50 (t, *J* = 7.6 Hz, 1 H),

7.40 (t, J = 7.5 Hz, 1 H), 4.09 (dd, J = 10.6, 1.2 Hz, 1 H), 3.90 (t, J = 10.4 Hz, 1 H), 3.82-3.79 (m, 2 H), 3.75 (ddd, J = 11.6, 10.3, 4.5 Hz, 1 H), 3.58 (dd, J = 12.1, 6.5 Hz, 1 H), 3.54 (s, 3 H), 3.48 (d, J = 9.0 Hz, 1 H), 2.96 (dd, J = 12.8, 4.6 Hz, 1 H), 2.25-2.20 (m, 3 H), 1.58 (quin, J = 7.3 Hz, 2 H), 1.33-1.23 (m, 6 H), 0.86 (t, J = 6.9 Hz, 3 H).

MS (ESI^+) *m/z* 705 $[M+Na]^+$, 703 $[M+Na]^+$.

HRMS (ESI-TOF): *m/z* Calcd. for $C_{30}H_{37}{}^{81}BrN_2NaO_9S$ $[M+Na]^+$ 705.12804; Found. 705.12917, Calcd. for $C_{30}H_{37}{}^{79}BrN_2NaO_9S$ $[M+Na]^+$ 703.13008; Found. 703.13090.

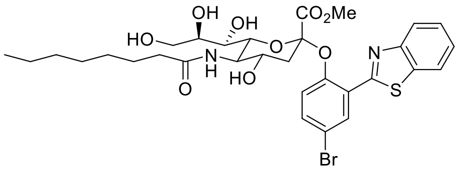

Methyl [2-(benzo[*d*]thiazol-2-yl)-4-bromophenyl]-*N*-octanoyl-α-D-neuraminate (S4f)

^{1}H-NMR (600 MHz, CD_3OD) δ 8.46 (d, J = 2.1 Hz, 1 H), 8.02 (d, J = 8.2 Hz, 1 H), 7.98 (d, J = 8.0 Hz, 1 H), 7.56 (d, J = 8.8 Hz, 1 H), 7.53 (dd, J = 8.9, 2.2 Hz, 1 H), 7.50 (ddd, J = 8.1, 7.2, 1.0 Hz, 1 H), 7.41 (ddd, J = 8.0, 7.2, 0.9 Hz, 1 H), 4.09 (dd, J = 10.6, 1.3 Hz, 1 H), 3.89 (t, J = 10.4 Hz, 1 H), 3.82-3.79 (m, 2 H), 3.75 (ddd, J = 11.7, 10.2, 4.5 Hz, 1 H), 3.58 (dd, J = 12.1, 6.4 Hz, 1 H), 3.54 (s, 3 H), 3.48 (dd, J = 8.9, 1.3 Hz, 1 H), 2.96 (dd, J = 12.8, 4.6 Hz, 1 H), 2.25-2.19 (m, 3 H), 1.58 (quin, J = 7.2 Hz, 2 H), 1.31-1.23 (m, 8 H), 0.85 (t, J = 7.0 Hz, 3 H).

MS (ESI^+) *m/z* 719 $[M+Na]^+$, 717 $[M+Na]^+$.

HRMS (ESI-TOF): *m/z* Calcd. for $C_{31}H_{39}{}^{81}BrN_2NaO_9S$ [M+Na]+ 719.14369; Found. 719.14303, Calcd. for $C_{31}H_{39}{}^{79}BrN_2NaO_9S$ [M+Na]+ 717.14573; Found. 717.14458.

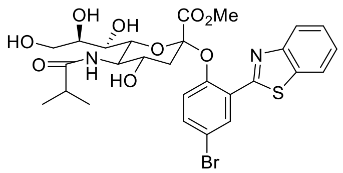

Methyl [2-(benzo[*d*]thiazol-2-yl)-4-bromophenyl]-*N*-isobutyryl-α-D-neuraminate (S4g)

^{1}H-NMR (600 MHz, CD_3OD) δ 8.46 (d, *J* = 2.2 Hz, 1 H), 8.02 (d, *J* = 8.2 Hz, 1 H), 7.98 (d, *J* = 7.9 Hz, 1 H), 7.56 (d, *J* = 8.9 Hz, 1 H), 7.53 (dd, *J* = 8.9, 2.4 Hz, 1 H), 7.50 (ddd, *J* = 8.2, 7.2, 1.1 Hz, 1 H), 7.41 (ddd, *J* = 8.1, 7.1, 0.9 Hz, 1 H), 4.09 (dd, *J* = 10.6, 1.4 Hz, 1 H), 3.87 (t, *J* = 10.4 Hz, 1 H), 3.81-3.75 (m, 3 H), 3.59 (q, *J* = 5.9 Hz, 1 H), 3.55 (s, 3 H), 3.47 (dd, *J* = 9.1, 1.2 Hz, 1 H), 2.96 (dd, *J* = 12.8, 4.6 Hz, 1 H), 2.47 (sep, *J* = 6.9 Hz, 1 H), 2.23 (t, *J* = 12.3 Hz, 1 H), 1.10 (d, *J* = 6.9 Hz, 3 H), 1.10 (d, *J* = 6.9 Hz, 3 H).

MS (ESI$^+$) *m/z* 663 $[M+Na]^+$, 661 $[M+Na]^+$.

HRMS (ESI-TOF): *m/z* Calcd. for $C_{27}H_{31}{}^{81}BrN_2NaO_9S$ $[M+Na]^+$ 663.08109; Found. 663.08097, Calcd. for $C_{27}H_{31}{}^{79}BrN_2NaO_9S$ $[M+Na]^+$ 661.08313; Found. 661.08258.

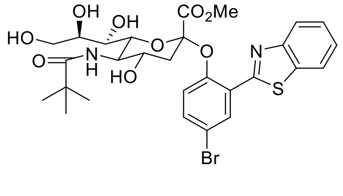

Methyl [2-(benzo[*d*]thiazol-2-yl)-4-bromophenyl]-*N*-pivaloyl-α-D-neuraminate (S4h)

^{1}H-NMR (600 MHz, CD_3OD) δ 8.45 (d, *J* = 2.3 Hz, 1 H), 8.02 (d, *J* = 8.2 Hz, 1 H), 7.98 (d, *J* = 8.0 Hz, 1 H), 7.57 (d, *J* = 8.9 Hz, 1 H), 7.54 (dd, *J* = 8.9, 2.4 Hz, 1 H), 7.50 (ddd, *J* = 8.1, 7.2, 0.9 Hz, 1 H), 7.41 (ddd, *J* = 8.1, 7.1, 0.8 Hz, 1 H), 4.08 (d, *J* = 9.0 Hz, 1 H), 3.92-3.86 (m, 2 H), 3.82-3.78 (m, 2 H), 3.59 (dd, *J* = 11.6, 5.6 Hz, 1 H), 3.55 (s, 3 H), 3.44 (d, *J* = 9.3 Hz, 1 H), 2.98 (dd, *J* = 12.7, 3.6 Hz, 1 H), 2.22 (t, *J* = 12.1 Hz, 1 H), 1.17 (s, 9 H).

MS (ESI$^+$) *m/z* 677 [M+Na]$^+$, 675 [M+Na]$^+$.

HRMS (ESI-TOF): *m/z* Calcd. for $C_{28}H_{33}{}^{81}BrN_2NaO_9S$ [M+Na]$^+$ 677.09674; Found. 677.09844, Calcd. for $C_{28}H_{33}{}^{79}BrN_2NaO_9S$ [M+Na]$^+$ 675.09878; Found. 675.10030.

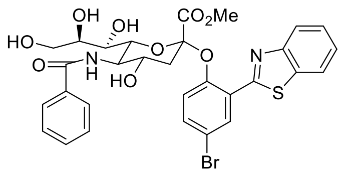

Methyl [2-(benzo[*d*]thiazol-2-yl)-4-bromophenyl]-*N*-benzoyl-α-D-neuraminate (S4i)

^{1}H-NMR (600 MHz, CD_3OD) δ 8.44 (d, *J* = 2.5 Hz, 1 H), 7.99 (d, *J* = 8.2 Hz, 1 H), 7.96 (d, *J* = 8.0 Hz, 1 H), 7.81 (d, *J* = 7.2 Hz, 2 H), 7.56 (d, *J* = 8.9 Hz, 1 H), 7.51 (dd, *J* = 8.9, 2.5 Hz, 1 H), 7.48-7.45 (m, 2 H), 7.39-7.36 (m, 3 H), 4.21 (dd, *J* = 10.7, 1.2 Hz, 1 H), 4.12 (t, *J* = 10.3 Hz, 1 H), 3.92 (ddd, *J* = 11.9, 10.1, 4.5 Hz, 1 H), 3.78 (ddd, *J* = 9.0, 5.7, 3.0 Hz, 1 H), 3.74 (dd, *J* =

11.4, 2.7 Hz, 1 H), 3.56 (dd, J = 11.4, 5.4 Hz, 1 H), 3.54-3.51 (m, 4 H), 2.98 (dd, J = 12.7, 4.6 Hz, 1 H), 2.25 (t, J = 12.3 Hz, 1 H).

MS (ESI$^+$) *m/z* 697 [M+Na]$^+$, 695 [M+Na]$^+$.

HRMS (ESI-TOF): *m/z* Calcd. for $C_{30}H_{29}{}^{81}BrN_2NaO_9S$ [M+Na]$^+$ 697.06544; Found. 697.06542, Calcd. for $C_{30}H_{29}{}^{79}BrN_2NaO_9S$ [M+Na]$^+$ 695.06748; Found. 695.06771.

II-III. General procedure for hydrolysis of methyl ester to give caroboxylic acid (S5, Compounds 1–9)

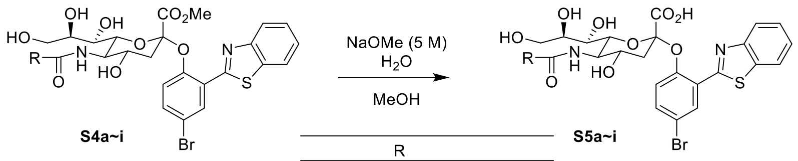

	R	
**S5a**	: -CH_2CH_3	Compound 3
**S5b**	: -$(CH_2)_2CH_3$	Compound 4
**S5c**	: -$(CH_2)_3CH_3$	Compound 5
**S5d**	: -$(CH_2)_4CH_3$	Compound 6
**S5e**	: -$(CH_2)_5CH_3$	Compound 7
**S5f**	: -$(CH_2)_6CH_3$	Compound 8
**S5g**	: -iPr	Compound 1
**S5h**	: -tBu	Compound 2
**S5i**	: -Ph	Compound 3

II-III-I. Synthetic procedure for S5a (Compound 4) is described below as a typical procedure for hydrolysis of methyl ester S4.

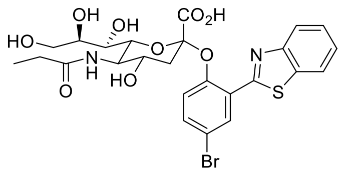

2-*O*-[2-(benzo[*d*]thiazol-2-yl)-4-bromophenyl]-*N*-propanoyl-α-D-neuraminic acid (S5a, Compound 4)

The prepared MeOH solution of **S4a** (6 mL), which was described in paragraph II-II-I, was concentrated under reduced pressure to a volume of 1 mL. The resulting MeOH solution was added successively H_2O (400 μL) and NaOMe (200 μL, 5 mol/L in MeOH), stirred at room temperature for 17 h, added AcOH (50 μL) to quench the reaction and purified by preparative HPLC to yield the desired **S5a** (60.6 mg) in 57% yield for 4 steps as a colorless amorphous.

^{1}H-NMR (600 MHz, D_2O) δ 7.81 (d, *J* = 2.1 Hz, 1 H), 7.73 (d, *J* = 8.2 Hz, 2 H), 7.32 (t, *J* = 7.7 Hz, 1 H), 7.25 (dd, *J* = 8.8, 2.2 Hz, 1 H), 7.21 (t, *J* = 7.6 Hz, 1 H), 7.11 (d, *J* = 8.9 Hz, 1 H), 3.81-3.78 (m, 2 H), 3.72-3.69 (m, 2 H), 3.64-3.59 (m, 1 H), 3.51 (dd, *J* = 11.8, 5.8 Hz, 1 H), 3.43 (d, *J* = 9.1 Hz, 1 H), 2.81 (dd, *J* = 12.4, 4.6 Hz, 1 H), 2.16 (q, *J* = 7.6 Hz, 2 H), 1.93 (t, *J* = 12.2 Hz, 1 H), 0.98 (t, *J* = 7.6 Hz, 3 H).

MS (ESI$^+$) *m/z* 635 [M+Na]$^+$, 633 [M+Na]$^+$.

HRMS (ESI-TOF): *m/z* Calcd. for $C_{25}H_{27}{}^{81}BrN_2NaO_9S$ [M+Na]$^+$ 635.04979; Found. 635.04874, Calcd. for $C_{25}H_{27}{}^{79}BrN_2NaO_9S$ [M+Na]$^+$ 633.05183; Found. 633.05009.

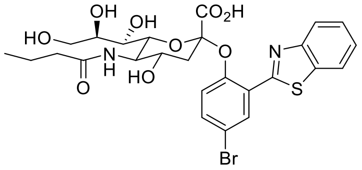

[2-(benzo[*d*]thiazol-2-yl)-4-bromophenyl]-*N*-butanoyl-α-D-neuraminic acid (S5b, Compound 5)

Yield: 65% (70.2 mg, colorless amorphous)

^{1}H-NMR (600 MHz, D_2O) δ 7.81 (d, *J* = 2.3 Hz, 1 H), 7.73 (d, *J* = 8.6 Hz, 2 H), 7.32 (t, *J* = 7.6 Hz, 1 H), 7.26 (dd, *J* = 8.9, 2.3 Hz, 1 H), 7.21 (t, *J* = 7.6 Hz, 1 H), 7.12 (d, *J* = 8.9 Hz, 1 H), 3.81-3.77 (m, 2 H), 3.72-3.69 (m, 2 H), 3.63-3.59 (m, 1 H), 3.50 (dd, *J* = 12.2, 6.2 Hz, 1 H), 3.45 (d, *J* = 9.0 Hz, 1 H), 2.81 (dd, *J* = 12.4, 4.6 Hz, 1 H), 2.12 (t, *J* = 7.2 Hz, 2 H), 1.93 (t, *J* = 12.1 Hz, 1 H), 1.47 (sex, *J* = 7.4 Hz, 2 H), 0.77 (t, *J* = 7.4 Hz, 3 H).

MS (ESI$^+$) *m/z* 649 $[M+Na]^+$, 647 $[M+Na]^+$.

HRMS (ESI-TOF): *m/z* Calcd. for $C_{26}H_{29}{}^{81}BrN_2NaO_9S$ $[M+Na]^+$ 649.06544; Found. 649.06380, Calcd. for $C_{26}H_{29}{}^{79}BrN_2NaO_9S$ $[M+Na]^+$ 647.06749; Found. 647.06519.

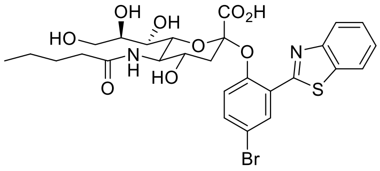

[2-(benzo[*d*]thiazol-2-yl)-4-bromophenyl]-*N*-penanoyl-α-D-neuraminic acid (S5c, Compound 6)

Yield: 64% (70.9 mg, colorless amorphous)

^{1}H-NMR (600 MHz, D_2O) δ 7.82 (s, 1 H), 7.73 (d, J = 8.1 Hz, 2 H), 7.32 (t, J = 7.6 Hz, 1 H), 7.26 (d, J = 8.8 Hz, 1 H), 7.21 (t, J = 7.6 Hz, 1 H), 7.12 (d, J = 8.9 Hz, 1 H), 3.80-3.76 (m, 2 H), 3.72-3.69 (m, 2 H), 3.63-3.59 (m, 1 H), 3.50 (dd, J = 12.0, 6.0 Hz, 1 H), 3.44 (d, J = 8.9 Hz, 1 H), 2.81 (dd, J = 12.4, 4.4 Hz, 1 H), 2.15 (t, J = 7.3 Hz, 2 H), 1.93 (t, J = 12.1 Hz, 1 H), 1.47-1.40 (m, 2 H), 1.17 (sex, J = 7.3 Hz, 2 H), 0.74 (t, J = 7.3 Hz, 3 H).

MS (ESI$^+$) *m/z* 663 $[M+Na]^+$, 661 $[M+Na]^+$.

HRMS (ESI-TOF): *m/z* Calcd. for $C_{27}H_{31}{}^{81}BrN_2NaO_9S$ $[M+Na]^+$ 663.08109; Found. 663.08082, Calcd. for $C_{27}H_{31}{}^{79}BrN_2NaO_9S$ $[M+Na]^+$ 661.08313; Found. 661.08218.

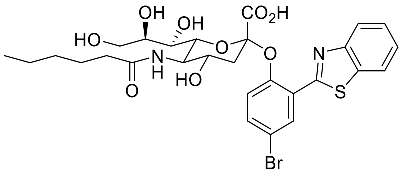

[2-(benzo[*d*]thiazol-2-yl)-4-bromophenyl]-*N*-hexanoyl-α-D-neuraminic acid (S5d, Compound 7)

Yield: 55% (61.8 mg, colorless amorphous)

^{1}H-NMR (600 MHz, D_2O) δ 7.84 (d, J = 2.5 Hz, 1 H), 7.75 (d, J = 7.6 Hz, 1 H), 7.74 (d, J = 7.9 Hz, 1 H), 7.33 (t, J = 7.7 Hz, 1 H), 7.27 (dd, J = 8.9, 2.5 Hz, 1 H), 7.22 (t, J = 7.7 Hz, 1 H), 7.13 (d, J = 8.9 Hz, 1 H), 3.80-3.76 (m, 2 H), 3.72-3.69 (m, 2 H), 3.63-3.59 (m, 1 H), 3.50 (dd, J =

12.0, 6.1 Hz, 1 H), 3.44 (d, J = 9.1 Hz, 1 H), 2.82 (dd, J = 12.5, 4.6 Hz, 1 H), 2.14 (t, J = 7.3 Hz, 2 H), 1.94 (t, J = 12.2 Hz, 1 H), 1.48-1.43 (m, 2 H), 1.17-1.10 (m, 4 H), 0.71 (t, J = 7.0 Hz, 3 H).

MS (ESI$^+$) *m/z* 677 $[M+Na]^+$, 675 $[M+Na]^+$, 655 $[M+H]^+$, 653 $[M+H]^+$.

HRMS (ESI-TOF): *m/z* Calcd. for $C_{28}H_{33}{}^{81}BrN_2NaO_9S$ $[M+Na]^+$ 677.09673; Found. 677.09554, Calcd. for $C_{28}H_{33}{}^{79}BrN_2NaO_9S$ $[M+Na]^+$ 675.09879; Found. 675.09888, Calcd. for $C_{28}H_{34}{}^{81}BrN_2O_9S$ $[M+H]^+$ 655.11479; Found. 655.11585, Calcd. for $C_{28}H_{34}{}^{79}BrN_2O_9S$ $[M+H]^+$ 653.11683; Found. 653.11603.

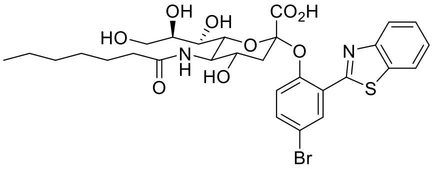

[2-(benzo[*d*]thiazol-2-yl)-4-bromophenyl]-*N*-hepanoyl-α-D-neuraminic acid (S5e, Compound 8)

Yield: 70% (80.8 mg, colorless amorphous)

^{1}H-NMR (600 MHz, D_2O) δ 7.83 (d, J = 2.4 Hz, 1 H), 7.74 (d, J = 7.9 Hz, 1 H), 7.73 (d, J = 8.0 Hz, 1 H), 7.32 (t, J = 7.7 Hz, 1 H), 7.27 (dd, J = 8.9, 2.4 Hz, 1 H), 7.21 (t, J = 7.6 Hz, 1 H), 7.13 (d, J = 8.8 Hz, 1 H), 3.79-3.75 (m, 2 H), 3.72-3.69 (m, 2 H), 3.64-3.59 (m, 1 H), 3.49 (dd, J = 12.4, 6.6 Hz, 1 H), 3.43 (dd, J = 8.9 Hz, 1 H), 2.82 (dd, J = 12.4, 4.6 Hz, 1 H), 2.14 (t, J = 7.3

Hz, 2 H), 1.94 (t, J = 12.1 Hz, 1 H), 1.48-1.40 (m, 2 H), 1.16-1.08 (m, 6 H), 0.70 (t, J = 6.7 Hz, 3 H).

MS (ESI$^+$) m/z 691 $[M+Na]^+$, 689 $[M+Na]^+$, 669 $[M+H]^+$, 667 $[M+H]^+$.

HRMS (ESI-TOF): m/z Calcd. for $C_{29}H_{35}{}^{81}BrN_2NaO_9S$ $[M+Na]^+$ 691.11238; Found. 691.11191, Calcd. for $C_{29}H_{35}{}^{79}BrN_2NaO_9S$ $[M+Na]^+$ 689.11443; Found. 689.11532, Calcd. for $C_{29}H_{36}{}^{81}BrN_2O_9S$ $[M+H]^+$ 669.13044; Found. 669.12800, Calcd. for $C_{29}H_{36}{}^{79}BrN_2O_9S$ $[M+H]^+$ 667.13249; Found. 667.13168.

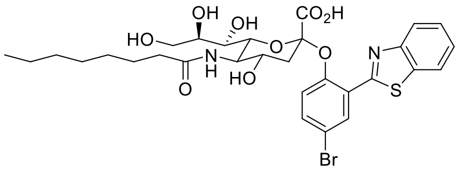

[2-(benzo[*d*]thiazol-2-yl)-4-bromophenyl]-*N*-octanoyl-α-D-neuraminic acid (S5f, Compound 9)

Yield: 69% (81.5 mg, colorless amorphous)

^{1}H-NMR (600 MHz, D_2O) δ 7.87 (s, 1 H), 7.77 (Two doublets look like triplet., J = 7.2 Hz, 2 H), 7.35 (t, J = 7.6 Hz, 1 H), 7.29 (d, J = 8.9 Hz, 1 H), 7.24 (t, J = 7.5 Hz, 1 H), 7.15 (d, J = 8.8 Hz, 1 H), 3.79-3.75 (m, 2 H), 3.72-3.69 (m, 2 H), 3.64-3.59 (m, 1 H), 3.49 (dd, J = 11.9, 6.3 Hz, 1 H), 3.42 (d, J = 8.9 Hz, 1 H), 2.82 (dd, J = 12.3, 4.2 Hz, 1 H), 2.13 (t, J = 7.1 Hz, 2 H), 1.95 (t, J = 12.1 Hz, 1 H), 1.47-1.40 (m, 2 H), 1.16-1.06 (m, 8 H), 0.69 (t, J = 6.1 Hz, 3 H).

MS (ESI$^+$) *m/z* 705 [M+Na]$^+$, 703 [M+Na]$^+$, 683 [M+H]$^+$, 681 [M+H]$^+$.

HRMS (ESI-TOF): *m/z* Calcd. for $C_{30}H_{37}{}^{81}BrN_2NaO_9S$ [M+Na]$^+$ 705.12803; Found. 705.12834, Calcd. for $C_{30}H_{37}{}^{79}BrN_2NaO_9S$ [M+Na]$^+$ 703.13008; Found. 703.13112, Calcd. for $C_{30}H_{38}{}^{81}BrN_2O_9S$ [M+H]$^+$ 683.14609; Found. 683.14775, Calcd. for $C_{30}H_{38}{}^{79}BrN_2O_9S$ [M+H]$^+$ 681.14814; Found. 681.14925.

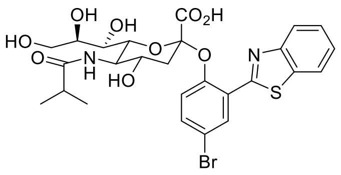

[2-(benzo[*d*]thiazol-2-yl)-4-bromophenyl]-*N*-isobutyroyl-α-D-neuraminic acid (S5g, Compound 1)

Yield: 67% (70.7 mg, colorless amorphous)

^{1}H-NMR (600 MHz, D_2O) δ 7.81 (s, 1 H), 7.73 (d, *J* = 8.0 Hz, 2 H), 7.32 (t, *J* = 7.6 Hz, 1 H), 7.25 (d, *J* = 8.9 Hz, 1 H), 7.21 (t, *J* = 7.6 Hz, 1 H), 7.11 (d, *J* = 8.8 Hz, 1 H), 3.81-3.76 (m, 2 H), 3.72-3.71 (m, 2 H), 3.65-3.61 (m, 1 H), 3.51 (dd, *J* = 12.4, 6.4 Hz, 1 H), 3.41 (d, *J* = 8.9 Hz, 1 H), 2.81 (dd, *J* = 12.4, 4.6 Hz, 1 H), 2.40 (sep, *J* = 6.9 Hz, 1 H), 1.91 (t, *J* = 12.2 Hz, 1 H), 0.98 (d, *J* = 6.4 Hz, 6 H).

MS (ESI$^+$) *m/z* 649 [M + Na]$^+$, 647 [M + Na]$^+$, 627 [M + H]$^+$, 625 [M + H]$^+$.

HRMS (ESI-TOF): *m/z* Calcd. for $C_{26}H_{29}{}^{81}BrN_2NaO_9S$ [M + Na]$^+$ 649.06544; Found. 649.06599, Calcd. for $C_{26}H_{29}{}^{79}BrN_2NaO_9S$ [M + Na]$^+$ 647.06748; Found. 647.06711, Calcd. for $C_{26}H_{30}{}^{81}BrN_2O_9S$ [M + H]$^+$ 627.08349; Found. 627.08510, Calcd. for $C_{26}H_{30}{}^{79}BrN_2O_9S$ [M + H]$^+$ 625.08554; Found. 625.09012.

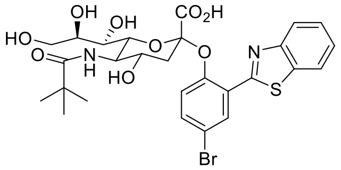

[2-(benzo[*d*]thiazol-2-yl)-4-bromophenyl]-*N*-pivaloyl-α-D-neuraminic acid (S5h, Compound 2)

Yield: 57% (62.1 mg, colorless amorphous)

^{1}H-NMR (600 MHz, D_2O) δ 7.81 (d, *J* = 2.3 Hz, 1 H), 7.74 (d, *J* = 8.1 Hz, 2 H), 7.34-7.31 (m, 1 H), 7.26 (dd, *J* = 9.0, 2.2 Hz, 1 H), 7.22 (t, *J* = 7.5 Hz, 1 H), 7.12 (d, *J* = 8.9 Hz, 1 H), 3.86-3.81 (m, 2 H), 3.72-3.71 (m, 3 H), 3.51 (dd, *J* = 12.5, 6.2 Hz, 1 H), 3.39 (d, *J* = 8.8 Hz, 1 H), 2.82 (dd, *J* = 12.4, 4.5 Hz, 1 H), 1.91 (t, *J* = 12.1 Hz, 1 H), 1.05 (s, 9 H).

MS (ESI$^+$) *m/z* 663 [M+Na]$^+$, 661 [M+Na]$^+$, 641 [M+H]$^+$, 639 [M+H]$^+$.

HRMS (ESI-TOF): *m/z* Calcd. for $C_{27}H_{31}{}^{81}BrN_2NaO_9S$ [M+Na]$^+$ 663.08109; Found. 663.08006, Calcd. for $C_{27}H_{31}{}^{79}BrN_2NaO_9S$ [M+Na]$^+$ 661.08385; Found. 661.08457, Calcd. for

$C_{27}H_{32}{}^{81}BrN_2O_9S$ [M+H]$^+$ 641.10233; Found. 641.10552, Calcd. for $C_{27}H_{32}{}^{79}BrN_2O_9S$ [M+H]$^+$ 639.10252; Found. 639.10385.

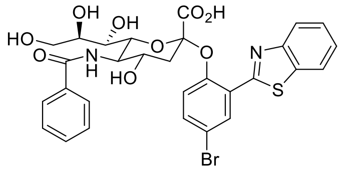

[2-(benzo[*d*]thiazol-2-yl)-4-bromophenyl]-*N*-benzoyl-α-D-neuraminic acid (S5i, Compound 3)

Yield: 9.0% (10.2 mg, colorless amorphous)

^{1}H-NMR (600 MHz, D_2O) δ 7.93 (s, 1 H), 7.82-7.81 (m, 2 H), 7.63 (d, *J* = 8.0 Hz, 2 H), 7.46 (t, *J* = 7.4 Hz, 1 H), 7.40-7.33 (m, 4 H), 7.28 (t, *J* = 7.6 Hz, 1 H), 7.20 (d, *J* = 8.8 Hz, 1 H), 4.06 (t, *J* = 10.1 Hz, 1 H), 3.97 (d, *J* = 10.5 Hz, 1 H), 3.79 (td, *J* = 10.8, 4.7 Hz, 1 H), 3.74-3.69 (m, 2 H), 3.53 (d, *J* = 9.0 Hz, 1 H), 3.49 (dd, *J* = 11.7, 5.9 Hz, 1 H), 2.88 (dd, *J* = 12.5, 4.5 Hz, 1 H), 2.03 (t, *J* = 12.1 Hz, 1 H).

MS (ESI$^+$) *m/z* 683 [M+Na]$^+$, 681 [M+Na]$^+$, 661 [M+H]$^+$, 659 [M+H]$^+$.

HRMS (ESI-TOF): *m/z* Calcd. for $C_{29}H_{27}{}^{81}BrN_2NaO_9S$ [M+Na]$^+$ 683.04979; Found. 683.05295, Calcd. for $C_{29}H_{27}{}^{79}BrN_2NaO_9S$ [M+Na]$^+$ 681.05183; Found. 681.05176, Calcd. for

$C_{29}H_{28}{}^{81}BrN_2O_9S$ $[M+H]^+$ 661.06784; Found. 661.07017, Calcd. for $C_{29}H_{28}{}^{79}BrN_2O_9S$ $[M+H]^+$ 659.06989; Found. 659.07179.

III. Reference

SR1. Minami, A. et al. Visualization of sialidase activity in Mammalian tissues and cancer detection with a novel fluorescent sialidase substrate. *PLoS One* **9**, e81941 (2014). doi: 10.1371/journal.pone.0081941.

IV. Spectroscopic DATA

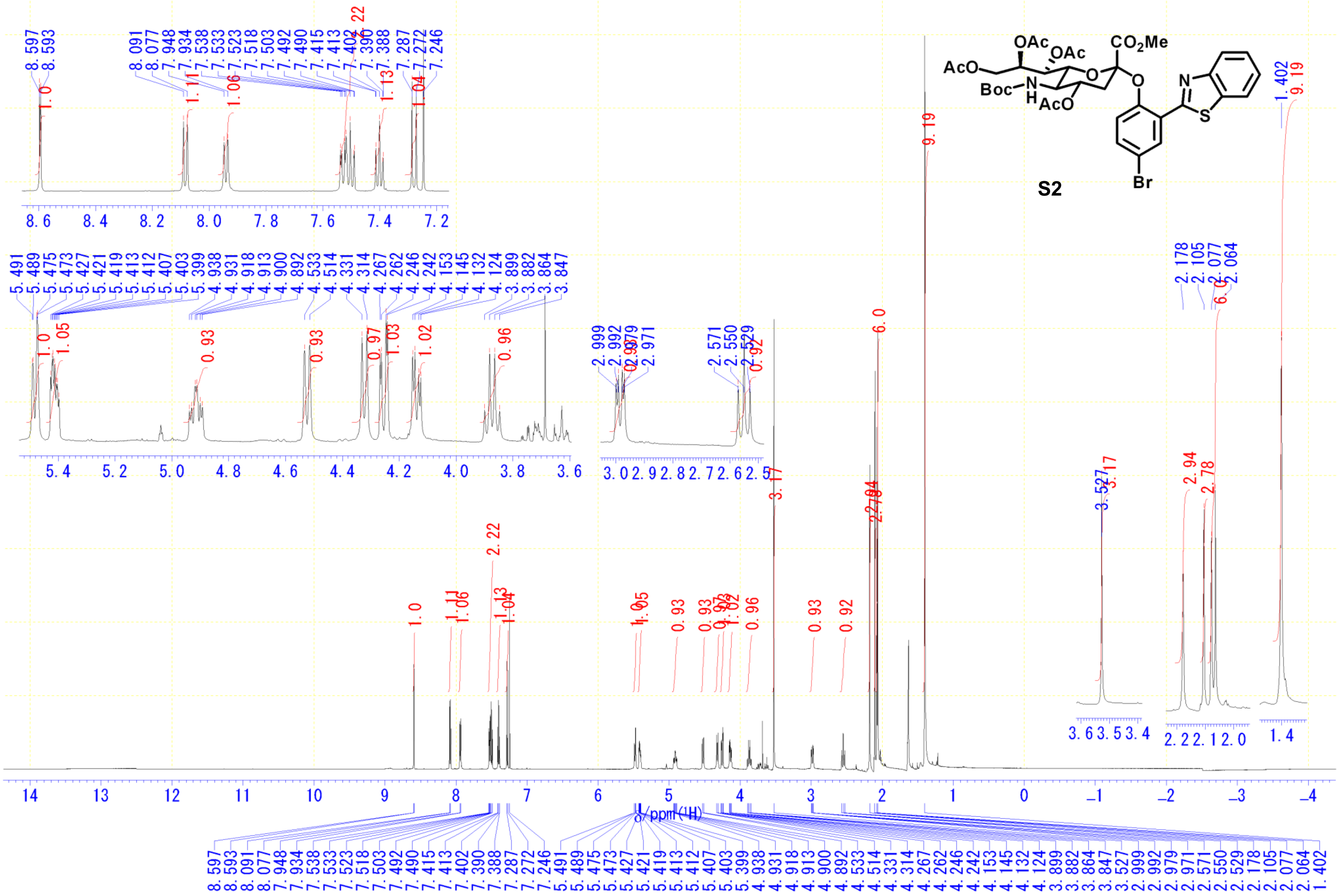

AcO
OAc
OAc
CO2Me
Boc
N
H
AcO
O
O
N
S
Br
S2
δ/ppm (1H)

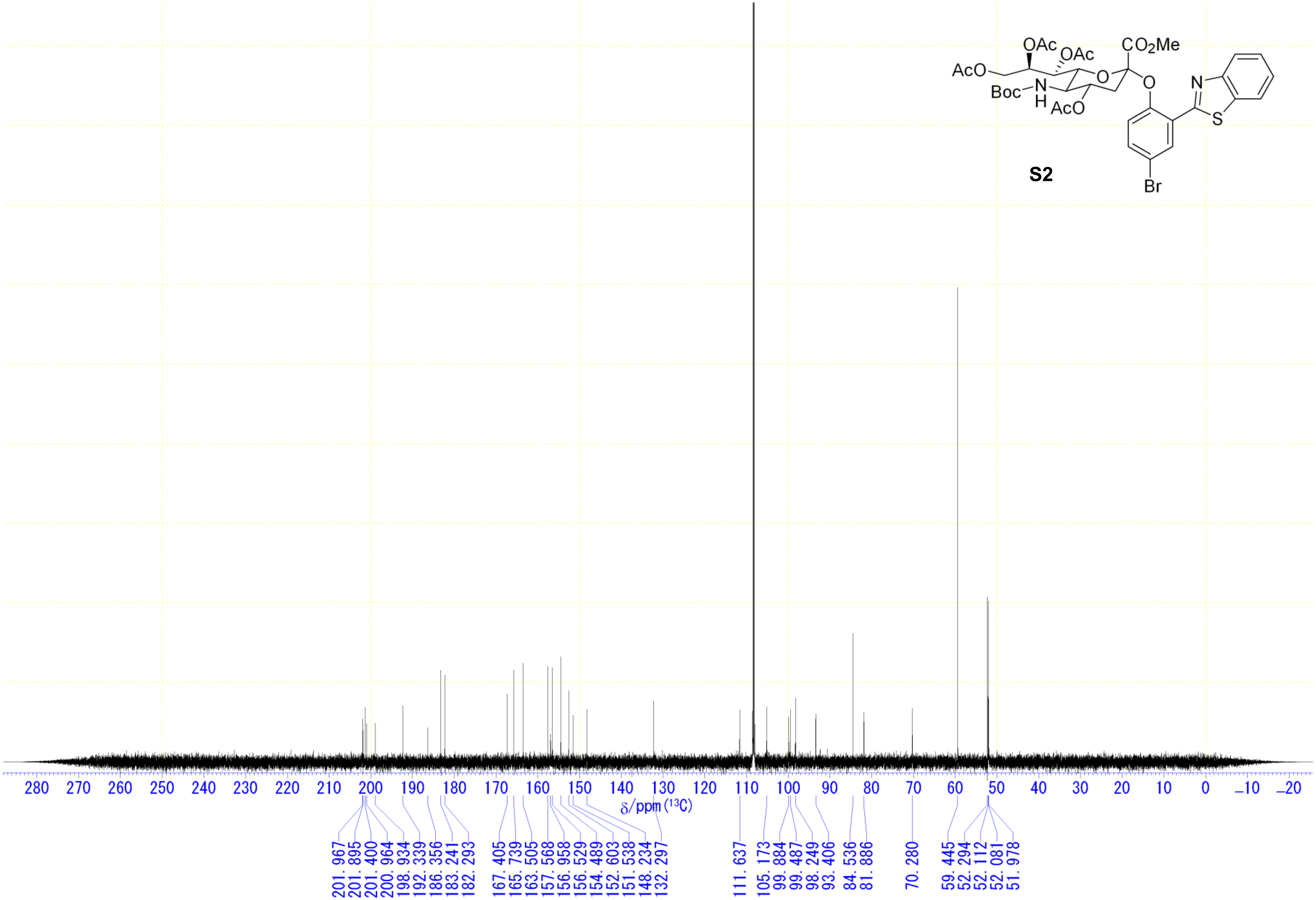
AcO
OAc
OAc
CO2Me
Boc
N
H
AcO
O
O
N
S
Br
S2
280 270 260 250 240 230 220 210 200 190 180 170 160 150 140 130 120 110 100 90 80 70 60 50 40 30 20 10 0 –10 –20
δ/ppm (13C)
201.967
201.895
201.400
200.964
198.934
192.339
186.356
183.241
182.293
167.405
165.739
163.505
157.568
156.958
156.529
154.489
152.603
151.538
148.234
132.297
111.637
105.173
99.884
99.487
98.249
93.406
84.536
81.886
70.280
59.445
52.294
52.112
52.081
51.978

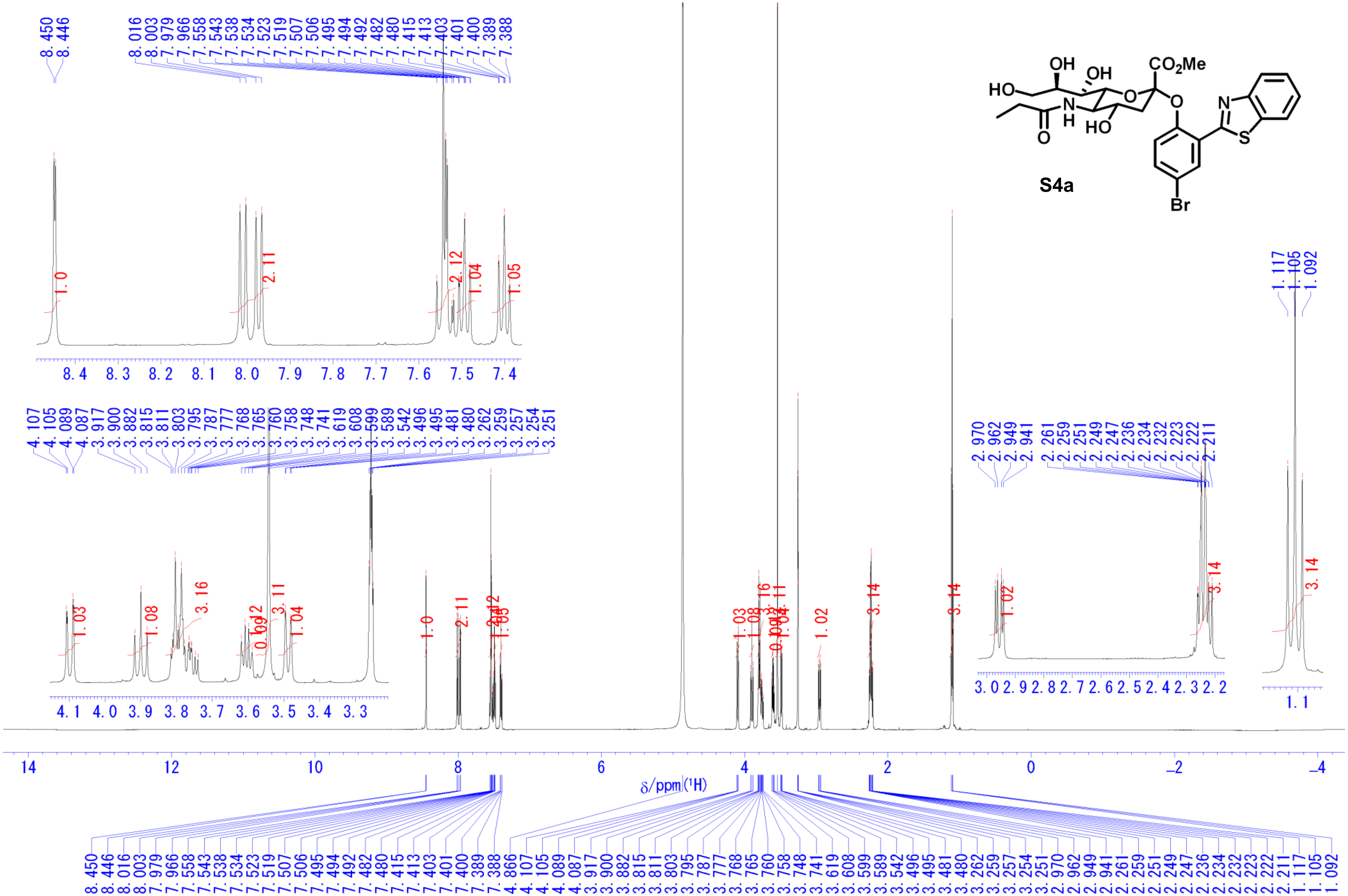

HO
OH
OH
CO2Me
N
H
O
HO
O
N
S
Br
S4a
δ/ppm (1H)
14 12 10 8 6 4 2 0 −2 −4
8.4 8.3 8.2 8.1 8.0 7.9 7.8 7.7 7.6 7.5 7.4
4.1 4.0 3.9 3.8 3.7 3.6 3.5 3.4 3.3
3.0 2.9 2.8 2.7 2.6 2.5 2.4 2.3 2.2
1.1
1.0
2.11
2.12
1.04
1.05
1.03
1.08
3.16
3.11
1.04
1.02
3.14
3.14
8.450 8.446 8.016 8.003 7.979 7.966 7.558 7.543 7.538 7.534 7.523 7.519 7.507 7.506 7.495 7.494 7.492 7.482 7.480 7.415 7.413 7.403 7.401 7.400 7.389 7.388 4.866 4.107 4.105 4.089 4.087 3.917 3.900 3.882 3.815 3.811 3.803 3.795 3.787 3.777 3.768 3.765 3.760 3.758 3.748 3.741 3.619 3.608 3.599 3.589 3.542 3.496 3.495 3.481 3.480 3.262 3.259 3.257 3.254 3.251 2.970 2.962 2.949 2.941 2.261 2.259 2.251 2.249 2.247 2.236 2.234 2.232 2.223 2.222 2.211 1.117 1.105 1.092

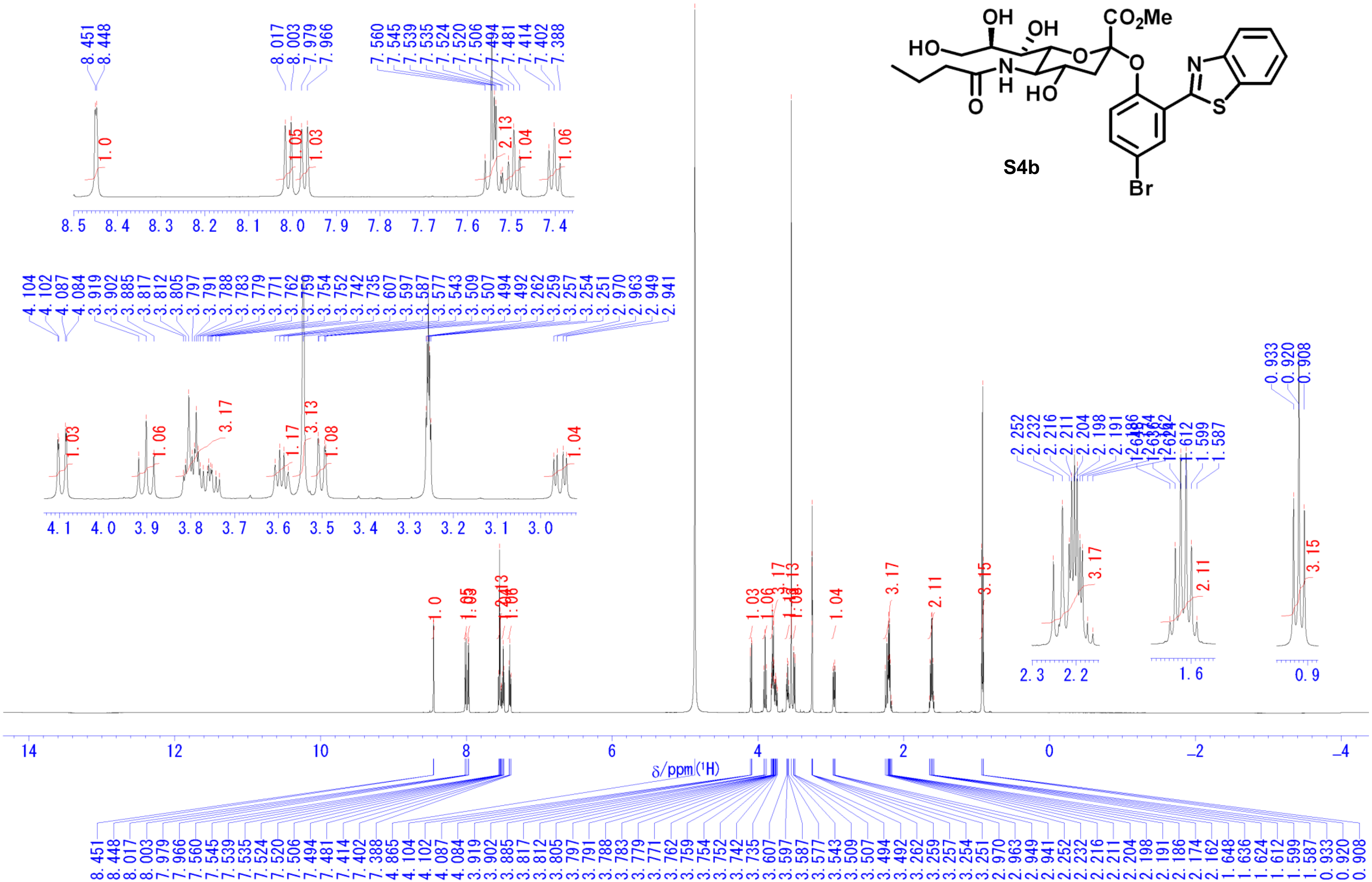
OH
OH
CO2Me
HO
N
H
O
HO
O
N
S
Br
S4b
δ/ppm (1H)
14
12
10
8
6
4
2
0
–2
–4

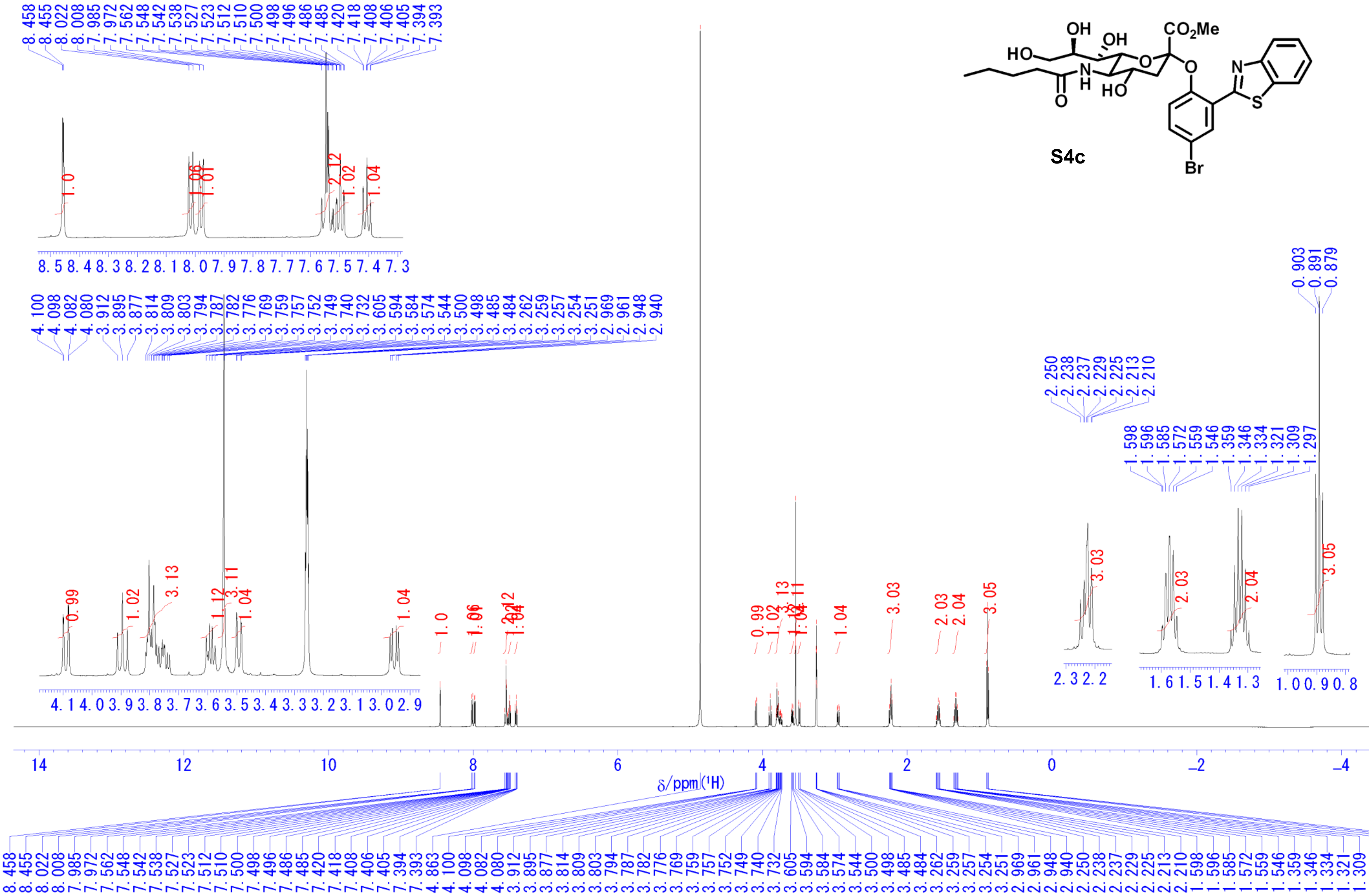

S4c
CO2Me
Br
δ/ppm (¹H)

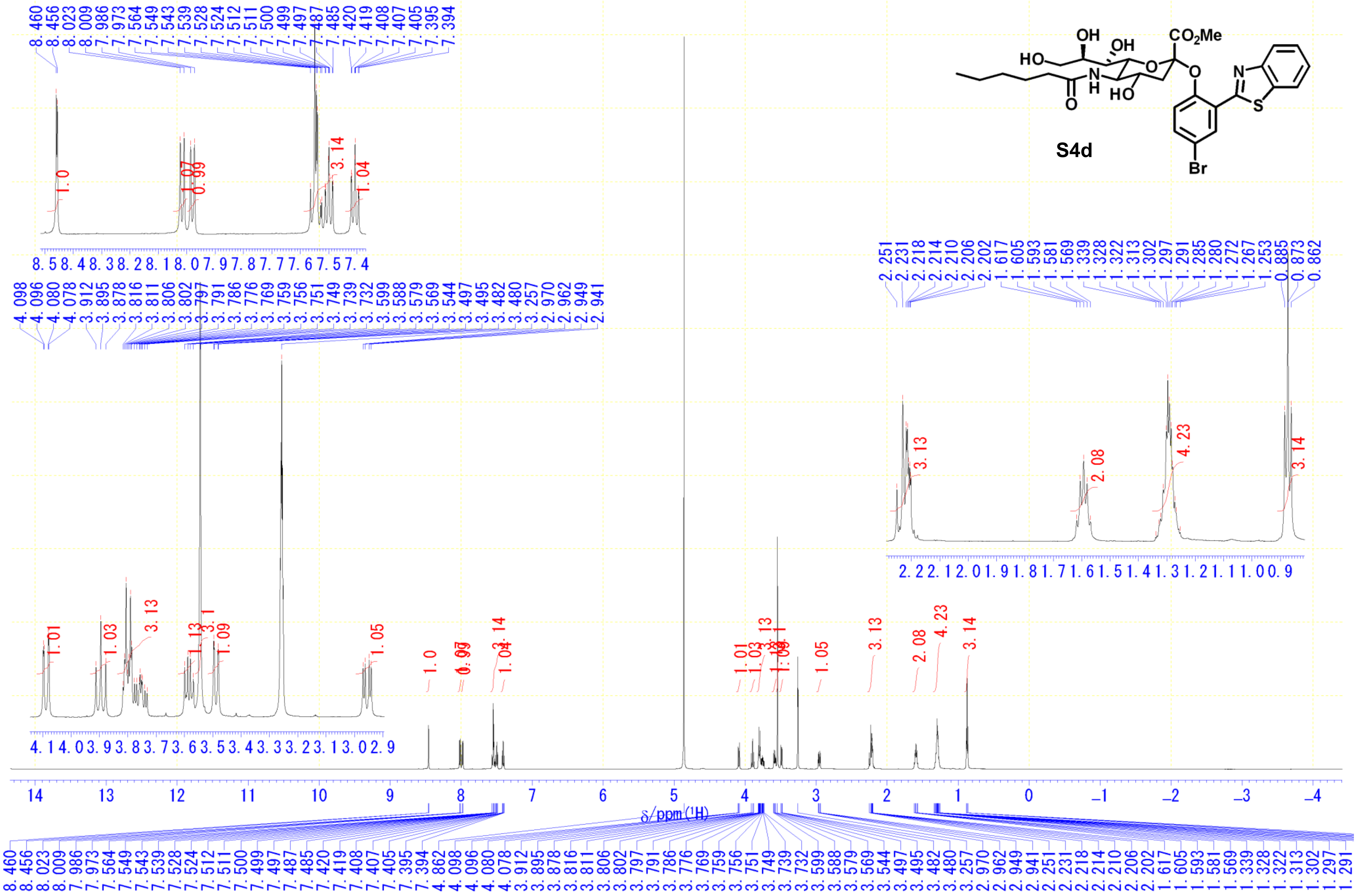
S4d
CO2Me
OH
HO
Br
δ/ppm (1H)

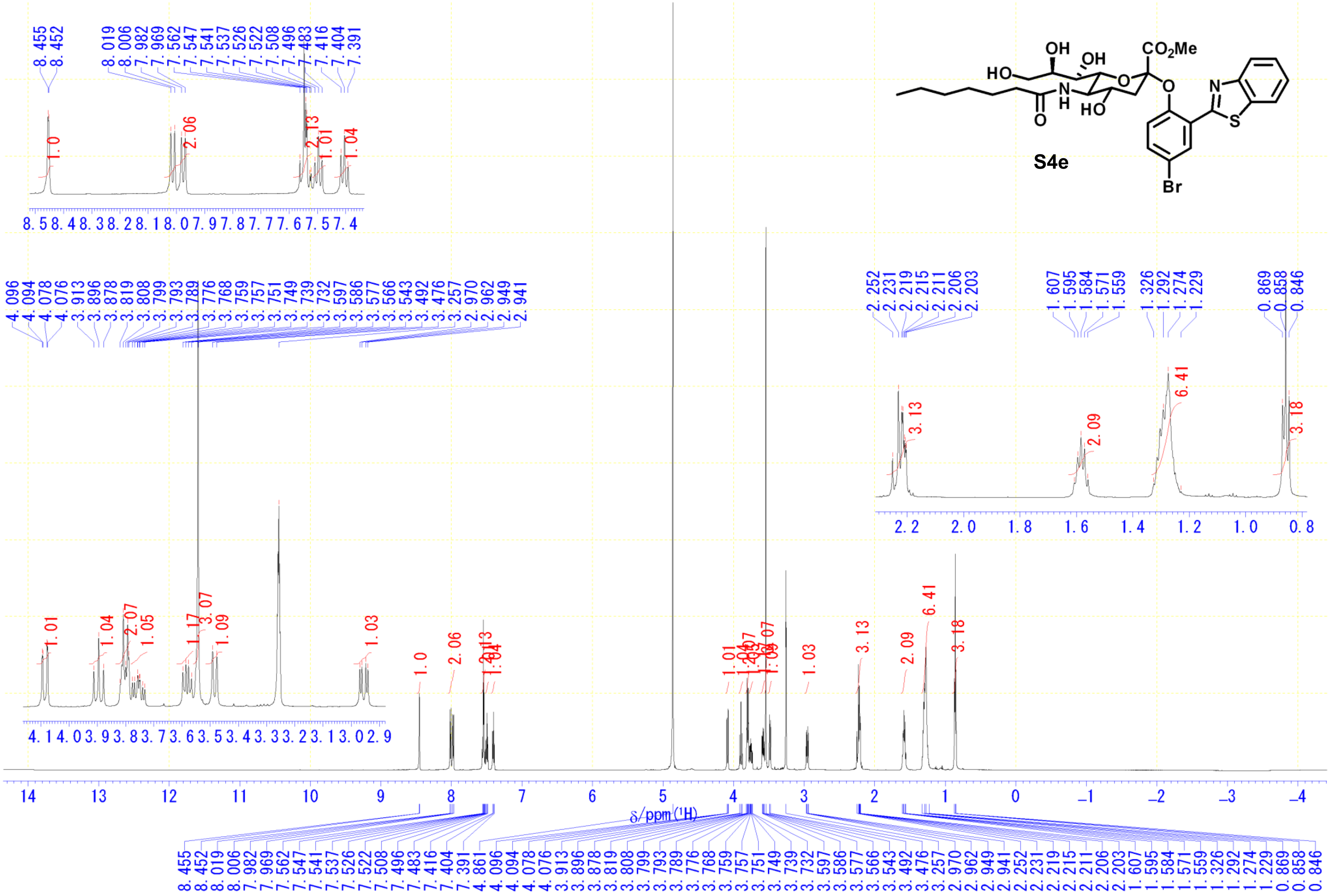
S4e
δ/ppm (¹H)

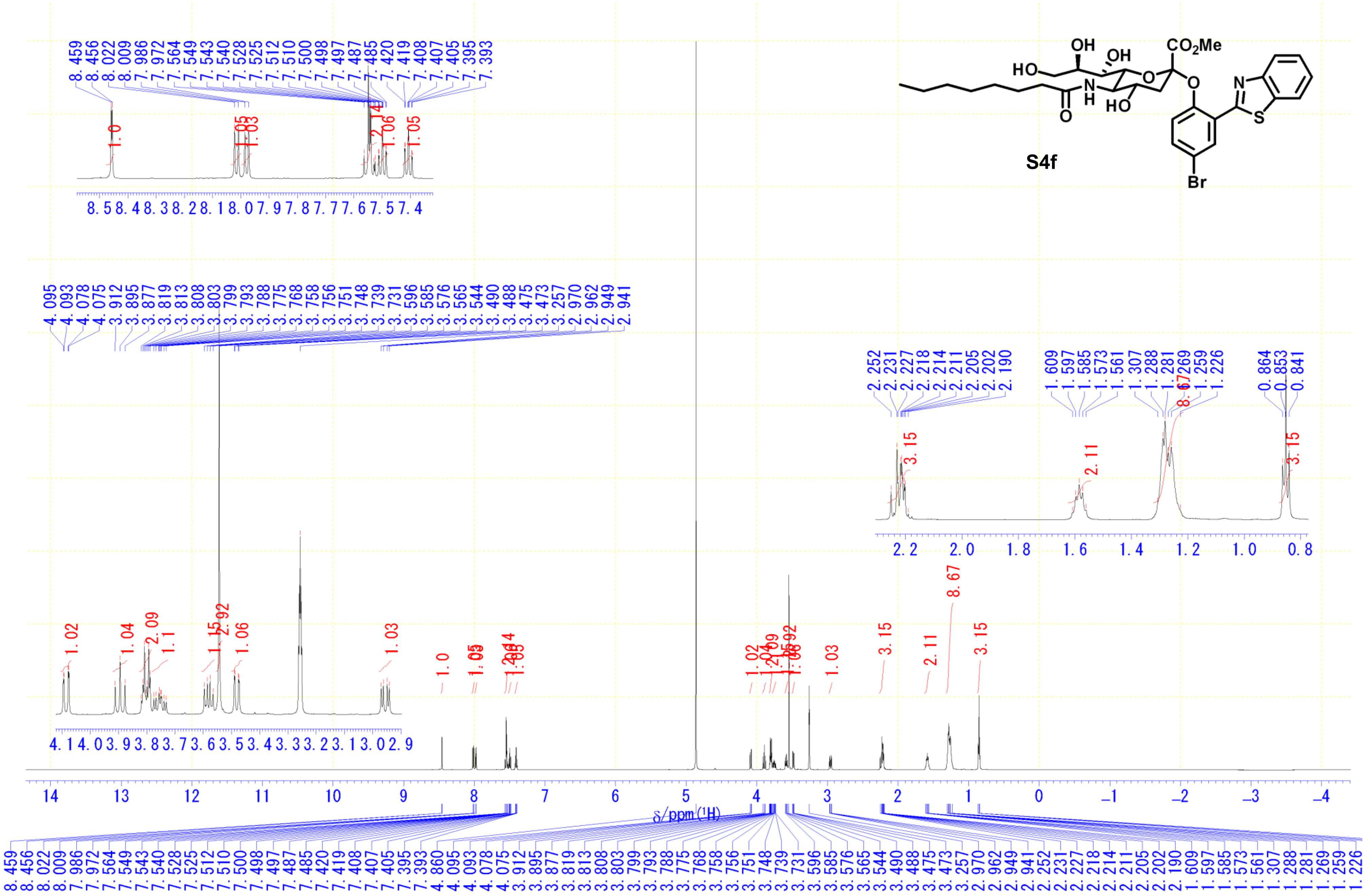
S4f
OH
OH
HO
CO2Me
N
H
O
O
HO
N
S
Br
δ/ppm (1H)

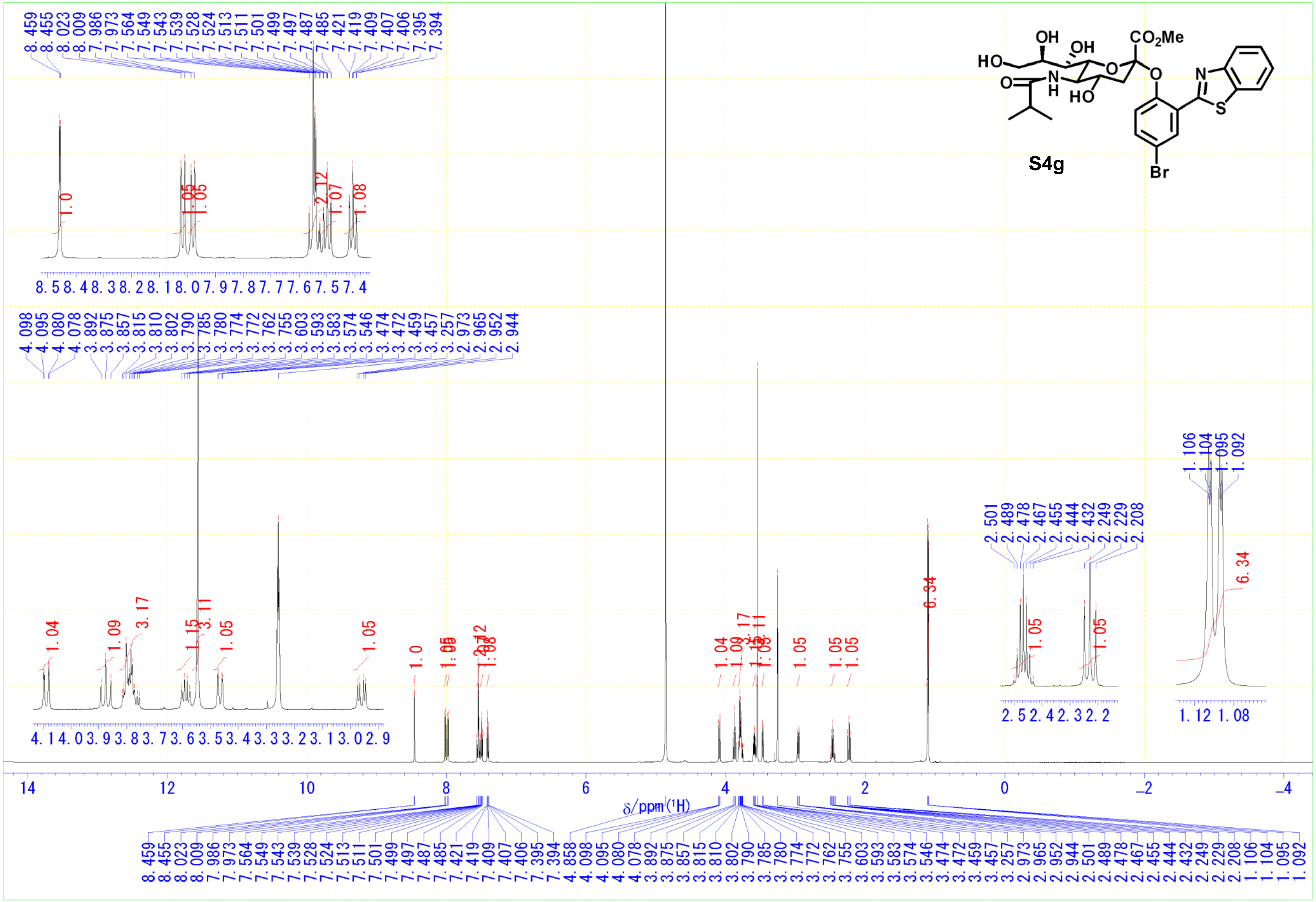
S4g
HO
OH
OH
CO2Me
O
N
H
HO
Br
S
8.459 8.455 8.023 8.009 7.986 7.973 7.564 7.549 7.543 7.539 7.528 7.524 7.513 7.511 7.501 7.499 7.497 7.487 7.485 7.421 7.419 7.409 7.407 7.406 7.395 7.394 4.858 4.098 4.095 4.080 4.078 3.892 3.875 3.857 3.815 3.810 3.802 3.790 3.785 3.780 3.774 3.772 3.762 3.755 3.603 3.593 3.583 3.574 3.546 3.474 3.472 3.459 3.457 3.257 2.973 2.965 2.952 2.944 2.501 2.489 2.478 2.467 2.455 2.444 2.432 2.249 2.229 2.208 1.106 1.104 1.095 1.092
δ/ppm (1H)

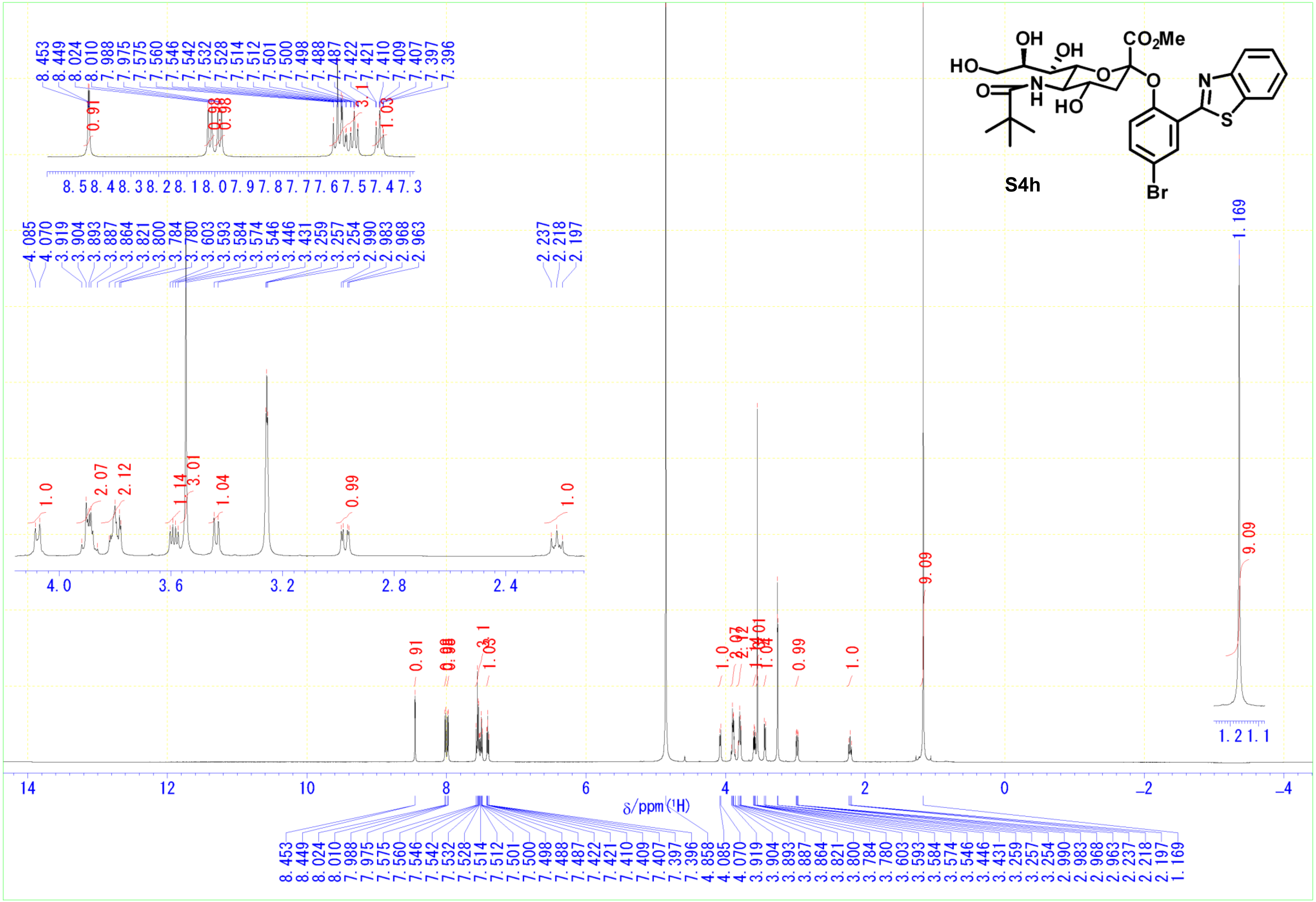
S4h
HO
OH
OH
CO2Me
O
N
H
HO
Br
N
S
δ/ppm (1H)
8.453 8.449 8.024 8.010 7.988 7.975 7.575 7.560 7.546 7.542 7.532 7.528 7.514 7.512 7.501 7.500 7.498 7.488 7.487 7.422 7.421 7.410 7.409 7.407 7.397 7.396 4.858 4.085 4.070 3.919 3.904 3.893 3.887 3.864 3.821 3.800 3.784 3.780 3.603 3.593 3.584 3.574 3.546 3.446 3.431 3.259 3.257 3.254 2.990 2.983 2.968 2.963 2.237 2.218 2.197 1.169
0.91 0.98 0.98 3.1 1.03
1.0 2.07 2.12 1.14 3.01 1.04 0.99 1.0
9.09

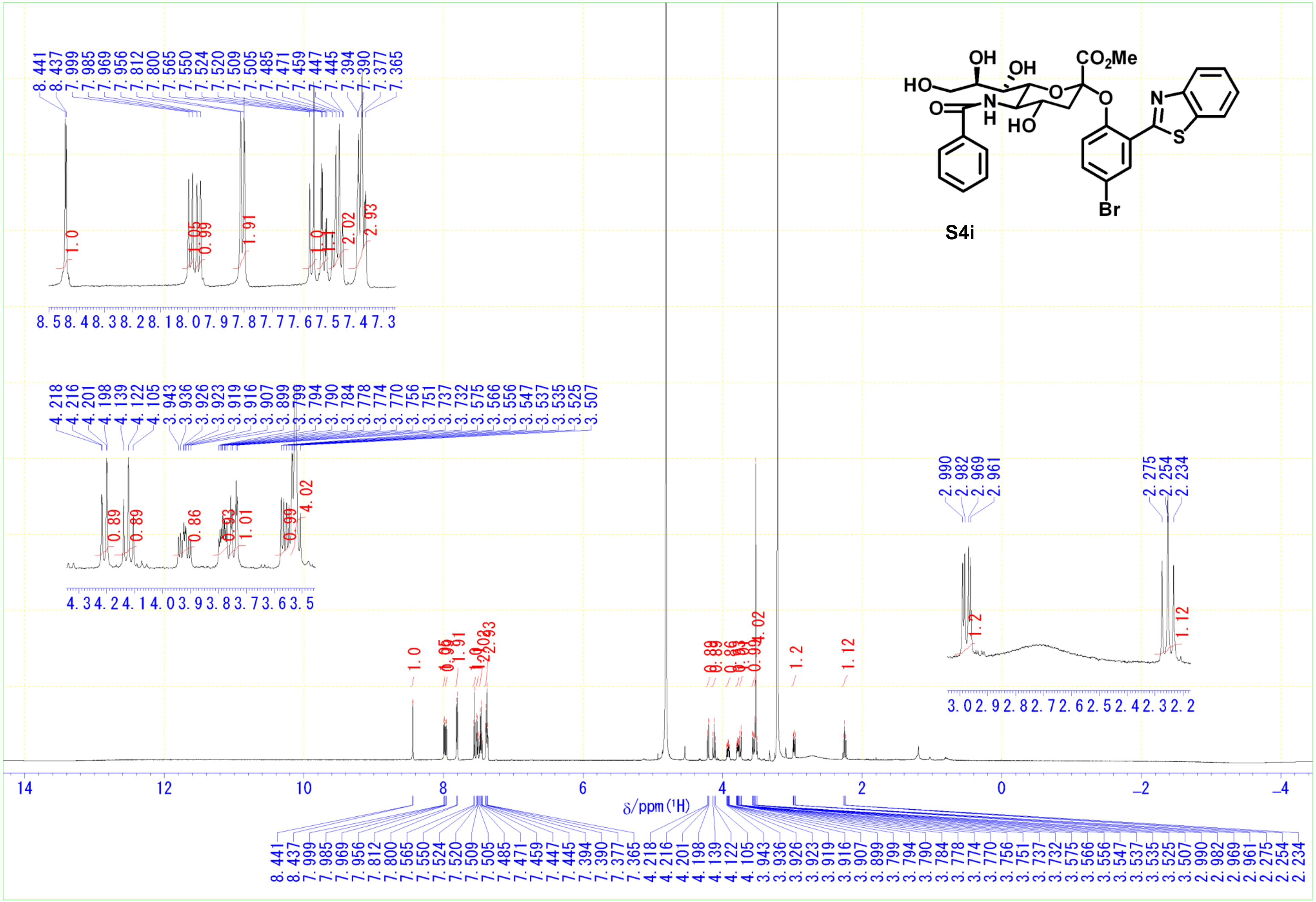
S4i
δ/ppm (¹H)

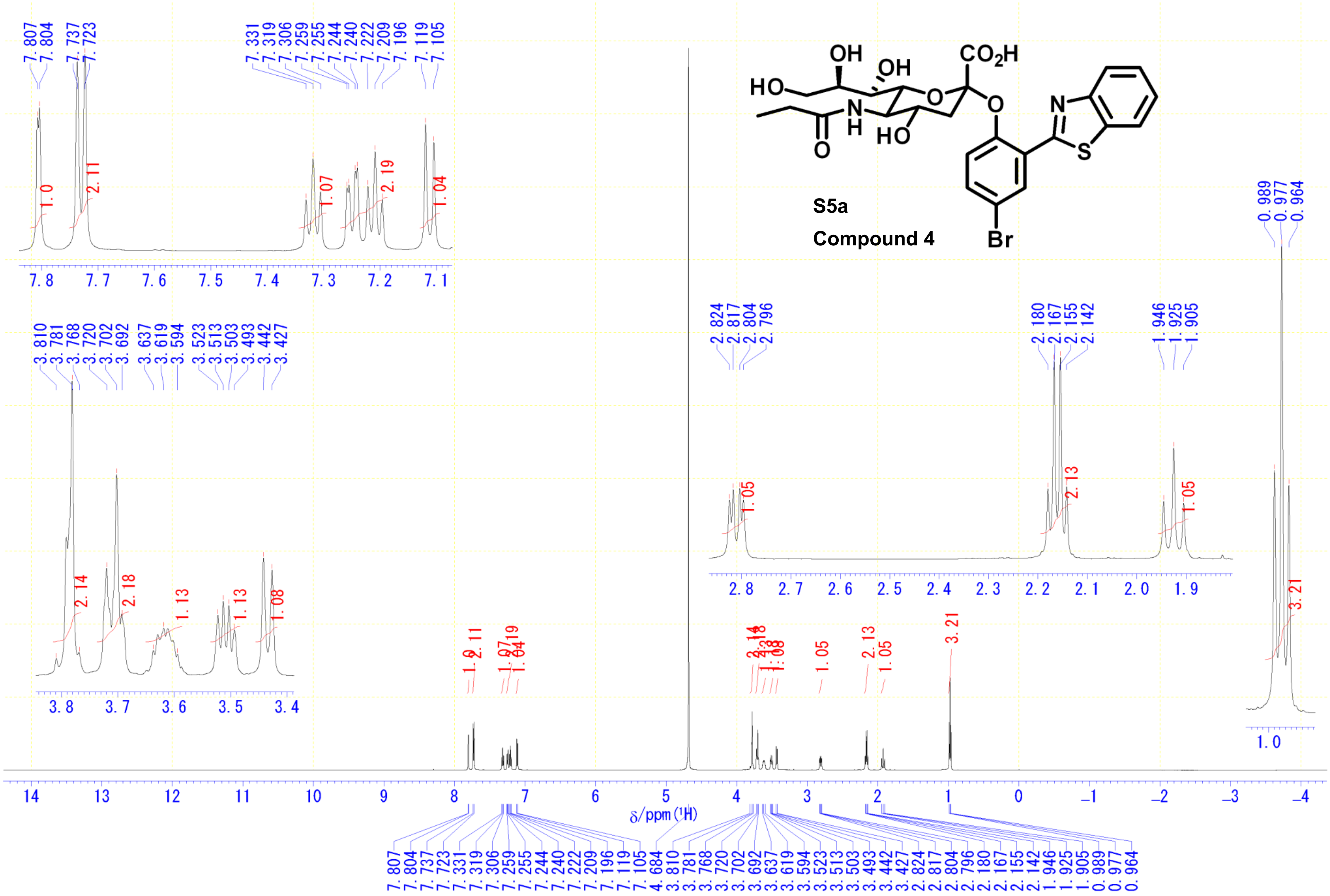
S5a
Compound 4
δ/ppm (¹H)

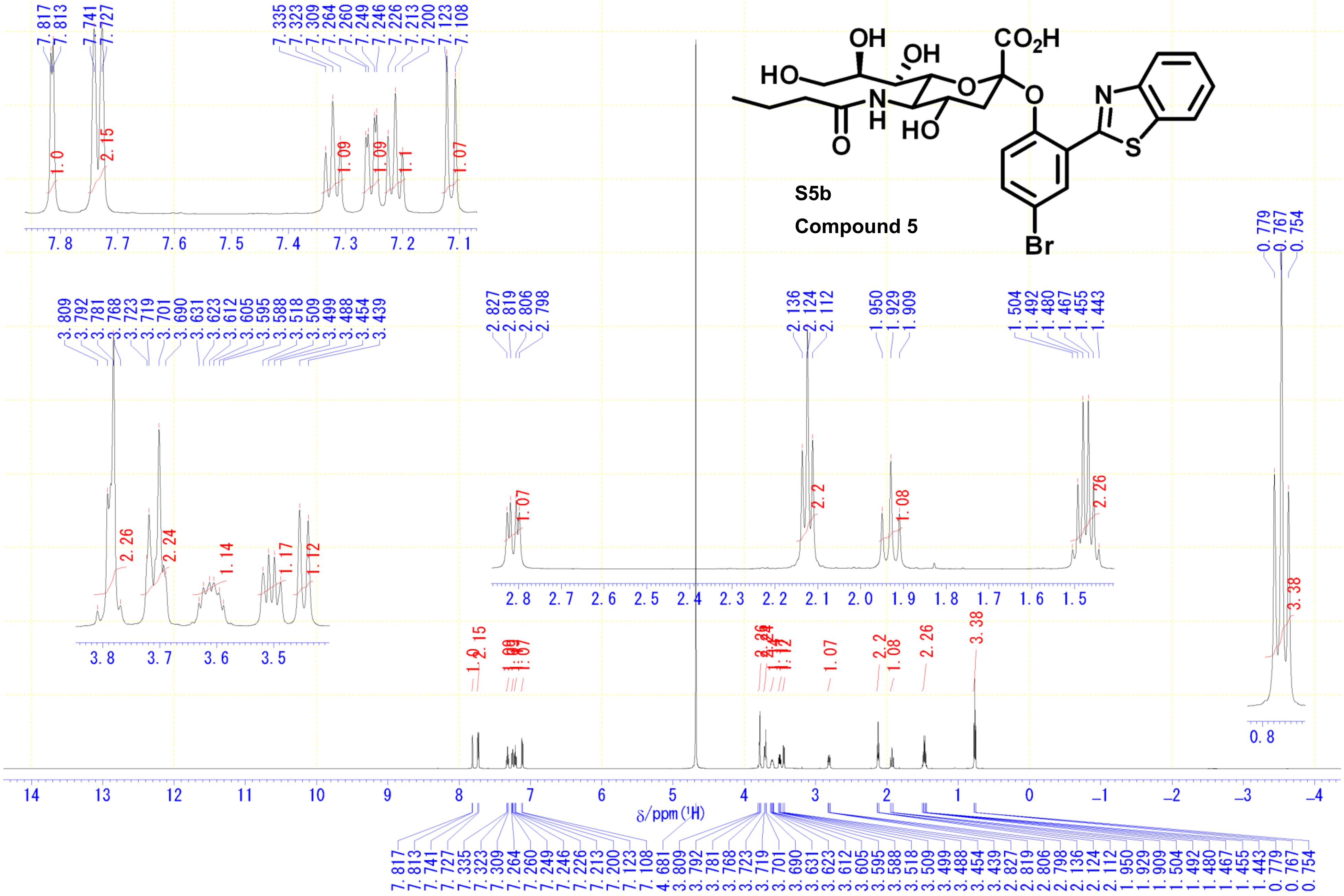

S5b

Compound 5

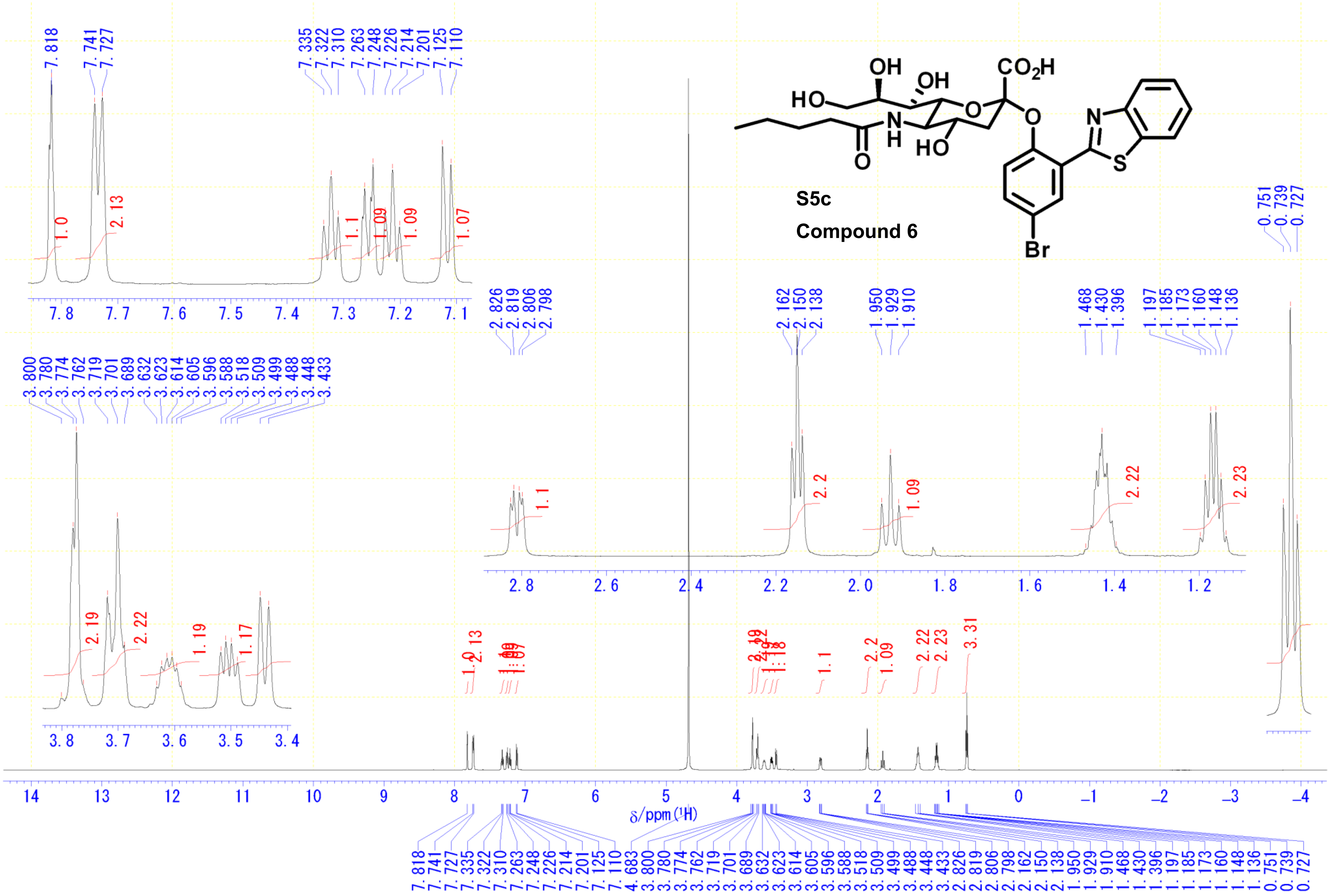
S5c
Compound 6
δ/ppm (¹H)
HO
OH
OH
CO₂H
O
N
H
HO
O
N
S
Br

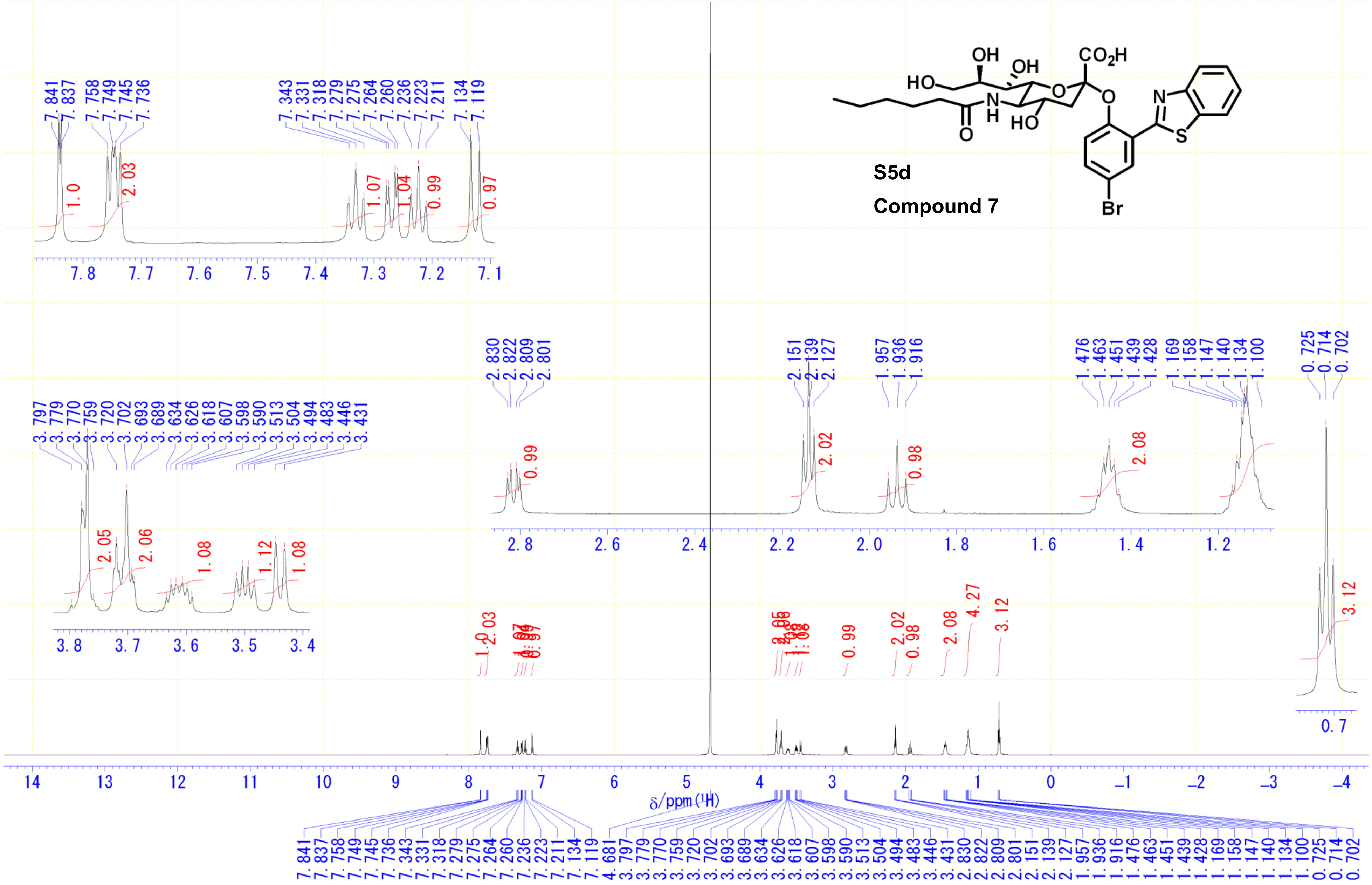
S5d
Compound 7
δ/ppm (¹H)

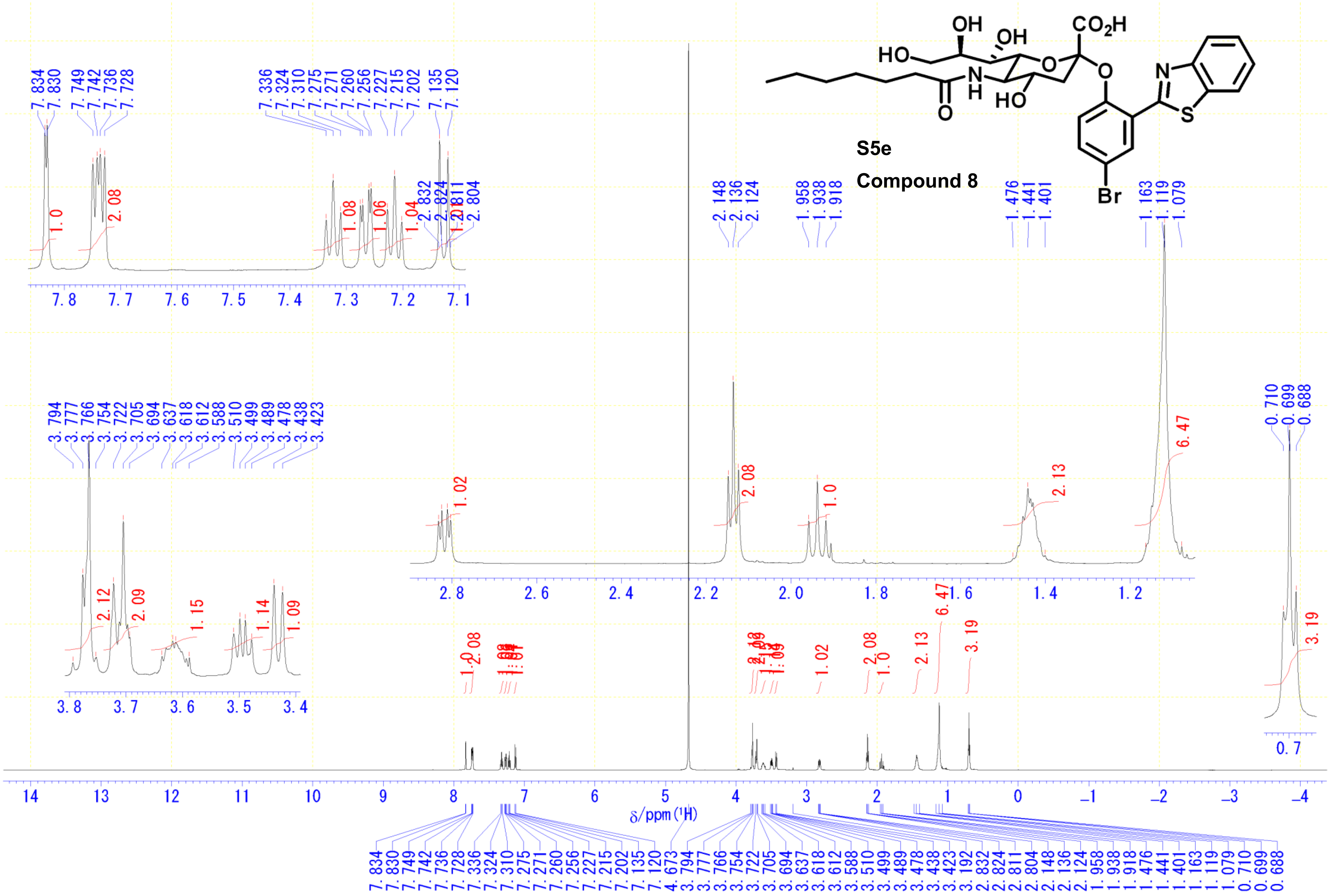
S5e
Compound 8
δ/ppm (¹H)

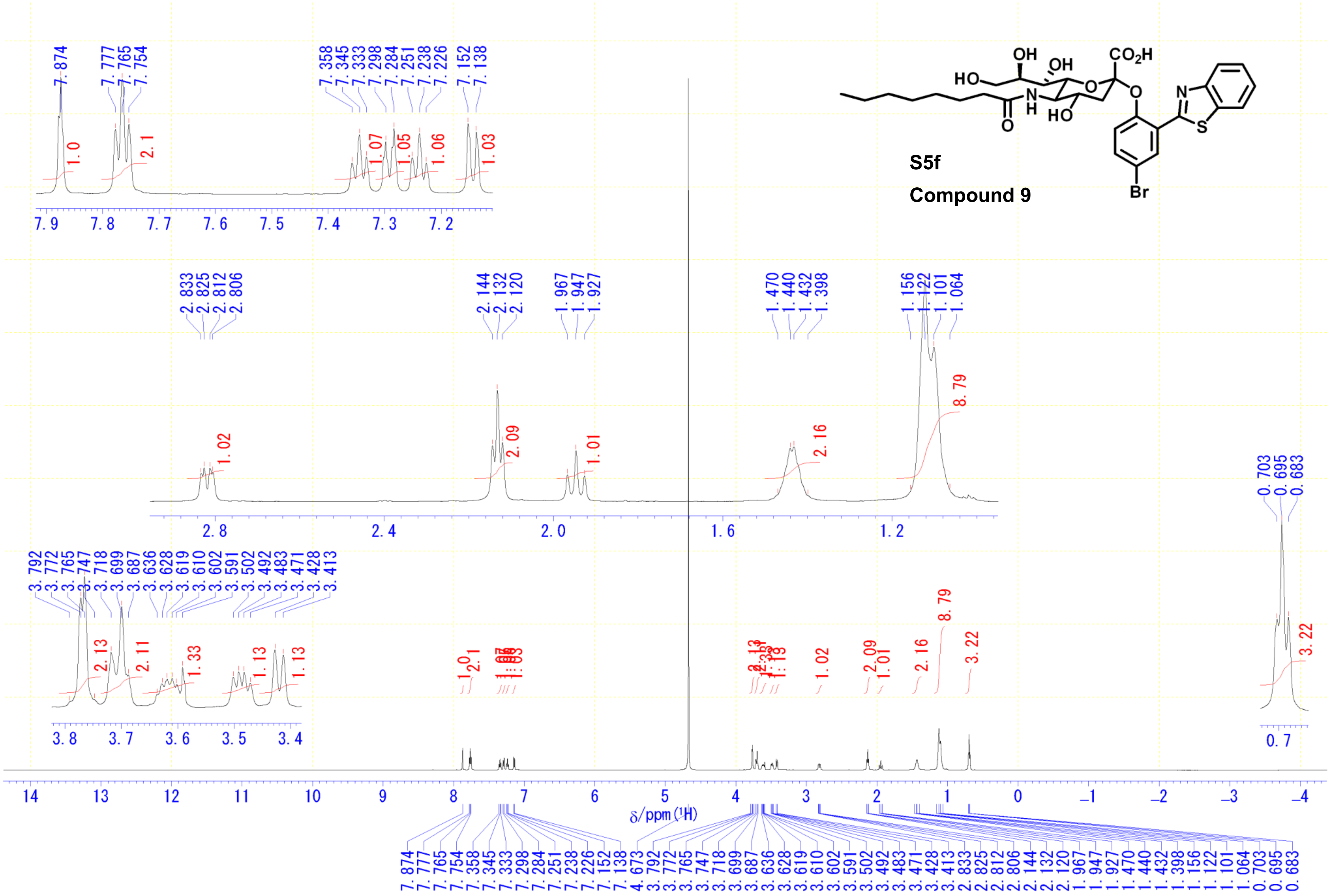
S5f
Compound 9
δ/ppm (1H)
7.874 7.777 7.765 7.754 7.358 7.345 7.333 7.298 7.284 7.251 7.238 7.226 7.152 7.138 4.673 3.792 3.772 3.765 3.747 3.718 3.699 3.687 3.636 3.628 3.619 3.610 3.602 3.591 3.502 3.492 3.483 3.471 3.428 3.413 2.833 2.825 2.812 2.806 2.144 2.132 2.120 1.967 1.947 1.927 1.470 1.440 1.432 1.398 1.156 1.122 1.101 1.064 0.703 0.695 0.683

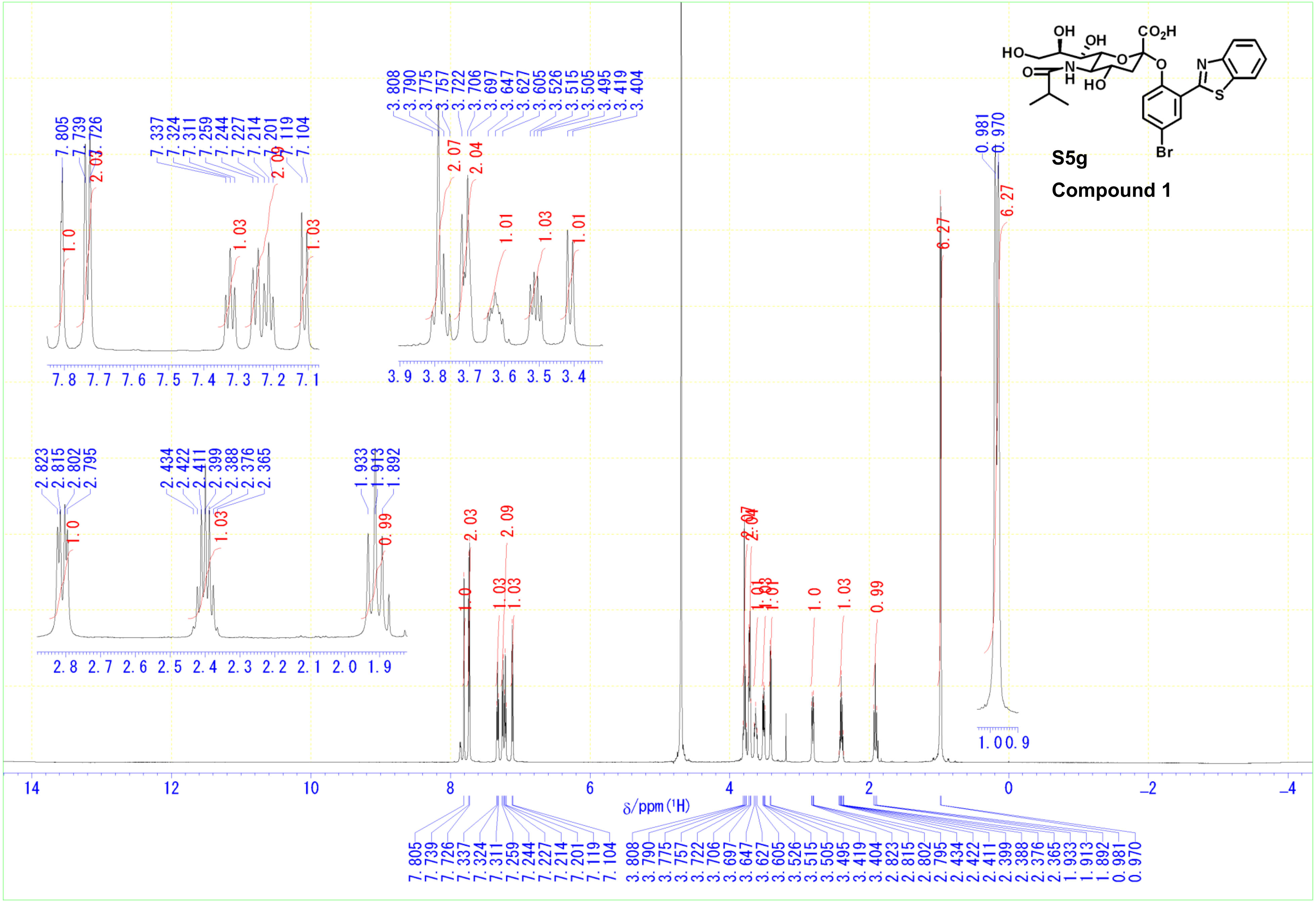

S5g
Compound 1
δ/ppm (¹H)

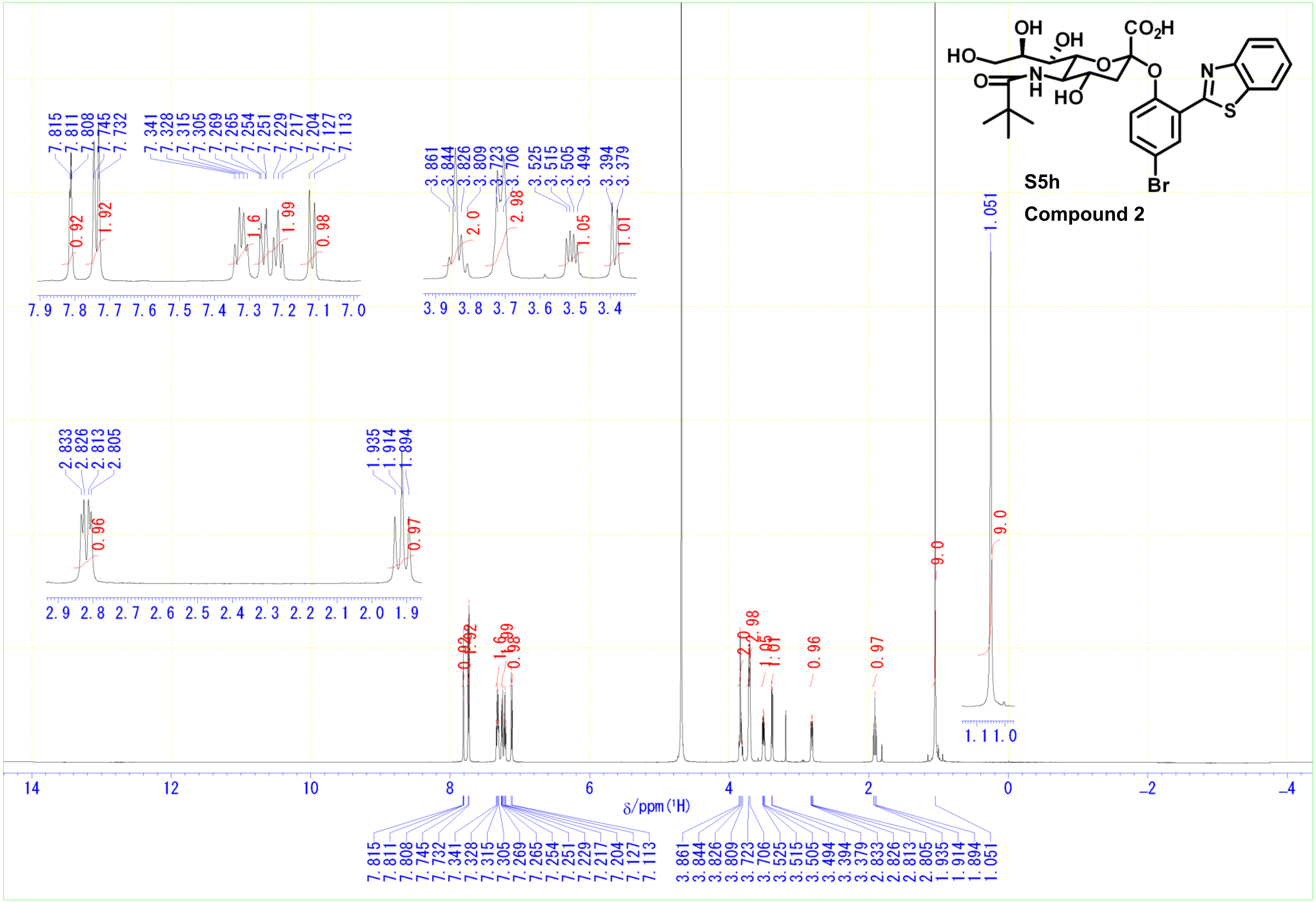
S5h
Compound 2
δ/ppm (1H)
7.815 7.811 7.808 7.745 7.732 7.341 7.328 7.315 7.305 7.269 7.265 7.254 7.251 7.229 7.217 7.204 7.127 7.113
3.861 3.844 3.826 3.809 3.723 3.706 3.525 3.515 3.505 3.494 3.394 3.379
2.833 2.826 2.813 2.805 1.935 1.914 1.894 1.051

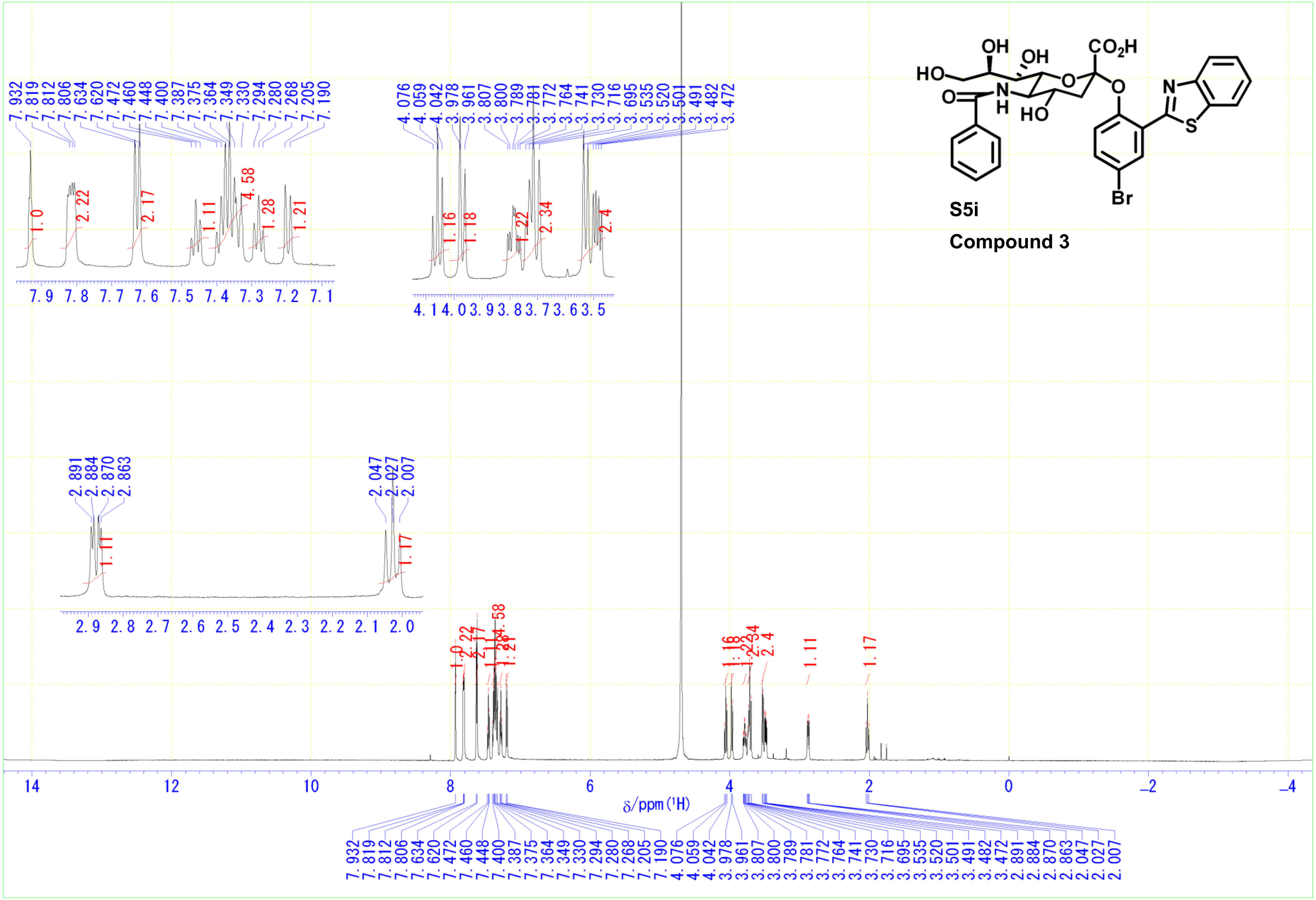
S5i
Compound 3
δ/ppm (¹H)
